# Accurate error control in high-dimensional association testing using conditional false discovery rates

**DOI:** 10.1002/bimj.201900254

**Published:** 2021-03-07

**Authors:** James Liley, Chris Wallace

**Affiliations:** 1 MRC Biostatistics Unit, University of Cambridge, Cambridge, UK; 2 Department of Medicine, Addenbrookes Hospital, University of Cambridge, Cambridge, UK; 3 Cambridge Institute of Therapeutic Immunology and Infectious Disease, Jeffrey Cheah Biomedical Centre, Cambridge Biomedical Campus, Cambridge, UK

**Keywords:** conditional false discovery rate, empirical Bayes, false discovery rate, high-dimensional association study, transcriptome-wide association study, unsupervised learning

## Abstract

High-dimensional hypothesis testing is ubiquitous in the biomedical sciences, and informative covariates may be employed to improve power. The conditional false discovery rate (cFDR) is a widely used approach suited to the setting where the covariate is a set of p-values for the equivalent hypotheses for a second trait. Although related to the Benjamini–Hochberg procedure, it does not permit any easy control of type-1 error rate and existing methods are over-conservative. We propose a newmethod for type-1 error rate control based on identifyingmappings from the unit square to the unit interval defined by the estimated cFDR and splitting observations so that each map is independent of the observations it is used to test. We also propose an adjustment to the existing cFDR estimator which further improves power. We show by simulation that the new method more than doubles potential improvement in power over unconditional analyses compared to existing methods. We demonstrate our method on transcriptome-wide association studies and show that the method can be used in an iterative way, enabling the use of multiple covariates successively. Our methods substantially improve the power and applicability of cFDR analysis.

## 1 Introduction

In the ‘omics’ approach to biology, a large number *n* of descriptive variables are considered in the analysis of a biological system, intended to provide a near-exhaustive characterisation of the system under consideration. Typically only a small proportion of the investigated variables are associated with the behaviour of the system, and we seek to identify this subset of variables, along with the magnitude and direction of their associated effect sizes. A first step is generally to test each hypothesis in a frequentist framework, generating a corresponding set of p-values. Often, additional information is available in the form of an external covariate, which assigns a numerical value to each hypothesis which has different (unknown) distributions amongst associations and non-associations. Information from such covariates can be incorporated into hypothesis testing to improve power in detecting associations.

A range of procedures have been proposed for this type of analysis. An important consideration is the form of the (two-dimensional) rejection rule applied to the p-value-covariate pairs. An optimal procedure (in terms of minimising type 2 error and controlling type 1 error) determines rejection regions on the basis of a ratio of bivariate probability densities (PDFs) of the p-value and covariate under the null and under the alternative. One approach to the problem at hand is to estimate this ratio directly (Alishahi et al., 2016; Du et al., 2014; Lei and Fithian, 2018). Other approaches include ‘filtering’ on covariate values (Bourgon et al., 2010), weighting hypotheses according to the value of the covariate (Basu et al., 2018; Benjamini et al., 2006; Cai et al., 2016; Genovese et al., 2006; Ignatiadis et al., 2016), modulating a univariate test of p-values in response to the covariate in some other way (Li and Barber, 2016, 2017; Scott et al., 2015) and binning covariates in order to treat each bin separately (Ferkingstad et al., 2008). Since covariates can be of many types (continuous, categorical; univariate, multivariate; known or unknown distributional properties) and can relate to the p-values in a range of ways, this array of methods is necessary to manage the range of problem types.

The conditional false discovery rate (cFDR) circumvents the difficulties of estimating PDFs by approximating the optimal ratio using cumulative density functions (CDFs) (Andreassen et al., 2013c). In this case, the covariate is generally a set of p-values arising from an analogous procedure on the same variables for a second ‘conditional’ trait with an unknown degree of similarity to the trait giving rise to the primary set of p-values (whichwe call the ‘principal’ trait). Themethod has been extensively used in genomics (Andreassen et al., 2013a, 2013b, 2013c, 2014a, 2014b, 2015; Broce et al., 2018; Desikan et al., 2015a, 2015b; Ferrari et al., 2017; Karch et al., 2018; Le Hellard et al., 2016; Liley and Wallace, 2015; Liu et al., 2013; Lv et al., 2017; McLaughlin et al., 2017; Schork et al., 2016; Shadrin et al., 2018; Smeland et al., 2017a, 2017b, 2017c; Van der Meer et al., 2020; Wang et al., 2016; Witoelar et al., 2017; Zuber et al., 2018). Formally, the cFDR is a posterior probability of non-association with the principal trait given that p-values for the principal and conditional traits fall below p-value thresholds *p, q,* respectively. It is readily estimated using empirical CDFs (ECDFs) (Andreassen et al., 2013c).

The cFDR is a useful Bayesian quantity in its own right. Generally, the cFDR is used in effectively a frequentist way: roughly, for each observed p-value pair (*p_i_*, *q_i_*), we estimate the cFDR at (*p*, *q*) = (*P_i_*, *q_i_* and reject the null hypothesis if this estimated value is less than some threshold α. This process is nearly analogous to the Benjamini-Hochberg procedure (B-H) (Benjamini and Hochberg, 1995) on a single set of p-values *p_i_*, but unlike B-H, it does not control the false discovery rate (FDR) at α (nor any other conventional measure of type-1 error rate). In a previous paper (Liley and Wallace, 2015), we proposed a rough method to approximately control FDR in this setting, but our method was drastically conservative.

The main contribution of this paper is to propose a much improved type-1 error rate control strategy for cFDR, which improves power relative to previous methods. Our method transforms cFDR estimates into ‘v-values’, which function analogously to p-values and can be used to control FDR or family-wise error rate (FWER). In four secondary contributions, we (a) propose an improvement to the existing estimatorwhich improves power, (b) show several asymptotic results about the method and demonstrate that the effect of certain troublesome properties is small, (c) enable and demonstrate iterative use of the procedure and (d) compare the general cFDR method with PDF-based, parametric and kernel density estimator (KDE)-based approaches. An R package is provided.

In this paper, we begin by describing a motivating example using transcriptome-wide association studies (TWAS). We then summarise the cFDR and its estimator and describe its relation to the B-H procedure. We then describe our method to transform cFDR estimates into p-value-like quantities and discuss how the cFDR approach relates to similar methods in the field. We evaluate the type-1 error rate control and power of the method and finally describe an iterated form of the procedure for use with multiple sets of covariates.

### 1.1 Motivating example

We consider a TWAS (Gusev et al., 2016) of breast cancer BRCA (Michailidou et al., 2017) and ovarian cancer (OCA; Phelan et al., 2017), which are epidemiologically and biologically similar diseases (Greene et al., 1984). TWASs test for association between levels of predicted expression of transcripts (gene products) in various tissues between cases (BRCA or OCA) and controls. For each transcript–tissue pair, theTWAS generates a p-value against the null hypothesis that the predicted mean expression of that transcript in that tissue is the same in case and control populations, according to a transcript-prediction rule learnt from independent data. The TWASs in question test around 10,000 gene transcripts across 45 tissues (though many transcript-tissue pairs are missing), and after we restrict to transcript-tissue pairs common to both studies, we are left with a set of ≈ 10^5^ p-values *PBRCA*, *POCA* for association with BRCA and OCA, respectively (further detail is given in

Supporting Information Section 1.1). We wish to find which of the variables are associated with BRCA, and thus investigate a null hypothesis $H_{BRCA}^{0}$ of non-association. Given established genetic correlations BRCA and OCA, we hope to leverage theOCATWAS results to increase power in this search. We will assume thatwe have no prior knowledge that any variables aremore likely to be BRCA- or OCA- associated, that absolute Z-scores *zBRCA* = −Φ^−1^(*PBRCA*/2), zoca = −Φ^−1^(*Poca*/2) have a block-diagonal correlation structure where block locations are known, and that under a null hypothesis $H_{BRCA}^{0}$ of no association with BRCA, z_*BRCA*_ and z_*OCA*_ is independent.

**Figure 1:**
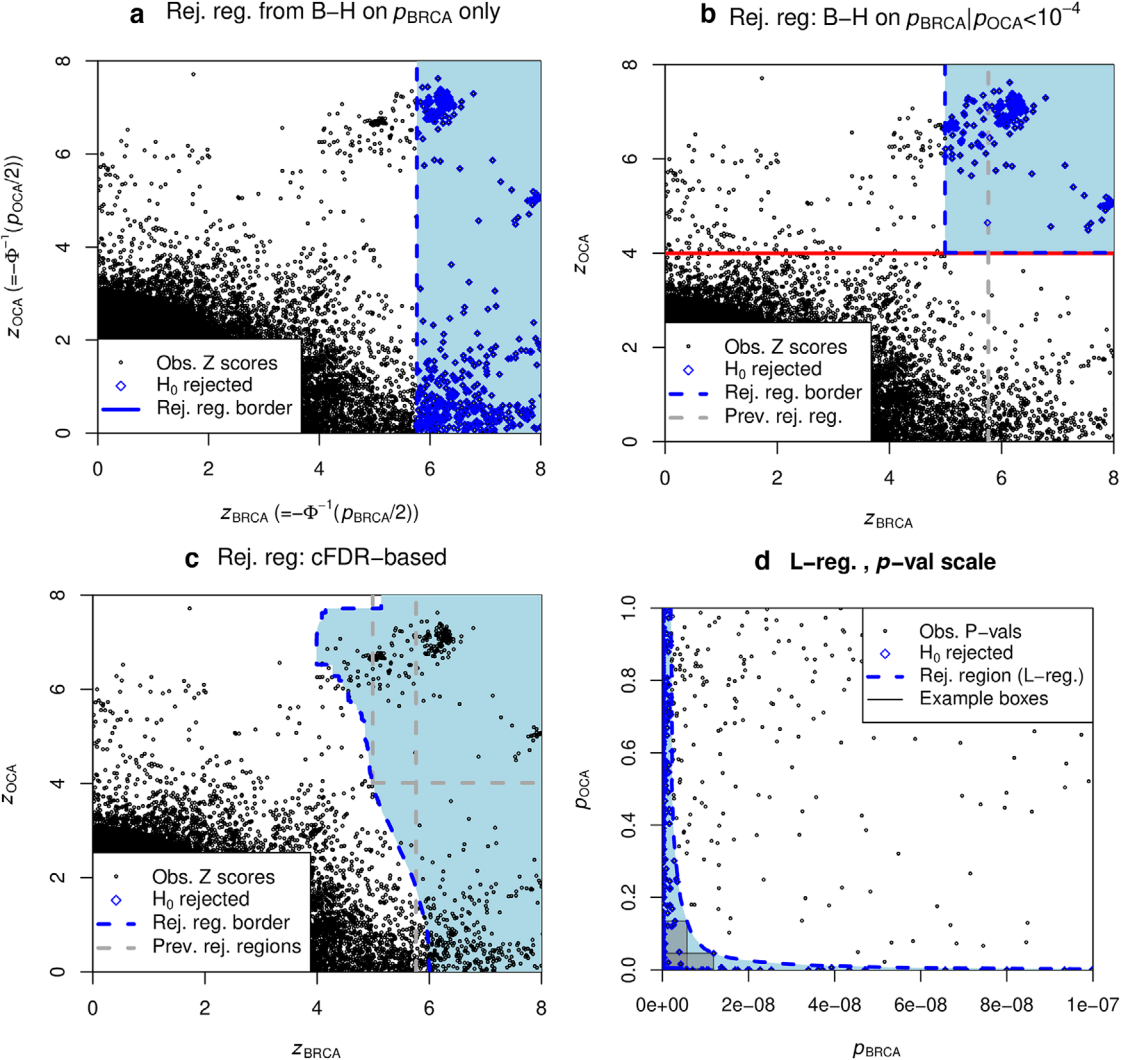
Illustration of cFDR approach using example data from the TWAS study of breast cancer (BRCA), conditioning on ovarian cancer (OCA). Each plot shows test statistics from BRCA (*x* axis) and OCA (*y* axis) on either the Z (a-c) or p-value (d) scale. All rejection regions use methods to control FDR at < 1 × 10^–6^. (a) B-H procedure applied to BRCA statistics alone leads to a rejection region to the right of the blue dashed vertical line. (b) B-H applied to those variables for which z_OCA_ exceeds the threshold shown by a solid red line. (c) cFDR procedure: for the *i*th values *z_BRCA_* (*i*)*z_OCA_* (*i*), a B-H procedure aiming to control the FDR at α is conducted on only the variables for which *z_OCA_* ≥ z_OCA_(*i*), and if the *i*th null hypothesis is rejected during this procedure, it is rejected overall. We term the rejection region corresponding to this value α an ‘L-region’ *L*(α), shown as the shaded region. (d) The exposition that follows using p-values rather than Z scores, and so we reproduce the data and *L*(α) on the p-value scale. On this scale, the estimated cFDR at a point *pBRCA*, *Poca* can be considered an estimate of the FDR corresponding to a fixed rejection region given by the box with *pBRCA*, *Poca* as its top-right corner, and the L-region *L*(α) roughly as the locus of top-right corners of boxes with estimated cFDR equal to α. Two such boxes are illustrated in the figure

A straightforward approach is to apply the B-H procedure to the values *PBRCA* (Figure 1, panel a). BRCA and OCA tend to have associations at the same variables, suggesting that a rejection region should reflect this to improve power. A natural way to do this is to only consider those variables for which *z*_OCA_ exceeds some threshold, which allows rejection of $H_{\text{BRCA}}^{0}$ at a looser *z_BRCA_* threshold (Figure 1, panel b). FDR control is maintained under the independence assumption above (Bourgon et al., 2010). However, this procedure is problematic: a threshold on z_OCA_ must be chosen *a priori* and variables with z_OCA_ falling below the red line have $H_{BRCA}^{0}$ retained automatically.

The cFDR procedure circumvents this problem (Figure 1, panel c). The associated rejection region, which we term an L-region, ‘adapts’ to the joint distribution of *z_BRCA_* and *z_OCA_*. For small *α*, the L-region approximates an optimal rejection region (Appendix A.1). A major shortcoming is that although a B-H procedure with FDR controlled at *α* is repeatedly used to generate the L-region, the overall FDR is not controlled at *α*, nor any straightforward function of *α* (Liley andWallace, 2015; Appendix A.2).

In this paper, we demonstrate a straightforward and effective way to control the type-1 error rate (specifically FDR or FWER) in the cFDR procedure. As *α* varies from 0 to 1, the leftmost boundary of the L-region ‘sweeps’ across the entire (+,+) quadrant, and for α_1_ < α_2_, we have *L*(α_1_) ⊆ *L*(α_2_). Thus we can associate each point (*x*,*y*) in the (+,+) quadrant with the smallest L-region containing it, which will generally have (*x*,*y*)on its leftmost border. Loosely, we control FDR by estimating the probability that each point would lie within its associated region under $H_{\text{BRCA}}^{0}$ . We term this the v-value, which has similar properties to a p-value and can be used in the B-H procedure.

Care must be taken when applying rejection rules to the same data on which those rules were determined, so we use a leave-one-out procedure which avoids this problem (Section 3, Appendix A.4). We show that the rejection region generated by the cFDR approximates the best-possible rejection region (Section 2.2, Appendix A.1), and that rejection regions converge reasonably fast as the number of variables under consideration increases (Section A.3). The rejection region is non-parametric, and we show that the cFDR method can outperform parametric methods (Section 5.2). Finally, the v-values may be considered ‘adjusted’ p-values, which enables straightforward iteration of the method with further sets of p-values at the same variables, discussed in Section 5.5.

## 2 Review of cFDR Estimator

### 2.1 Definitions

Assume that we have results from *n* pairs of hypothesis tests against two series of null hypotheses $\left(H_{0}^{p}(i),H_{0}^{q}(i)\right)$ in the form of a set *S* of bivariate p-values *S* = (*P_i_*,*q_i_*), *i* = 1…*n*. In our motivating example, $H_{0}^{P}(i)$ and $H_{0}^{q}(i)$ are non-association of the *i*th tissue-gene pair with BRCA and OCA, respectively. We consider $\left(H_{0}^{p}(i),H_{0}^{q}(i)\right)$ to be realisations of independent Bernoulli random variables $H_{0}^{p},H_{0}^{q}$ satisfying $Pr\left(H_{0}^{p}\right)=\pi_{0},Pr\left(H_{0}^{q}\right)=\pi_{0}^{q}$ , and *p_i_*, *q_i_* to be independent and identically distributed (IID) realisations of random variables *P*, *Q* satisfying:

(1) $$\begin{array}{r} P|H_{0}^{p}\;\;∼\;\;U(0,1)\\ P\;\;⫫\;\;Q|H_{0}^{p} \end{array}$$)

although assumption (1) can be relaxed. We denote

(2) $$\begin{array}{l} F_{0}(p,q)=Pr\left(P\leq p,Q\leq q|H_{0}^{p}\right)=pF_{0}^{q}(q)\\ \;F(p,q)=Pr(P\leq p,Q\leq q) \end{array}$$

(3) $$\begin{array}{l} f_{0}(p,q)=f\left(P=p,Q=q|H_{0}^{p}\right)=f_{0}^{q}(q)\\ \;f(p,q)=f(P=p,Q=q), \end{array}$$

where the separability of (2) and (3) is due to assumption(1).

### 2.2 Optimal procedure

Under $H_{0}^{p}$ , the probability of a random instance of (*P, Q*) falling in a region *R* is *∫_R_* *f*_0_(*p*, *q*)*dpdq*. To find an ideal two-dimensional rejection region for hypothesis testing, we wish to fix this value at a level *α* while maximising the probability *∫_R_* *f*(*P*, *q*)*dpdq*. This optimal region (or one such optimal region) is given by the set of points {(*p*, *q*) : *f*_0_(*p*, *q*)/*f*(*p*, *q*) ≥ *k*_α_} for some *k_α_* (a formal statement and proof are given in Appendix A.1, and this is also shown in various forms in Du et al. (2014), Alishahi et al. (2016) and Lei and Fithian (2018). In equivalent terms, an optimal decision rule for the set *S* would rankp-value pairs according to *f*_0_(*P_i_*, *q_d_*)/f(*P_i_*, *q_i_*) or equivalently $Pr\left(H_{0}^{p}|P=p_{i},Q=q_{i}\right)$ .

A natural approach is to estimate *f_0_* and *f* using a parametric approximation (Du et al., 2014; Lei and Fithian, 2018) or local approximations using KDEs (Alishahi et al., 2016) or splinemodels (Zablocki et al., 2014).However, PDFs are difficult to estimate in general, and there may be little reason to believe parametric assumptions are satisfied; in our motivating example (Figure 1, panels a-c) there is little reason to think that a smooth rejection region would be optimal.

### 2.3 Conditional false discovery rate

The cFDR (Andreassen et al., 2013c) takes an alternative approach of instead ranking points by an estimate of *F*_0_(*p*, *q*)/*F*(*p*, *q*). This estimate is obtained by estimating the monotonically related quantity:

(4) $$cFDR(p,q)=Pr\left(H_{0}^{p}|P\leq p,Q\leq q\right)$$

(5) $$=\frac{Pr\left(P\leq p|H_{0}^{p},Q\leq q\right)}{Pr(P\leq p|Q\leq q)}Pr\left(H_{0}^{p}|Q\leq q\right).$$

Suppose we have a multi-set *X* of p-value pairs (*P_i_*, *q_i_*). If almost all these pairs are IID realisations (*p_i_*, *q_i_*) of (*P*, *Q*), then for fixed *p*, *q*, the ECDFs

$$\begin{array}{r} \frac{1}{\left|X\right|}\left|\left\{i:\left(p_{i},q_{i}\right)\in X,p_{i}\leq p,q_{i}\leq q\right\}\right|\\ \frac{1}{\left|X\right|}\left|\left\{i:\left(p_{i},q_{i}\right)\in X,q_{i}\leq q\right\}\right| \end{array}$$

are consistent estimators of *Pr*(*P* ≤ *p*, *Q* ≤ *q*), Pr(*Q* ≤ *q*), respectively. Given assumption (1), we have $p=Pr\left(P\leq p|H_{0}^{p}\right)=Pr\left(P\leq p|Q\leq q,H_{0}^{p}\right)$ and (for the moment) we may conservatively approximate $Pr\left(H_{0}^{p}|Q\leq q\right)=1$ . Given *X*, we thus define the estimated cFDR $\left(\;\text{denoted}\;\hat{cFDR}\right)$ , as a function of two variables (*p*, *q*) ∈ (0, 1):

(6) $${\hat{cFDR}}_{X}(p,q)=p\frac{\max\left(\left|\left\{i:q_{i}\leq q,\left(p_{i},q_{i}\right)\in X\right\}\right|,1\right)}{\max\left(\left|\left\{i:p_{i}\leq p,q_{i}\leq q,\left(p_{i},q_{i}\right)\in X\right\}\right|\right),1)}.$$

For fixed $p,q,{\hat{cFDR}}_{X}(p,q)$ is a generally biased but consistent estimator of $cFDR(p,q)/Pr\left(H_{0}^{p}|Q\leq q\right)$ , which converges uniformly on fixed regions at a rate of *O*(*n* ^–1/2^) (see Appendix A.3), and it is usually a downwards-biased (conservative) estimator of *cFDR*(*p*, *q*).

Approximating $Pr\left(H_{0}^{p}|Q\leq q\right)=1$ in Equation (6) disregards any variation on $Pr\left(H_{0}^{p}|Q\leq q\right)$ with *q*, so we introduce at this stage an estimate of $Pr\left(H_{0}^{p}|Q\leq q\right)$ , which we can multiply with ${\hat{cFDR}}_{X}(p,q)$ to improve the accuracy of approximation of *cFDR*(*p*, *q*). Our estimate is

(7) $$\begin{array}{lll} Pr\left(H_{0}^{p}|Q\leq q\right) & = & Pr\left(H_{0}^{p}\right)\frac{Pr\left(Q\leq q|H_{0}^{p}\right)}{Pr(Q\leq q)}\\ & \approx & \pi_{0}\frac{Pr(Q\leq q|P>1/2)}{Pr(Q\leq q)}\\ & \approx & \frac{\min\left(1,\left|\left\{i:\left(p_{i},q_{i}\right)\in X,q_{i}\leq q,p_{i}>1/2\right\}\right|\right)}{\min\left(1,\left|\left\{i:\left(p_{i},q_{i}\right)\in X,q_{i}\leq q\right\}\right|\left|\left\{i:\left(p_{i},q_{i}\right)\in X,p_{i}>1/2\right\}\right|\right)}\\ & = & {\hat{Pr}}_{X}\left(H_{0}^{p}|Q\leq q\right), \end{array}$$

where we approximate $\pi_{0}=Pr\left(H_{0}^{P}\right)=1$ . We denote

(8) $${\hat{cFDR}}_{X}^{n}(p,q)={\hat{cFDR}}_{X}(p,q){\hat{Pr}}_{X}\left(H_{0}^{p}|Q\leq q\right).$$

Estimating *π*_0_ (rather than setting *π*_0_ = 1) would uniformly scale all estimates of *cFDR*(*p*, *q*), which has no effect on our rejection procedure.

In the hypothesis testing setting, we aim to use $\hat{cFDR}$ or ${\hat{cFDR}}^{n}$ to construct a decision rule on our set *S* of observed p-value pairs (we will forego the *n* superscript from now, with the understanding that itmay be added). A simple approach is to reject $H_{0}^{p}(i)$ if ${\hat{cFDR}}_{S}\left(p_{i},q_{i}\right)\leq \alpha$ , but ${\hat{cFDR}}_{S}(p,q)$ is notmonotonically increasing with *p* andwe do not want to reject $H_{0}^{p}$ for some (*P_i_*, *q_i_*) and not for some other pair (*P_j_*, *q_j_*) with *q_i_* = *q_j_* but *p_j_* < *p_i_*. Hence, we can use the decision rule (as per Andreassen et al., 2013c)

(9) $$\;\text{Reject}\;H_{0}^{p}\;\text{if:}\;\exists p^{\prime }\geq p_{i}:{\hat{cFDR}}_{S}\left(p^{\prime },q_{i}\right)\leq \alpha .$$

This enables a rejection region with a single rightmost boundary, as shown in panels D in Figure 1. It closely parallels the B-H (Benjamini and Hochberg, 1995) procedureon a set of p-values. Suppose we have a set *S*_1_ of p-values *p*_1_, *p*_2_, …, *p_n_*, and define

$$BH_{S_{1}}(p)=p\frac{\left|S_{1}\right|}{\max\left(1,\left|\left\{i:p_{i}\leq p,p_{i}\in S_{1}\right\}\right|\right)}.$$

Then the B-H procedure can be written as

(10) $$\;\text{Reject}\;H_{0}^{p}\;\text{if:}\;\exists p^{\prime }\geq p_{i}:BH_{S_{1}}\left(p^{\prime }\right)\leq \alpha .$$

The B-H procedure controls FDR at *α*, and if it is performed with *S*_1_ as the subset of *S* for which *q_i_* ≤ γ (where *γ* is a threshold chosen independently of *S*), the FDR will still be controlled at α if assumption (1) is satisfied (Bourgon et al., 2010; Ignatiadis et al., 2016). The rejection procedure (9) is equivalent to repeatedly performing this ‘thresholded’ B-H at *γ* = *q_i_*, and using that decision rule for point (*P_i_*, *q_d_* (panel c, Figure 1).

When procedure (9) is used, the FDR is no longer controlled at *α*, and indeed can exceed α by an arbitrary proportion. This is most easily seen in the extreme case

(11) $$P,Q|H_{0}^{p}∼U{\left(0,1\right)}^{2}$$

(12) $$P,Q|H_{1}^{p}∼(0,0),$$

in which we can show that the FDR *α_TRUE_* of rejection procedure (9) applied to $\hat{cFDR}$ satisfies

(13) $$\underset{n\to \infty }{\lim}\left(\frac{\alpha_{TRUE}(1-\alpha )}{\alpha \left(1-\alpha_{TRUE}\right)}\right)=\log\left(\frac{1-\alpha \pi_{0}}{1-\pi_{0}}\right)$$

and when applied to ${\hat{cFDR}}^{n}$ satisfies

(14) $$\underset{n\to \infty }{\lim}\left(\frac{FDR}{\alpha }\right)=\frac{1-\log\left(\frac{\alpha }{1-\alpha }\frac{1-\pi_{0}}{\pi_{0}}\right)}{1-\alpha \log\left(\frac{\alpha }{1-\alpha }\frac{1-\pi_{0}}{\pi_{0}}\right)}$$

with the corollary that the actual FDR when using rejection procedure (9) can be an arbitrarily large multiple of *α*. These formulae are proved in Theorem A.3 (AppendixA.2).

Previous work using the cFDR generally interprets it in a Bayesian context, without requiring a bound on FDR or FWER. In Liley and Wallace (2015), we introduced a method to choose an α^*^ such that rejection criterion (9) roughly controlled the FDR at *α*, but that method was overly conservative, generally controlling FDR at a far lower level than needed.

## 3 Map From p-Value Pairs to V-Values

We identify a ‘rejection region’ associated with $\hat{cFDR}$ by adding a ‘test point’ (*p*, *q*) to a set of points *X* and considering the region for which a hypothesis corresponding to (*p*, *q*) is rejected under (9) with *S* = *X* + (*p*, *q*).

The function ${\hat{cFDR}}_{X+(p,q)}(p,q)$ is now defined on the unit square. It is difficult to use, however: when considered as a function of *p* with fixed *q*, it does not monotonically increase with *p*. We thus define

(15) $$\hat{cFDR}t_{X}(p,q)=\underset{p^{\prime }\geq p}{\min}{\hat{cFDR}}_{X+\left(p^{\prime },q\right)}\left(p^{\prime },q\right)$$

and define the ‘L-region’ *L_X_* (α):

(16) $$L_{X}(\alpha )=\left\{(p,q):\hat{cFDR}t_{X}(p,q)\leq \alpha \right\}$$

(17) $$=\left\{(p,q):\exists p^{\prime }\geq p:{\hat{cFDR}}_{X+\left(p^{\prime },q\right)}\left(p^{\prime },q\right)\leq \alpha \right\}$$

and define the ‘L-curve’ as the rightmost boundary of this region. We note that

(18) $$\alpha \leq \beta \Rightarrow L_{X}(\alpha )\subseteq L_{X}(\beta ).$$

We now show the following, and include the brief proof:

### Theorem 3.1

*Assume that $P,Q|H_{0}^{P}$ is a bivariate continuous random variable with support* [0, 1]^2^, *and set μ_0_ as its induced measure. Suppose X* = (*P_i_*, *q_i_*) ∈ [0, 1]^2^, *i* ∈ 1 … *n is a fixed finite set of points. Define L_x_* (α) *as per Equation (16), with $\hat{cFDR}$ defined as per either Equation (6) or Equation (8), and define the ‘v-value’ as a function of p*,*q* ∈ (0, 1)^2^

(19) $$v_{X}(p,q)=\underset{\gamma :(p,q)\in L_{X}(\gamma )}{\inf}\left(\mu_{0}\left[L_{X}(\gamma )\right]\right)\;\left(=\underset{\gamma :(p,q)\in L_{X}(\gamma )}{\min}\left(\underset{L_{X}(\gamma )}{\int }f_{0}(x,y)dxdy\right)\right)$$

*the second definition being valid if* $P,Q|H_{0}^{P}$ *admits a PDF (as in Section 2). Then for* α ∈ (0,1)

(20) $$Pr\left(v_{X}(P,Q)\leq \alpha |H_{0}^{P}\right)\leq \alpha .$$

*Proof.* Since *X* is finite *L_x_* (α) is Lesbegue-measurable, so the integral in(19) is well-defined. Note that since L-regions are closed regions inside contours of a function monotonic in *p*, we could replace ‘inf’ with ‘min’ in definition (19).

Given α ∈ (0,1), let

(21) $$\text{Γ}(\alpha )=\left\{\gamma :\mu_{0}\left[L_{X}(\gamma )\right]\leq \alpha \right\}.$$

Suppose there exists γ(α) ∈ Γ(α) with (*p*, *q*) ∈ *L_X_* (γ(α)). Then from definition (19), we have

(22) $$v_{X}(p,q)\leq \mu_{0}\left[L_{X}(\gamma (\alpha ))\right]\leq \alpha$$

so, using property (18)

$$\begin{array}{ccc} Pr\left(v_{X}(P,Q)\leq \alpha |H_{0}^{P}\right) & \leq & Pr\left[(P,Q)\in \underset{\gamma (\alpha )\in \text{Γ}(\alpha )}{\cup^{\text{​}}}L_{X}(\gamma (\alpha ))|H_{0}^{P}\right]\\ & = & \underset{\gamma (\alpha )\in \text{Γ}(\alpha )}{\sup}\left(\mu_{0}\left[L_{X}(\gamma (\alpha ))\right]\right)\\ & \leq \alpha . \end{array}$$

If there exists *γ* such that α = μ_0_[L(χ)] then (*p*, *q*) ∈ *L_x_* (γ) ⇔ *v_x_* (*p*, *q*) ≤ α, so equality is achieved in (20).

### Remark 3.2

With Γ defined as per (21), and setting *μ*_1_ as the induced measure of $P,Q|H_{1}^{P}$ , the power of the rejection procedure

(23) $$\left\{RejectH_{0}^{P}\;\text{if}\;v_{X}(p,q)\leq \alpha \right\}$$

is

(24) $$Pr\text{(reject}\;H_{0}^{P}|H_{1}^{P}\text{)}=\underset{\gamma (\alpha )\in \text{Γ}(\alpha )}{\sup}(\mu_{1}(L_{X}(\gamma (\alpha ))),$$

and the type-1 error rate is ≤ α.

Theorem 3.1 assumes the probability measure *F_0_* of $P,Q|H_{0}^{P}$ is known. In practice, it must be estimated. This can be readily done given assumption (1), as will be shown in Section 3.2. We can think of the function *v_x_* (*p*, *q*) as a map from the unit square to the unit interval, where the map is defined by the points *X*.

We note that property (20) indicates that the value *v_x_* (*p*, *q*) is interpretable as a p-value against $H_{0}^{p}$ , using $\hat{cFDRt}(p,q)$ as a test statistic (it may even be thought of as a definition of a p-value; Storey et al., 2003). In this sense, it is slightly conservative due to the ‘≤ α’ rather than ‘= α’ in (20). Resultant v-values may thus be used in the B-H procedure to control FDR, or used with a Šidák correction to control FWER. For the remainder of this paper, we will seek to control FDR by the B-H procedure.

In order to use Theorem 3.1 to generate a p-value for a test point (*p*, *q*). we must assume *X* is ‘fixed’. In practice, this means (*p_i_*, *q_i_*) must be independent of values (*p*, *q*); hence, not in *X*.

Given our set of points *S* = (*p_i_*, *q_i_*), this is easily managed: to test (*p_i_*, *q_i_*), we simply leave (*p_i_*, *q_i_*) out of the points used to define the set of L-regions we use on (*p_i_*, *q_i_*) itself. That is, given our set *S* of datapoints as above, we define the ‘leave-one-out’ v-value

(25) $$v\left(p_{i},q_{i}\right)=v_{S-\left(p_{i},q_{i}\right)}\left(p_{i},q_{i}\right).$$

The problem can also be managed by leaving out blocks of points; for a partition of 1 … *n* into blocks 1,2,…, *k*, supposing *i* is in block *b*(*i*), the ‘block-out’ v-value is defined as

(26) $$v\left(p_{i},q_{i}\right)=v_{S-\text{block}b(t)}\left(p_{l},q_{i}\right).$$

If observations (*p_i_*, *q_i_*) are not independent but have a block-diagonal correlation structure, then this procedure is necessary in order to ensure property (20) holds for (*p*, *q*) = (*p_i_*, *q_i_*): since each observation (*p_i_*, *q_i_*) carries information about the other p-value pairs it is correlated with, removing it will not remove the influence of point (*p_i_*, *q_i_*) on the map. In this case, blocks should be chosen so that p-value pairs are independent between blocks, but possibly dependentwithin blocks. Such structure arises often in -omics experiments; in genetics, independence of allele counts may be assumed between chromosomes, but not generally within.

For comparison, we also define the ‘naive’ v-value

(27) $$v\left(p_{i},q_{l}\right)=v_{S}\left(p_{l},q_{l}\right),$$

where *p_i_*, *q_i_* is in *S*.

In the subsequent section, we note several asymptotic properties of L-regions and v-values. We note that consistency of L-region estimation is not necessary for type-1 error rate control: there is no requirement in Theorem 3.1 for the values in *X* to have the same distribution as *P*,*Q*. L-regions are also identical under monotonic transformations of the function $\hat{cFDR}(p,q)$ , so the method is unaffected by the approximation $Pr\left(H_{0}^{P}\right)\approx 1$ in Equation (7).

### 3.1 Asymptotic properties of L-regions and v-values

We describe several asymptotic properties of L-regions and v-values. Estimator (7)is not generally consistent, so we focus our attention on properties of regions defined using $\hat{cFDR}$ rather than ${\hat{cFDR}}^{n}$ . We divide out the quantity estimated in (7), write *F*(*q*) = *Pr*(*Q* ≤ *q*) and define

(28) $$C(p,q)=\frac{cFDR(p,q)}{Pr\left(H_{0}^{p}|Q\leq q\right)}=p\frac{F(q)}{F(p,q)},$$

which we generally assumed to be differentiable on the unit square. We recall definition (15) and note the following (proved in Appendix A.3):

#### Theorem 3.3

*Let R be the region of the unit square for which F*(*p*, *q*) ≥ γ > 0 and *F*(*q*) > 0. *ThenonR, $\hat{cFDR}(p,q)$ converges uniformly to C*(*p*, *q*), *and if 𝜕C*(*p*, *q*)/*𝜕p* ≥ 0, *then so does* $\hat{cFDRt}(p,q)$ .

This theoremindicates that the empirical L-curve converges to a contour of *C*(*p*, *q*), where the contour exists. Although the region *R* does not cover the entire unit square, in practice it usually has Lesbegue measure 0: if $Pr\left(H_{0}^{Q},H_{0}^{P}\right)>0$ and $P,Q|H_{0}^{Q},H_{0}^{P}∼U{\left(0,1\right)}^{2}$ then *F*(*p*, *q*) is bounded away from 0 on (0,1)^2^. We note that *C*(*p*, *q*) is meaningful only when *F*(*p*, *q*) > 0.

Because of the uniform convergence, this can be translated into a statement about v-values. Given an L-region *L*(α), we define the M-region as the ‘expected’ L-region:

(29) M(α)={(p,q):C(p,q)≤α}

and the ‘error’ on the v-value *v* = ∫_L(α)_ f_0_(*p*, *q*)*dpdq* as

(30) $$|\text{Δ}v|=\left|\int_{L(\alpha )}f_{0}(p,q)dpdq-\int_{M(\alpha )}f_{0}(p,q)dpdq\right|.$$

Now we have

#### Theorem 3.4

*Define R as in Theorem 3.3, and further assume that* $f_{0}(p,q)=f\left(P=p,Q=q|H_{0}^{P}\right)$ *is known and on R we have 𝜕C*(*p*, *q*)/*𝜕p* ≥ γ_2_ > 0. *Write R^c^* = [0,1]^2^ \ *R. Then the maximum error on any v-value is*

(31) $$\int_{R^{c}}f_{0}(p,q)dpdq+O\left(\frac{1}{\sqrt{n}}\right)$$

thus, as *n* → ∞, v-values based on $\hat{cFDR}$ converge at a rate *O*(*n* ^-1/2^) to those that would be obtained using *C*(*p_i_*, *q_i_*), plus a fixed error. We also note that under the conditions in both theorems, if *R* has negligible Lesbegue measure, and there exists *γ* such that

$$\int_{M(\gamma )}f_{0}(p,q)dpdq=1$$

then as *n* → ∞ the power of rejection procedure (23) satisfies

(32) $$Pr\left(\;\text{reject}\;H_{0}^{P}|H_{1}^{P}\right)\to \mu_{1}[M(\gamma )],$$

where *μ*_1_ is defined as in Equation (23). Finally, we note that neither consistency nor unbiasedness of the $\hat{cFDR}$ estimator is necessary for the p-value property in Theorem 3.1 to hold. Proofs of Theorems 3.3 and 3.4 are given in Appendix A.3.

### 3.2 Estimation of $P,Q|H_{0}^{p}$

Recalling Equation (3), wemaywrite $f_{0}(p,q)=f_{0}^{q}(q)$ . To estimate $f_{0}^{q}$ , we assume that $\left(Q|H_{0}^{p}\right)$ and approximate the latter with a mixture-Gaussian distribution

(33) $$-{\text{Φ}}^{-1}\left(\frac{Q}{2}\right)|P>\frac{1}{2}∼\{\begin{array}{ll} \left|N(0,1)\right| & \text{prob}\;=\pi_{0}\\ \left|N(0,\sigma_{0}^{2})\right| & \text{prob}\;=1-\pi_{0}, \end{array}$$

where *N*(μ, σ) is the normal distribution with mean *μ* and variance *σ* ^2^. Estimates $\hat{\pi_{0}}$ , $\hat{\sigma_{0}}$ of *π*_0_ and σ_0_ can be readily made using an expectation-maximisation algorithm (Dempster et al., 1977), using the values *q_i_* for which the corresponding *p_i_* is ≥ 1/2. We then estimate the density *f*_0_(*p*, *q*) of $P,Q|H_{0}^{P}$ as

(34) $$\hat{f_{0}}(p,q)=1f_{0}^{q}(q)=\hat{\pi_{0}}+\left(1-\hat{\pi_{0}}\right)\frac{\phi \left({\text{Φ}}^{-1}(q/2),\sigma =\hat{\sigma_{0}}\right)}{\phi \left({\text{Φ}}^{-1}(q/2),\sigma =1\right)},$$

where *φ* is the normal density function with SD σ. If *P*, *Q* have a known dependence under $H_{0}^{p}$ , an alternative distribution can be used for computing *v*(*L*) (see Supporting Information Section 1.2). The PDF $f_{0}^{q}$ could be estimated in other ways; for example, a kernel density estimate (Sheather and Jones, 1991).

Type-1 error control is maintained under a relaxation of the assumption that $P|H_{0}^{P}∼U(0,1)$ if the distribution of *P* dominates *U*(0,1); that is, if $Pr\left(P\leq p|H_{0}^{P}\right)\leq p$ for all *p*, since we will overestimate *f*_0_ inside L-regions in this case and hence v-values will be conservative.

### 3.3 Correlation between v-values

Decision rules based on multiple p-values generally require adjustment if p-values are dependent (e.g. Benjamini and Hochberg, 1995). If v-values are obtained by the leave-one-out procedure (25) they are slightly pairwise dependent. The dependence is small; if *X’* = *X* − (*p_i_*, *q_i_*) − (*p_j_*, *q_j_*) the values *v_X’_* (*p_i_*, *q_i_*), *v_X’_* (*p_j_*, *q_j_*) are independent, so the pairwise dependence between v-values corresponding to (*p_i_*, *q_i_*), (*p_j_*/*q_i_*) only arises from the differences *v_X’+(*p_j_*, *q_j_*)_* (*p_i_*, *q_i_*) − *v_X’_* (*p_i_*, *q_i_*), *v_X’+(*p_i_*, *q_t_*)_* (*p_j_*, *q_j_*) − *v_X’_* (*p_j_*, *q_j_*)·; that is, the effect of a single point ((*p_j_*, *q_j_*), (*p_i_*, *q_i_*, respectively) on the map *v_X’_* defined by |*X’*| = |*X*| − 2 points. Indeed, we show that the expected change to v-values on adding a single new point is small:

#### Theorem 3.5

*Suppose we add a point* (p^*^, ^q*^) *to a set of n points* (*p_i_*, *q_i_*), *considered as realisations of P, Q and conditions are satisfied for convergence of v-values as above. Let Δv*(*L*(α)) *be the shift in a v-value corresponding to an L-curve L*(α) *after adding* (p^*^, q^*^). Then

(35) $$E_{\alpha ∼U(0,1)}(|\text{Δ}v(L(\alpha ))|)=O\left(\frac{1}{n^{2}}\right).$$

The proof is given in Appendix A.4. When v-values are defined using block-out as in (26), v-values are independent within-block but dependent between blocks. The B-H procedure is also sensitive to higher order (non-pairwise) dependence between v-values, but we show by simulation in Section 5 residual dependence does not generally lead to failure of FDR control, even when we increase dependence by enforcing correlation between observations *p_i_*, *p_j_* and between *q_i_*, *q_j_*.

### 3.4 Algorithm

We can now present our final algorithm.

We can interpret *v_i_* as ‘the probability that a randomly chosen (*p*, *q*) pair has a more extreme $\hat{cFDR}$ value than $\hat{cFDR}\left(p_{i},q_{i}\right)$ ; that is, as a p-value. This allows straightforward FWER or FDR control, especially as v-values are almost independent. The v-values order hypotheses such that a rejection rule {reject $H_{0}^{P}r(i)\;\text{if}\;v_{i}\leq \alpha$ } has near-optimal power, in

Algorithm 1 **Controlling type-1 error rate in cFDR**

**Input**: ‘principal’ p-values *p*_1__,*p*_2_, …*p_n_*; ‘conditional’ p-values *q*_1_,*q*_2_, …*q_n_*; optionally fold assignment *b* : 1… *n* → 1… *k* such that (*P_i_*,*q_i_*) ⫫ (*p_j_*,*q_j_*)\*b*(*i*) = *b*(*j*)

**Output**: v-values *v*_1_,*v*_2_ …*v_n_*

1: Identify the set {*q_i_* : *P_i_* > 1/2} and make estimates $\hat{\pi_{0}}$ , $\hat{\sigma_{0}}$ of π_0_, σ_0_ as per (33)

2: Set ${\hat{f}}_{0}(p,q)$ as per Equation (34)

3: **for** *i* ∈1 …*n* **do**

4: Set *S’* = {(*P_j_*,*q_j_*) : *j* ≠ *i*} (leave-one-out) or *S*’ = {(*P_j_*,*q_j_*) : *b*(*j*) ≠ *b*(*i*)} (block-out)

5: Find *c_i_* = min{*c* : (*p_i_*,*q_i_*) ∈ *L_s>_* (*c*)}

6: Set $v_{i}=\int_{L_{s^{\prime }}\left(c_{i}\right)}{\hat{f}}_{0}(p,q)dpdq$

7: Return (*v*_1_,*v*_2_, …,*v_i_*)

terms of corresponding to near-optimal forms for rejection regions. Much ofour methodologycanalso be used ifvalues *q_t_* are not p-values for some second trait, as long as they fall in (0,1). However, approximation (33) may be inappropriate if this is not the case.

## 4 Relation to Other Methods

A wide range of approaches have been proposed for the problem of high-dimensional association testing using an informative covariate. Given the correspondingly wide variation in problems of this type, the optimal methodis likely to depend on circumstance. In general, we will take *P*,*p*, $H_{0}^{p}$ to refer to p-values and hypotheses for the trait of primary interest, and *Q*,*q* to refer to the covariate.

### 4.1 Determination of rejection region form

The simplest approach to covariate-based testing is ‘independent filtering’ (Bourgon et al., 2010) in which attention is restricted to the set {(*p_i_*,*q_j_*) : *q_i_* ≥ *q*_0_}, with the B-H procedure then applied to the corresponding subset of values of *p_i_*. This procedure is equivalent to rejection regions which are a series of rectangles with upper border at *q* = *q*_0_. Independent filtering is clearly non-optimal, but is well-suited to some problem types (Bourgonetal., 2010).

As discussed above, a range of approaches aim to approximate the optimal rejection regions based on *f*_0_/*f*. In Du et al. (2014) and Lei and Fithian (2018), parameterisation leads to rejection regions constricted to a particular parametric class; in Du et al. (2014) that of oracle rejection regions under mixture-Gaussian forms of *f*_0_ and *f*. In Alishahi et al. (2016) and Zablocki et al. (2014), boundaries of rejection regions are necessarily smooth at a scale corresponding to the smoothing kernel width, but can take otherwise arbitrary forms. An alternative approach is to ‘bin’ covariates (Ferkingstad et al., 2008; Ignatiadis et al., 2016) which leads to L-curves which are step functions with steps spaced according to the resolution of the bins.

An approach in Li and Barber (2016) estimates $Pr\left(H_{0}^{p}|Q=q\right)$ for each *q* to modulate a B-H type test for each observation. The entire effect of the covariate in this method is encompassed through the value of $Pr\left(H_{0}^{p}|Q=q\right)$ , which necessarily relies on point-estimates of the PDF $f\left(Q=q|H_{0}^{p}\right)$ , and hence the method is dependent on the accuracy of this estimate.

Another common approach to covariate modulation is the weighted B-H procedure (Benjamini et al., 2006), in which each p-value *p_i_* is reweighted to a value *P_i_*/*W_i_* (where ∑ *w_i_* = 1) and the standard B-H procedure is then applied to the values *P_i_*/*W_i_*. Our method can be interpreted in these terms, setting *W_i_* = *v_i_*/*p_i_*, but this is rather unnatural; there is no clear way to interpret what the ratio *v_i_*/*p_i_* means, and this approach does not make use of the ‘p-value property’ in Equation (20).

The use of ECDFs to generate rejection regions has the advantage of making use of the global distribution of *P*, *Q*, while spline- and kernel-density based estimates can generally only use local observations. The cFDR-based method has the obvious disadvantage of not converging to the optimum rejection region, and it can be less powerful than parametric approaches if parametric assumptions hold. However, using CDFs rather than PDFs allows faster convergence of rejection regions with *n*, and this favours the cFDR approach if *n* is small, particularly if CDF- and PDF- based regions are similar and PDFs are difficult to model well.

Under certain circumstances, contours of CDF- and PDF-based methods are similar. A precise statement, proof and demonstration is given in Appendix A.5.

### 4.2 Censoring of points

In general terms, the process determining a decision rule to be used on observation (*p_i_*,*q_i_*) cannot easily make use of the datapoint (*p_i_*,*q_i_*) itself, since the use of the point biases the choice of decision rule in some way. Approaches by Du et al. (2014) and Alishahi et al. (2016) censor the points used in the decision rule to those already rejected in a stepwise approach, and a method in Lei and Fithian (2018) masks the information available for the decision rule by effectively adding the point (1 – *P_i_*,*q_i_*) to the dataset.

Since cFDR uses the entire dataset to estimate ECDFs, complex censoring can require that the cFDR estimator be changed in a non-trivial way. In particular, there is no obvious way to apply the methods proposed by Alishahi et al.(2016) or Lei and Fithian (2018). We propose avoiding the problem by leaving out the point (*p_i_*,*q_i_*) directly (Equations (25) and (26)), at the cost of residual correlation in resultant v-values. While crude, this corresponds to a near-minimum censorship of points, and the resultant correlation tends to be small enough to ignore (see Appendix A.4).

### 4.3 Asymmetry and management of extreme outliers

An important property of the cFDR-based method is asymmetry, in that $H_{0}^{p}$ cannot generally be rejected based on a low *q_i_* alone (this can be seen by noting that $\hat{cFDR}\left(p_{i},q_{i}\right)\geq p_{i}$ , and *p_i_* can only exceed ${\hat{cFDR}}^{n}$ in rare circumstances). Parametric approaches such as those in Du et al. (2014) and Lei and Fithian (2018) are not generally robust to this; for example, in Du et al. (2014), an extremely low *q_i_* could lead to rejecting $H_{0}^{P}$ even if the corresponding *p_i_* were close to 1 and *P*, *Q* were independent (given that the degree of dependence is estimated). This property of the cFDR is very important when *p_i_* and *q_i_* are derived from genome-wide association studies (GWAS) on different diseases; it is entirely possible and even expected that a very strong association in the conditional trait is not an association with the principal trait. This property also differentiates our approach from meta-analysis of two sets of p-values.

### 4.4 Relation to original FDR-controlling method for cFDR

In a paper in 2015 (Liley and Wallace, 2015), we identified the problem of failure of FDR control at α when using a rejection rule $\hat{cFDR}\leq \alpha$ and proposed a rough solution. We proposed identifying L-curves and estimating *f*_0_ as above, and for each L-region *L_s_* (α^*^), identifying a rectangle *R*(α^*^) contained within it with vertices (0,0), (0, *q_r_*), (*p_r_*, 0), (*p_r_*,*q_r_*). Since *R*(α^*^) ⊆ *L_s_* (α^*^), we have

(36) $$Pr((P,Q)\in L_{S}(\alpha^{*}))\geq Pr((P,Q)\in R(\alpha^{*}))$$

and $\left(\hat{p_{r},q_{r}}\right)\leq \alpha^{*}$ , so the FDR associated with rejecting any (*p*, *q*) pairs falling in *L_s_* (α^*^) was approximately

(37) $$\begin{array}{ccc} E\left(\frac{\left|\left\{i:p_{i},q_{i}\in L_{S}\left(\alpha^{*}\right),H_{0}^{p}\right\}\right|}{\min\left(\left|\left\{i:p_{i},q_{i}\in L_{S}\left(\alpha^{*}\right)\right\}\right|,1\right)}\right) & \approx & \frac{Pr\left((P,Q)\in L_{S}\left(\alpha^{*}\right)|H_{0}^{p}\right)}{Pr\left((P,Q)\in L_{S}\left(\alpha^{*}\right)\right)}\\ & \leq & \frac{Pr\left((P,Q)\in L_{S}\left(\alpha^{*}\right)|H_{0}^{p}\right)}{Pr\left((P,Q)\in R\left(\alpha^{*}\right)|H_{0}^{p}\right)}\frac{Pr\left((P,Q)\in R\left(\alpha^{*}\right)|H_{0}^{p}\right)}{Pr\left((P,Q)\in R\left(\alpha^{*}\right)\right)}\\ & \approx & \frac{Pr\left((P,Q)\in L_{S}\left(\alpha^{*}\right)|H_{0}^{p}\right)}{Pr\left((P,Q)\in R\left(\alpha^{*}\right)|H_{0}^{p}\right)}{\hat{cFDR}}_{S}\left(p_{r},q_{r}\right) \end{array}$$

(38) $$\leq \frac{\int_{L_{S}\left(\alpha^{*}\right)}f_{0}dpdq}{\int_{R\left(\alpha^{*}\right)}f_{0}dpdq}\alpha^{*}.$$

To approximately control FDR at α, our procedure found α^*^ so that expression (38) was ≤ α and rejection $H_{0}^{p}$ whenever (*P_i_*,*q_i_*)∈*L_s_* (α^*^).

As well as being approximate, this procedure was conservative due to inequality (36). Our new method avoids this conservative assumption and is on firmer theoretical ground. Furthermore, our old method precluded use of ${\hat{cFDR}}^{n}$ given approximation (37). We show by simulation below that this results in substantial improvement in power in our new method.

## 5 Assessment of Performance

In this section, we address five main points. Firstly, we demonstrate that our new method controls type-1 error rate (FDR) appropriately and that the censoring approach of (25) and (26) is necessary. Secondly, we demonstrate that power is substantially improved relative to our previous method for fixed level of FDR control, and that use of ${\hat{cFDR}}^{n}$ over $\hat{cFDR}$ improves power further. We then demonstrate that in settings where parametric assumptions are not satisfied, rejection regions based on ${\hat{cFDR}}^{n}$ can correspond to a more powerful procedure than rejection regions based on alternative CDF or PDF estimators. We examine the effect of correlation between observations *P_i_*,*q_i_* on our main FDR-controlling methods and demonstrate that the disadvantage of using our leave-one-out method (Equation (25)) instead of the leave-out-block method (Equation (26)) out method in the presence of correlationis loss of power rather than loss of FDR control.Finally, we assess the degree of shared association between *P* and *Q* which is necessary for our method to give an advantage over p-values alone.

In each simulation, we sampled a set of values *S* = (*P_i_*,*q_i_*), *i*∈1… *n*. The sampling schema we used itself depended on a series of underlying parameters, which were themselves sampled from a joint distribution specified in Table 1, ora conditional distribution of it. We also separately considered several fixed values of parameters.

We first chose a fixed total number of hypotheses *n*, then split these into four classes of fixed size:*c*_1_ of size $n_{1}^{pq}$ associated in both *P* and *Q*, *C*_2_ of size $n_{1}^{p}$ associated only with *P*, *C*_3_ of size $n_{1}^{q}$ associated only with *Q* and *C*_4_ associated with neither *P* nor *Q*. Within each class, samples (*P_i_*,*q_i_*) were identically distributed.

For *i* ∈ *C*_1_, *C*_2_, we sampled *p_i_* (determined by *d*, *s_p_*) by first simulating Z scores:

$$\begin{array}{l} d=1:-{\text{Φ}}^{-1}\left(\frac{p_{i}}{2}\right)\frac{1}{s_{p}}∼N(0,1)\\ d=2:-{\text{Φ}}^{-1}\left(\frac{p_{i}}{2}\right)\frac{1}{s_{p}}∼t(\text{df}=3,\text{ncp}=0)\\ d=3:-{\text{Φ}}^{-1}\left(\frac{p_{i}}{2}\right)\frac{1}{s_{p}}∼\;\text{Cauchy}\;(\;\text{location}\;=0,\;\text{scale}\;=1), \end{array}$$

where $-{\text{Φ}}^{-1}\left(\frac{p_{i}}{2}\right)$ can be considered a Z-score corresponding to *p_i_*, and *s_p_* a scaling factor for the distribution. We set the distribution of *q_i_* ∈ *C*_1_,*C*_3_ similarly, with *s_q_* in place of *s_p_*. The values *p_i_*, *q_i_* for *i* ∈ *C*_4_ were sampled from *U*(0,1).

Although effect sizes are often assumed to follow normal distributions, real data are often noisier, with longer tails, and recent work suggests non-normal distributions may be a better fit in the case of GWAS data (Walters et al., 2019). We chose the alternative distributions (normal, t (3df), and Cauchy) to span behaviours from ‘well-behaved’ (normal) to ‘long-tailed’ (t) to ‘chaotic’ (Cauchy) to survey a wider range of possibilities.

Samples *P_i_*,*P_j_* and *q_i_*, *q_j_* were generally independent (unless otherwise specified), but we also sampled under two patterns of dependence. Firstly, we simulated a ‘block’ correlation structure in which we divided samples into three blocks, and within each block sampled z-scores *z_pi_*, *z_pj_*, *z_qi_*, *z_qj_* corresponding to *P_i_*, *P_j_* and *q_i_*, *q_j_* such that cor(*z_pi_*, *z_pj_*) = cor(*z_qi_*, *z_qj_*) = *p* if *i*, *j* were in the same block and class, and cor(*z_qi_*, *z_qj_*) = cor(*z_qi_*, *z_qj_*) = 0 otherwise. Secondly, we simulated an equicorrelation structure in which cor(*z_qi_*, *z_qj_*) = cor(*z_qi_*, *z_qj_*) = *p* whenever *i* and *j* were in the same class. When *d* ∈ 2,3, we used the off-diagonal elements of the normalised dependence matrix in the multivariate T distribution in place of correlation.

When relevant, we also sampled parameters from the distribution specified in Table 1 conditional on $n_{1}^{p}+n_{1}^{pq}=0$ ; that is, no associations with *P*. We plotted results from these simulations separately to those with parameters drawn from the unconditional distribution.

Given a rejection procedure, we defined

$$\begin{array}{l} FDP=\{\begin{array}{ll} 0 & \text{if}\;\text{no}\;\text{rejections}\\ \frac{\;\text{number}\;\text{of}\;\text{falsely}\;\text{rejected}\;\text{null}\;\text{hypotheses}\;}{\;\text{total}\;\text{number}\;\text{of}\;\text{rejections}\;} & \text{if}\;\geq 1\;\text{rejection} \end{array}\\ TDP=\{\begin{array}{ll} 0 & \text{if}\;\text{no}\;\text{rejections}\\ \frac{\;\text{number}\;\text{of}\;\text{correctly}\;\text{rejected}\;\text{null}\;\text{hypotheses}\;}{\;\text{true}\;\text{number}\;\text{of}\;\text{associations}\;} & \text{if}\;\geq 1\;\text{rejection}. \end{array} \end{array}$$

We analysed type 1 error in terms of the estimated FDR, $FDR=E(FDP)\approx \bar{FDP}$ and power in terms of the corresponding true-discovery rate $TDR=E(TDP)\approx \bar{TDP}$ . We compared *FDP* and *TDP* between samples by estimating them via a Gaussian-weighted moving average across the independent variable (usually $n_{1}^{p}+n_{1}^{pq}$ ). We show 95% pointwise confidence envelopes derived as per Gatz and Smith (1995), except in cases where such envelopes obstruct viewability of the plot. In these cases, we state that values *TDR(A)* for one method *A* ‘exceed’ paired values *TDR (B)* of another method *B* if in at least six of eight equal subdivisions of the *x*-axis range the following three conditions hold: *TDR*(*A*) > *TDR*(*B*) more than *TDR*(*B*) > *TDR*(*A*), the mean *TDR*(*A*) - *TDR*(*B*) is positive and a Wilcoxon test of ranks on *TDR*(*A*) - *TDR*(*B*) rejects the null hypothesis of a symmetric distribution around 0 with *p* < 5× 10^–3^ (or the equivalent with *FDR* in place of *TDR*). Each line on each plot corresponds to > 5000 simulation runs. All raw simulation results and analytic code are publically available at https://github.com/jamesliley/cfdr_pipeline.

### 5.1 New FDR-controlling procedure leads to greater power than previous method, and adjustment improves power further

We first compared FDR control amongst five methods, aiming to control the FDR at either α = 0.1 or α = 0.01:

1. the B-H method applied to the values *p_i_*, labelled ‘P-val’
2. the B-H method applied to ‘naive’ v-values ν(*P_i_*,*q_j_*) = *v_s_* (*P_i_*,*q_i_*) as per Equation (27) for reference, labelled ‘Naive’
3. the B-H method applied to ‘leave-one-out’ v-values ν(*P_i_*,*q_j_*) = *v*_s-(*P_i_*,*q_i_*)_(*p_i_*,*q_i_*) as per Equation (25), labelled ‘LOO’
4. the B-H method applied to block-out v-values (after randomly separating observations into three equally sized subdivisions, so (*P_i_*,*q_i_*) is in subdivision *b*(*i*), defining v-values *v_s-b(i)_* (*P_i_*,*q_i_*)) as per Equation (26), labelled ‘LOB’
5. our previous method for FDR control applied to (*P_i_*,*q_i_*), labelled ‘Orig.’

We sampled simulation parameters according to Table 1 or the corresponding conditional distribution of Table 1 with $n_{1}^{p}+n_{1}^{pq}=0$ .

**Figure 2:**
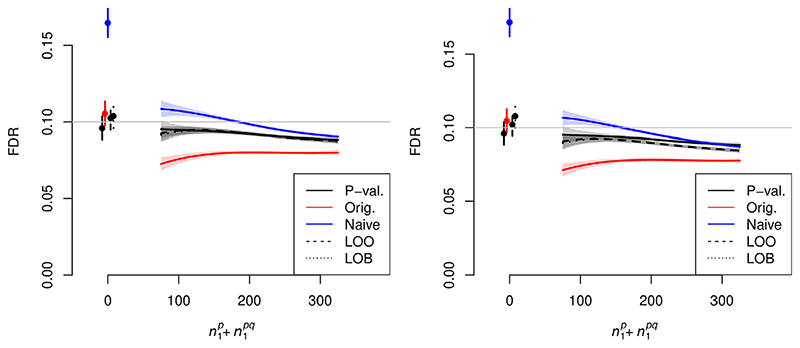
FDR control of various methods against $n_{1}^{p}+n_{1}^{pq}$ , the total number of variables associated with *P* (the primary study under consideration). The horizontal line shows α = 0.1, the desired FDR control level. Simulations in the left panel integrate L-regions over the true distribution *f_0_*; simulations in the right panel integrate over the estimated distribution as per Equation (33). Shaded regions indicate 95% confidence envelopes. Curves show moving weighted averages using a Gaussian kernel with SD 15% of the *x* axis range. Lines on the left indicate FDR control with $n_{1}^{p}+n_{1}^{pq}=0$ corresponding plot with α = 0.01 is shown in Supplementary Figure 7

Expected *FDP* was consistent with the FDR control level when using leave-one-out v-values or ‘block-out v-values (rejection procedures 3,4). When using the ‘naive’ v-values *v_s_* (*P_i_*,*q_i_*) (rejection procedure 2), FDR was not controlled at the requisite level. The FDR using methods 3 and 4 exceeded the FDR of our original method (rejection procedure 5), indicating that our original method was conservative. FDR control was maintained when using the approximation of *f_0_* in Equation (33). Results are shown in Figure 2.

**Figure 3:**
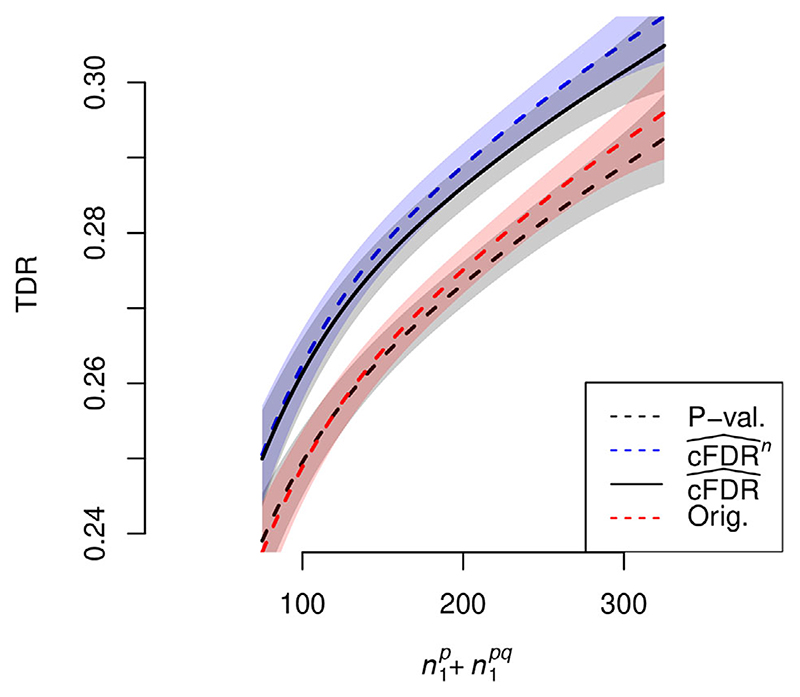
TDR of various methods against $n_{1}^{p}+n_{1}^{pq}$ , the total number of variables associated with *P* (the primary study under consideration), at FDR control level α = 0.1. Shaded areas show 95% pointwise confidence envelopes. A corresponding plot with α = 0.01 is shown in Supplementary Figure 8. Curves show moving weighted averages using a Gaussian kernel with SD 15% of the *x* axis range

Having established the validity of rejection methods 3 and 4, we compared the power of ‘adjusted’ cFDR ( $\left({\hat{cFDR}}^{n}\right)$ ) and non-adjusted cFDR ( $\left(\hat{cFDR}\right)$ ) using the leave-one-out v-value (rejection procedure 3) and the power of our previous method, rejection procedure 5, applied to $\hat{cFDR}$ (labelled ‘Orig’). The TDR of ${\hat{cFDR}}^{n}$ exceeded the power of $\hat{cFDR}$ which in turn exceeded the power of our previous rejection procedure on $\hat{cFDR}$ (Figure 3).

We report FDR and TDR for p-values, ${\hat{cFDR}}^{n}$ and oracle cfdr for a range of fixed simulation parameters in Table 2

### 5.2 PDF-based estimation leads to a less powerful procedure than CDF-based estimation

As *n* → ∞, consistent estimators of $Pr\left(H_{0}^{p}|P=p,Q=q\right)$ will converge to optimal rejection regions while estimators of $Pr\left(H_{0}^{p}|P\leq p,Q\leq q\right)$ will not, and hence the former will ultimately be more powerful. However, we found that under the distribution of simulation parameters in Table 1, the ECDF-based estimator ${\hat{cFDR}}^{n}$ is considerably more powerful than two PDF-based estimators of $Pr\left(H_{0}^{p}|P=p,Q=q\right)$ .

**Figure 4:**
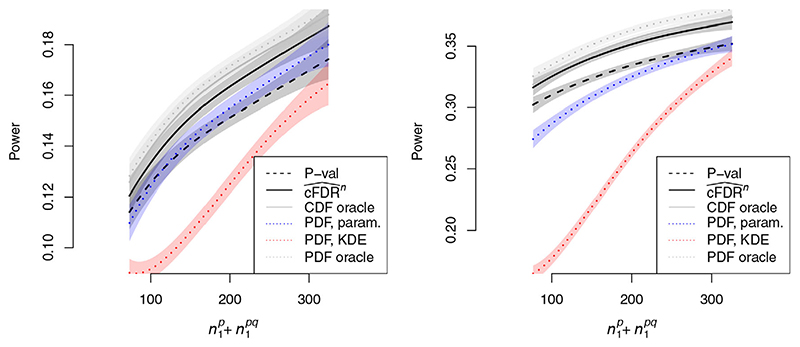
TDR of PDF-based methods against $n_{1}^{p}+n_{1}^{pq}$ , the total number of variables associated with *P* (the primary study under consideration), controlling FDR at α. = 0.1. In the left panel, parametric assumptions were satisfied (i.e. *d* = 1 in Table 1) and in the right panel, they are not (*d* = 2,3). Shaded regions show pointwise 95% confidence intervals. A corresponding plot with α. = 0.01 is shown in Supplementary Figure 8. Curves show moving weighted averages using a Gaussian kernel with SD 15% ofthe *x* axis range

Results are shown in Figure 4. We considered parametric (labelled ‘PDF param’) and KDE-based (labelled ‘PDF KDE’) estimators of $Pr\left(H_{0}^{p}|P=p,Q=q\right)$ . The parametric model was based on a four-Gaussian model detailed in Supporting Information, Section 1.4. We separated cases in which parametric assumptions were satisfied (i.e. *d* = 1 in Table 1) and in which they were not (*d* = 2,3). The TDR of ${\hat{cFDR}}^{n}$ exceeded the TDR of both estimators of $Pr\left(H_{0}^{P}|P=p,Q=q\right)$ . The performance of an oracle CDF procedure (using exact contours of *F*_0_/*F* as rejection regions, labelled ‘CDF oracle’) and an oracle PDF procedure (using exact contours of *f_0_*/*f* as rejection regions, labelled ‘PDF oracle’) are shown for comparison.

#### 5.2.1 Parametric- and KDE-based cFDR estimators are less powerful than the ECDF-based estimator

We also examined PDF- and KDE-based estimates of the cFDR rather than the cfdr. Details ofthe alternative estimators are given in Supporting Information Section 1.4.

**Figure 5:**
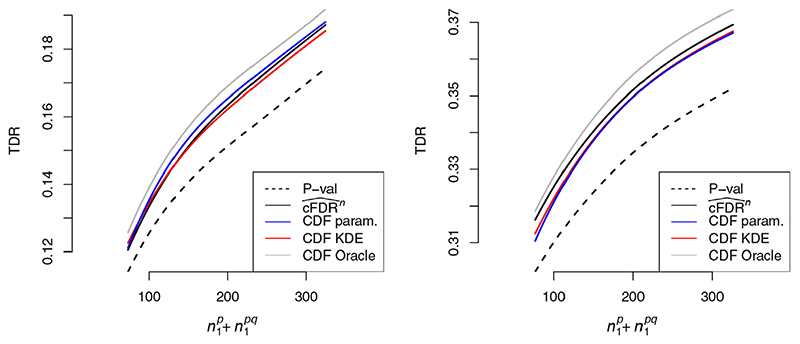
TDR of various methods against $n_{1}^{p}+n_{1}^{pq}$ , the total number of variables associated with *P* (the primary study under consideration), restricting to simulations in which parametric assumptions were satisfied (left panel) or were not satisfied (right panel), at FDR control level α. = 0.1. A corresponding plot with α. = 0.01 is shown in Supplementary Figure 9. Confidence intervals are omitted for visual clarity Curves show moving weighted averages using a Gaussian kernel with SD 15% of the *x* axis range

When parametric assumptions were satisfied (Figure 5, left panel), performance of the ECDF-based ${\hat{cFDR}}^{n}$ , parametric (labelled ‘CDF param’) and KDE-based (labelled ‘CDF KDE’) cFDR estimators was equivocal. When parametric assumptions were not satisfied (*d* = 2,3 as per Table 1; Figure 5, right panel), the TDR of the ECDF estimator exceeded the TDR of the parametric and KDE estimators. The performance of an oracle CDF procedure (using exact contours of *F_0_*/*F* as rejection regions) is shown for comparison.

### 5.3 Correlated samples lead to lower TDR but FDR control is maintained

When *P_i_*,*q_i_* had either block correlation or equicorrelation, we found that FDR control was maintained when using leave-one-out v-values (Equation (25)) and when using leave-out-block v-values (Equation (26)). Under equicorrelation, the TDR of leave-out-block exceeded the TDR of leave-one-out v-values. Under block correlation, although TDR of leave-out- block did not formally exceed the TDR of leave-one-out, apaired Wilcoxon rank-sum test on TDR values rejected the null hypothesis of a symmetric distribution around 0 with *p* < 1× 10^–6^ in favour of leave-out-block.

**Figure 6:**
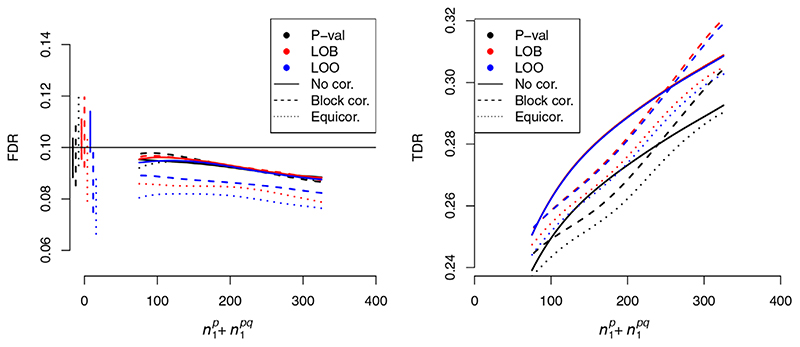
FDR (left) and TDR (right) of FDR-controlling methods leave-out-block (Equation (26)) and leave-one-out (Equation (25)) applied to ${\hat{cFDR}}^{n}$ , and the B-H procedure applied to p-values, under several models of correlation between observations (*p* = 0.01). Confidence envelopes are omitted for visual clarity. Vertical lines show FDR with 95% confidence intervals at $n_{1}^{p}+n_{1}^{pq}=0$ (the p-value appears not to control FDR under equicorrelation, but it is well-known to do so theoretically). A corresponding figure with *p* = 0.1 is shown in Supplementary Figure 11. Curves show moving weighted averages using a Gaussian kernel with SD 15% ofthe *x* axis range

Maintenance of FDR control is expected, as the B-H procedure controls FDR more conservatively when p-values are positively correlated than when independent. Figure 6 shows FDR and TDR controlling at α = 0.1 in the case *p* = 0.01, including performance of p-values *P_i_* under the B-H procedure for comparison. The case *p* = 0.1 is similar and is shown in Supplementary Figure 11.

### 5.4 TDR of ${\hat{cFDR}}^{n}$ becomes higher than TDR of p-value alone when ≈ 20% of hypotheses are shared

**Figure 7:**
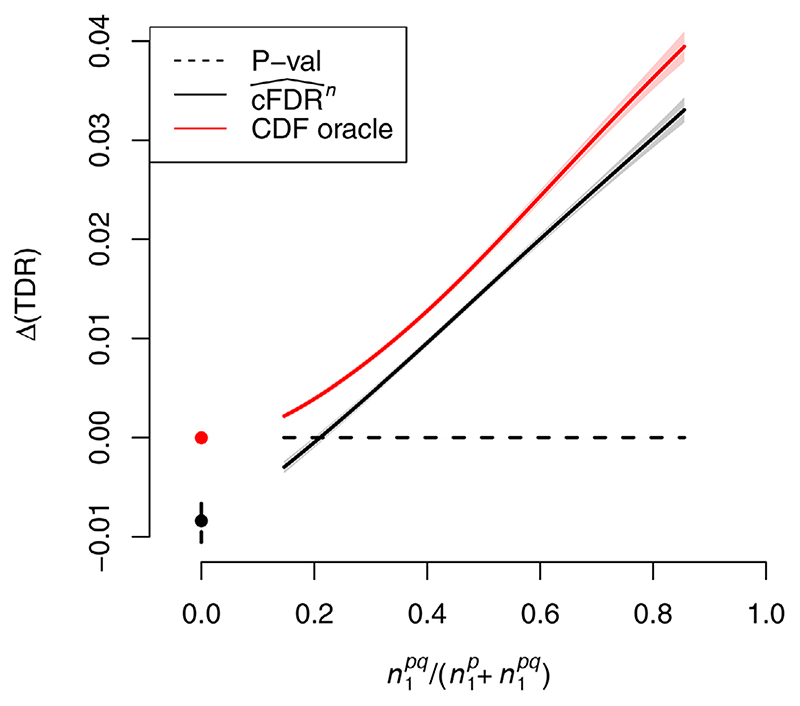
Difference in TDR between ${\hat{cFDR}}^{n}$ (assessed by leave-one-out v-values) and p-values, controlling FDR at α. = 0.1, against $n_{1}^{pq}/\left(n_{1}^{pq}+n_{1}^{p}\right)$ ) (proportion of non-null hypotheses for *P* which are shared with Q). The performance of the oracle CDF method is shown for comparison. Shaded areas show pointwise 95% confidence intervals. Points and lines at the leftmost edge show TDR values and 95% confidence intervals when $n_{1}^{pq}/\left(n_{1}^{pq}+n_{1}^{p}\right)=0.$ corresponding figure with α = 0.01 is shown in Supplementary Figure 12. Curves show moving weighted averages using a Gaussian kernel with SD 15% ofthe *x* axis range

Finally, we assessed the proportion of non-null hypotheses for *P* which needed to be shared with *Q* in order for ${\hat{cFDR}}^{n}$ to have an advantage in TDR over only considering *P*. When no non-null hypotheses are shared, the values *q_i_* confer no information on $H_{0}^{P}$ , so we expect that use of v-values will add only noise and the TDR of the p-value will be larger than that of the v-value. We plotted the difference in TDR between ${\hat{cFDR}}^{n}$ (using leave-one-out v-values) and p-values *p_i_* against $n_{1}^{pq}/\left(n_{1}^{pq}+n_{1}^{p}\right)$ and found that this difference became positive when around 20% of hypotheses were shared (Figure 7). This figure is dependent on our simulation parameters: a smaller percentage of hypotheses may lead to an advantage of ${\hat{cFDR}}^{n}$ if, for instance, effect sizes were very large.

### 5.5 Iterated cFDR

Since our proposed method for type-1 error rate control maps p-value/covariate pairs to v-values preserving the p-value property, we are free to use the resultant v-values in a second cFDR-based analysis against a second covariate. This enables immediate and simple adaptation to a setting in which more than one set of covariates are available. In our motivating example, this would allow us to subsequently ’condition’ on other potentially related diseases as well as OCA.

We simulated a set of p-values {*p*} = {*p_i_*,*i* ∈ 1… 1000}, with 100 true associations ( $H_{1}^{p}$ ) in which p-values were sampled from 2Φ(–|*N*(0,3^2^)|) (where Φ is the normal CDF) and 900 non-associations ( $H_{0}^{p}$ ) in which p-values were sampled from *U*(0,1). We then similarly simulated sets of covariates $\left\{q^{j}\right\}=\left\{q_{i}^{j},i\in 1\ldots 1000\right\}$ with 100 true associations $H_{1}^{q}$ , which for even *j* were randomly spaced amongst the 1000 variables (uninformative covariates) and for odd *j* overlapped more-than-randomly with associations with principal p-values (informative covariates), with around 54 shared associations on average (strictly, such that $Pr\left(H_{1}^{q}|H_{1}^{p}\right)=15Pr\left(H_{1}^{q}|H_{0}^{p}\right)).$

**Figure 8:**
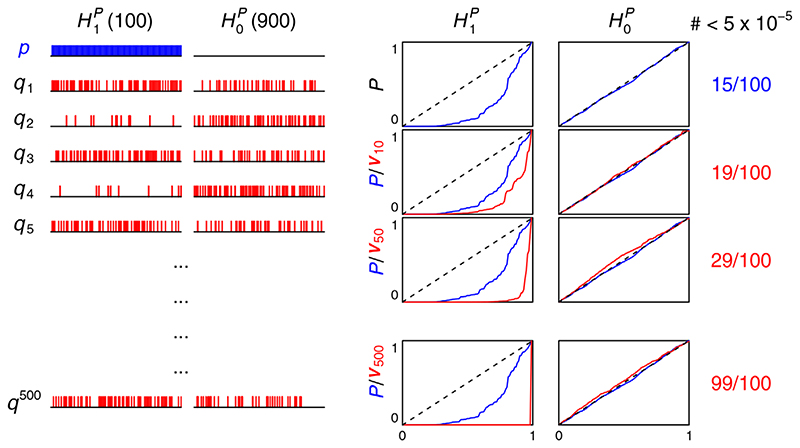
Iterated cFDR. The left part of plot shows where non-null hypotheses fall in *p*/*q^J^* (blue vertical lines for *p*, red for *q_j_*). Non-null hypotheses are shared more-than-randomly in only every second set *q^j^*. The right part shows *p*/*v_j_* values (blue/red lines, respectively) plotted in an ascending order under $H_{1}^{p}$ , $H_{0}^{p}$ , and the number of *p*/*v_j_* values which reach the Bonferroni-corrected significance

Starting with *v*_0_ = *p*, we conditioned on each set of {*q^j^*} in succession, so ν_*i* + 1_ = ν(ν_*i*_,*q^i^*). We used ${\hat{cFDR}}^{n}$ as an estimator and used leave-one-out v-values. Originally 19 of 100 null hypotheses were correctly rejected using *p* alone (*p* < 5 × 10^–5^ = 0.05/1000). On repeated conditioning, almost all v-values when *H^p^* = 1 tended to 0: 99 null hypotheses were correctly rejected using *v*_500_. Under $H_{0}^{p}$ , v-values remained uniform on (0,1) (Figure 8). This indicated the potential to greatly strengthen the power of a high-dimensional association analysis by repeated conditioning in this manner, even when only half of the sets of covariates are informative.

### 5.6 Summary of BRCA analysis

Finally, we return to the motivating example. cFDR rejects more null hypotheses for BRCA (724) than B-H on BRCA data alone (678, Figure1a) or the subset of variables with OCA association (280, Figure 1b). The procedure is asymmetrical in that it will not reject a BRCA null hypothesis for a low OCA p-value alone and can readily be reversed: Supplementary Figure 6 shows a similar analysis analysing association with OCA.

## 6 Discussion

We present an improvement to the cFDR method, a widely used procedure in genetic discovery. Our new methods essentially involve computing an analogy of the p-value corresponding to the ranking of hypotheses defined by the cFDR estimator. Our method enables the cFDR to be used definitively in the discovery phase of -omics studies with control of a type-1 error rate. The general procedure of multiple p-value testing with a covariate has wide scientific application; see Du et al. (2014), Alishahi et al. (2016) and Li and Barber (2016) for example.

The $\hat{cFDR}$ and ${\hat{cFDR}}^{n}$ estimators make no distributional assumptions on *P*, *Q*. The type-1 error rate controlling method requires modelling of the PDF of *P*, $Q|H_{0}^{p}$ , but this requires approximating a univariate PDF $Q|H_{0}^{p}$ . Furthermore, this PDF is only used as an integrand rather than for direct point estimates. It is reasonable to expect that for approximations to complex PDFs, relative average errors over intervals will be smaller than relative errors at individual points; parametric approximations tend to be smoother than the true distribution at a fine scale, and KDE-based approximations rougher. An obvious shortcoming of cFDR-based methods is the lack of asymptotic optimality. Methods based on consistent estimators of *f_0_*/*f* will eventually outperform any estimator of *F_0_*/*F* for large enough *n* (see Supporting Information, Section 1.5). However, the ECDF-based cFDR estimator was far stronger than PDF-based estimators at the values of *n* we simulated at (10^3^ – 10^4^). In practical terms, it is important to note that *n*, being the number of variables, cannot generally be increased indefinitely, as opposed to, for instance, sample size. Essentially, the fitting of L-curves corresponds to a procedure by which the similarity between *P*, *Q* is assessed, and the degree of modulation when moving from *p* to *v* values corresponds to this similarity. Moreover, this assessment of similarity occurs intrinsically on the basis of the joint CDF rather than relying on a parametric description.

L-curves may not change monotonically with *Q*; that is, it may be possible to reject a null hypothesis with p-values (*p*, *q_1_*) and not reject a null hypothesis with p-values (*p*, *q*_2_), *q*_2_ < *q*_1_ (see the lower-left panel of Figure 1). It would be possible within our framework of FDR control (Theorem 3.1) to force L-curves to be monotonic with *Q*, and indeed since this would result in straight-up-and-down segments on L-curves, the loss of power due to noise when *P* and *Q* are unrelated (Figure 7) may be reduced in this case. However, non-monotonicity of L-curves is potentially advantageous in a biostatistical setting. Between TWAS or GWAS for similar diseases, it may be the case that shared non-null hypotheses have ’moderately’ small p-values, corresponding to common general shared medium-risk pathological causes, but non-shared non-null hypotheses have ‘extremely’ small p-values, corresponding to specific high-risk pathologies. Non-monotonic L curves allow this effect to be modelled. Unrestricted L-curves also allow use to be made of q-values such that $Pr\left(H_{0}^{P}|Q=q\right)$ is lower with low *q,* rather than higher: we show this in Table 2, row ‘negative information’.

Our proposed ‘iterated cFDR’ procedure can be thought of as a meta-analysis of a series of experiments *E_P_*,*E_Q1_*,*E_Q2_*, … giving rise to p-value sets {*p*}, $\left\{q_{i}^{1}\right\}$ , $\left\{q_{i}^{2}\right\}$ ,… when only the first set ({*p*}) is known to test the correct hypotheses; that is, be *U*(0,1) for null hypotheses. It enables us to find the set of non-null hypotheses corresponding to $E_{P}\left(\;\text{denoted}\;\left\{H_{1}^{p}\right\}\right)$ , even though the set of non-null hypotheses corresponding to $E_{Q_{j}}\left(\;\text{denoted}\;\left\{H_{1}^{Q_{j}}\right\}\right)$ may only partly overlap $\left\{H_{1}^{P}\right\}$ , may contain hypotheses not in $\left\{H_{1}^{p}\right\}$ and (half the time) may even carry no information about $H_{1}^{p}$ at all. This could be used to refine the set of association statistics {*p_i_*} for a disease of interest by using sets of association statistics $\left\{q_{i}^{1}\right\}$ , $\left\{q_{i}^{2}\right\}$ at the same variables for a range of separate traits. It could also be used to improve power when repeating an -omics study in a new ethnic group by levering on previous studies in different ethnicities.

In summary, our method improves the power of cFDR analyses and allows it to be used confidently in the setting of multiple hypothesis testing. This can enable more efficient use of data, and more information to be gained from the same datasets.Our method contributes to a set of tools for high-dimensional statistical analysis and has wide application across a range of fields in biomedicine and elsewhere.

## Supplementary Material

Supplementary material

Supplementary material 2

Appendix

## Figures and Tables

**Table 1:** Variables used in simulations

Variable	Description	Sampling distribution
*n*	Total number of variables	10^*U*(3,4)^ (rounded)
$n_{1}^{pq}$	Number of variables assoc. with *P*, *Q*	*U*(0, 200) (rounded)
$n_{1}^{p}$	Number of variables associated with *P*	*U*(0, 200) (rounded)
$n_{1}^{q}$	Number of variables associated with *Q*	*U*(0, 200) (rounded)
*S_p_*	Scale for distribution of *P* (see below)	$U\left(\frac{3}{2},3\right)$
*s_p_*	Scale for distribution of *Q*	$U\left(\frac{3}{2},3\right)$
*d*	Form of alternative distributions	1: Normal, 2: t (3df), 3: Cauchy, eq. prob.

**Table 2:** FDR and TDR of p-value, ${\hat{cFDR}}^{n}$ , and oracle cfdr (best possible procedure) using leave-one-out v-values (Equation (25)) for a range of simulation parameters, controlling FDR at α. = 0.1. All descriptions are relative to ‘Reference,’ which has the following parameter values: *n* = 5000, $n_{1}^{p}=n_{1}^{q}=n_{1}^{pq}=100$ , *s_p_* = *s_q_* = 2, *d* = 2. TDR is undefined if $n_{1}^{p}+n_{1}^{pq}=0$ . ‘Negative information’ means *fewer-than* random shared associations, rather than more $\left(n_{1}^{p}=n_{1}^{q}=2000,n_{1}^{pq}=0\right)$ . Complete parameter values, confidence intervals and more detailed results are shown in Supplementary Table 1

Description	TDR(P)	FDR(cFDR)	TDR(cFDR)	TDR(oracle)
Reference	0.194	0.0955	0.208	0.212
No effects		0.0973		
Weak effects	0.00803	0.0814	0.00795	0.00904
Large variance in effect sizes	0.493	0.0957	0.517	0.527
Larger n	0.173	0.0995	0.189	0.193
Smaller n	0.26	0.0795	0.265	0.269
No non-null shared hypotheses	0.188	0.102	0.178	0.187
All non-null hypotheses shared	0.195	0.0963	0.26	0.309
Negative information	0.302	0.0585	0.314	0.319
Block correlation	0.2	0.0974	0.213	0.217
Equicorrelation	0.191	0.0897	0.205	0.209

## Data Availability

All functions necessary to apply the methods detailed in this work are available in the R package https://github.com/jamesliley/cfdr A full pipeline to generate the results in this paper is available in the git repository https://github.com/jamesliley/cfdr_pipeline

## Data Availability Statement

The data that support the findings of this study are openly available in TWAS-HUB at http://twas-hub.org/traits/.
