# Supplementary material for: Accurate error control in high-dimensional association testing using conditional false discovery rates
Source: Biom J. Author manuscript; Available in PMC 2022 Feb 3. (PMC7612315; doi:10.1002/bimj.201900254)
Supplement: Supplementary material [file EMS140914-supplement-Supplementary_material.pdf]

Accurate error control in high dimensional association testing using conditional false discovery rates

Supplementary material and figures

James Liley and Chris Wallace

1 Supplementary material

1.1 TWAS details

TWAS test association of gene expression (a biologically interpretable quantity) with some trait, when the trait and expression have not been measured on the same individuals, by using a reference expression-quantitative trait locus (eQTL) study and genome-wide association study (GWAS). A TWAS firstly uses eQTL data to learn rules to predict mRNA expression levels in a given tissue according to individual genotype, then applies these rules to predict expression for each individual in a GWAS, and finally compares these predicted expression levels across the GWAS trait of interest [Gusev et al., 2016].

The results from a TWAS are a set of p-values corresponding to tissue-gene pairs. The object is to find which tissue-gene pairs are associated with the disease under consideration; that is, which p-values come from a distribution other than $U(0, 1)$. In general, such tissue-gene pairs are a small proportion of all those considered. We consciously ignore any prior information which could be derived from tissues likely to be BRCA or OCA associated, for purposes of demonstration.

We considered TWAS datasets for breast cancer (BRCA, [Michailidou et al., 2017]) and ovarian cancer (OCA, [Phelan et al., 2017]), containing tests for varying numbers of genes across 54 tissues. BRCA and OCA have considerable phenotypic overlap [Greene et al., 1984], and we may hope that summary statistics for one disease may be useful for leverage in association analyses of the other. We considered RNA-tissue pairs available in both datasets, restricting our analysis only to pairs in which RNA expression was predicted using data from the GTEx consortium, comprising a total of $n = 80222$ hypotheses.

Given the GWAS-scale dimensionality of testing, we chose a conservative FDR control level $\alpha = 1 \times 10^{-6}$. We used both $\widehat{cFDR}$ and $\widehat{cFDR}^n$ to generate v-values, and used the block-out method with blocks assigned according to genes, so expression levels for each gene were assigned a separate block (for 11327 folds in total).

1.2 Correlation of $P, Q|H_0^p$

Throughout this paper we have largely assumed that $P \perp\!\!\!\perp Q|H_0^p$, but methods can be easily adapted as long as the distribution $P \perp\!\!\!\perp Q|H_0^p$ is known (or we are happy to assume it is known). An example where this occurs is in the GWAS literature in which the studies giving rise to p_i and q_i share samples (usually control samples), which induces a known correlation between P and Q under H_0^p.

This can generally be managed by using the true f_0 (or an approximation allowing for dependence of P, Q under H_0^p) in equation (19) in the main paper and accounting for the true f_0 in the computation of $Pr(P \leq p|Q \leq q, H_0^p)$ necessary to estimate cFDR (equation (6) in main paper) where it is otherwise equal to p under assumption (1) in the main paper.

We demonstrate how to approximate $Pr(P \leq p|Q \leq q, H_0^p)$ and f_0 in the specific case of shared controls in a previous paper [Liley and Wallace, 2015].

1.3 Simulations

1.4 Alternative cFDR estimators, and estimators of cfdr

In this section, we introduce new estimators of the cFDR $Pr(H_0^p|P \leq p, Q \leq q)$ and cfdr $Pr(H_0^p|P = p, Q = q)$, for use in simulations as detailed in section 5 of the main paper.

The main incentive for different estimators of cFDR is the tendency for the ECDF based estimator (equation (6) in the main paper) to have marked discontinuities at extremes of the unit square. This is illustrated in figure 1. The main incentive for estimators of cfdr is to allow comparison of PDF- and CDF- based estimators of the optimum rejection region detailed in section 2.2 in the main paper.

1.4.1 Parametric estimators for cFDR and cfdr

The estimate $\widehat{cFDR}$ is based on empirical quantities estimated directly by empirical CDFs of (P, Q). We consider here estimators based on approximating the joint distribution of P, Q using a bivariate mixture-normal parametrisation. This estimator enforces continuity of $\widehat{cFDR}$ on the open unit square, and is robust to small deviations in p-values, overcoming the effect detailed in figure 1. It is easiest to visualise parametrisations as distributions over the unsigned Z scores $(Z_p, Z_q) = \left(-\Phi^{-1}(P/2), -\Phi^{-1}(Q/2)\right)$ with $\Phi^{-1}(x)$ denoting the standard normal quantile function at x.

We use a parametrisation with seven parameters: $(\pi_0,\ \pi_1,\ \pi_2,\ \tau_1,\ \tau_2,\ \sigma_1,\ \sigma_2)$, which parametrise a four-part bivariate mixture-Gaussian distribution over the $(+, +)$ quadrant

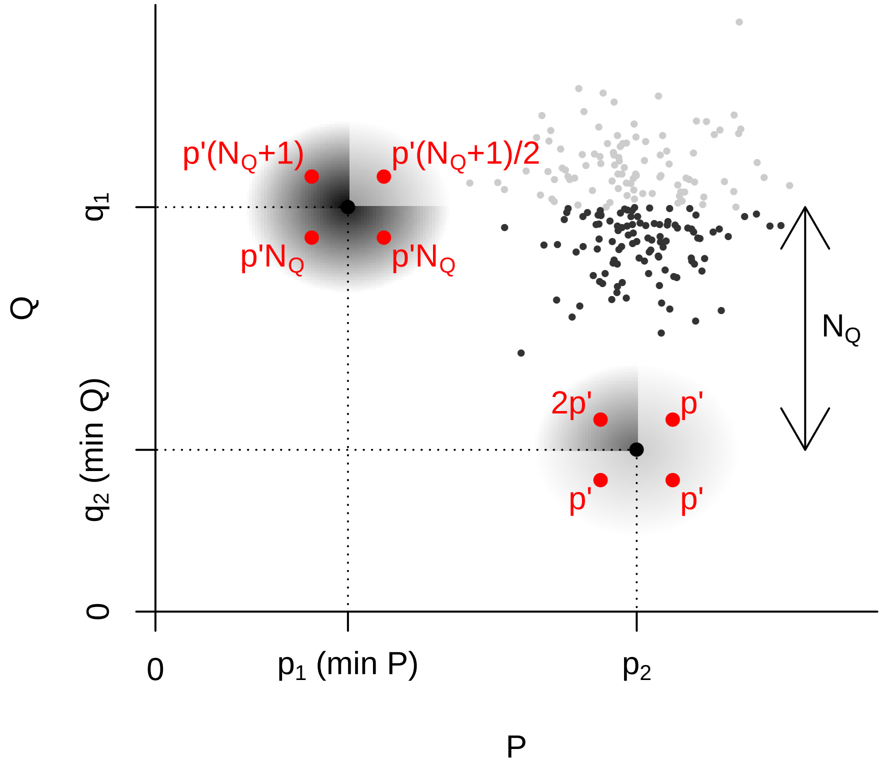

Figure 1: Dependence of $c\widehat{FDR}$ values on location of nearby points. In this example, we denote by (p_1, q_1), (p_2, q_2) the points at the (unique) left and lower extremes of the observed p-value distribution respectively; that is, $p_1 = \min(p_i)$, $q_2 = \min(q_i)$. We set N_Q as the number of points with $q_1 \leq q_i \leq q_2$ (small black points). If we add a test point (p', q') (shown in red) in a small neighbourhood of either (p_1, q_1) or (p_2, q_2), the estimated $c\widehat{FDR}_{S+(p',q')}(p', q')$ (shown in red next to the point) differs by a factor of 2 in different quadrants of the neighbourhood.

with PDF:

$$
\begin{aligned}
f^p(x,y) = 4\pi_0 N_{\Sigma_0}(x,y) \\
+ 4\pi_1 N_{\Sigma_1}(x,y) \\
+ 4\pi_2 N_{\Sigma_2}(x,y) \\
+ 4(1-\pi_0-\pi_1-\pi_2) N_{\Sigma_3}(x,y)
\end{aligned} \tag{1}
$$

where $N_\Sigma(x,y)$ is the PDF of the bivariate normal distribution centred at the origin with variance Σ, the factor of 4 is due to to only unsigned Z-scores being used, and

$$
\Sigma_0 = I_2 \qquad \Sigma_1 = \begin{pmatrix} \tau_1^2 & 0 \\ 0 & 1 \end{pmatrix} \qquad \Sigma_2 = \begin{pmatrix} 1 & 0 \\ 0 & \sigma_1^2 \end{pmatrix} \qquad \Sigma_3 = \begin{pmatrix} \tau_2^2 & 0 \\ 0 & \sigma_2^2 \end{pmatrix} \tag{2}
$$

This model specifies a proportion π_1 of study variables to be associated only with the trait of interest P (with $SD(Z_P) = \tau_1$), a proportion π_2 to be associated only with the second trait Q (with $SD(Z_Q) = \sigma_1$), and a proportion $(1-\pi_0-\pi_1-\pi_2)$ to be associated with both ($Var(Z_P, Z_Q) = \Sigma_3$). We can now write:

$$
\begin{aligned}
f_0^p(x,y) &= f(Z_P = x, Z_Q = y | H_0^p) \\
&= 4\frac{\pi_0}{\pi_0+\pi_2} N_{\Sigma_0}(x,y) \\
&\quad + 4\frac{\pi_2}{\pi_0+\pi_2} N_{\Sigma_2}(x,y)
\end{aligned} \tag{3}
$$

We allow different values of σ_1, σ_2 and τ_1, τ_2 to allow for potentially different reasons for shared (both P and Q) and independent (P XOR Q) associations.

Maximum-likelihood estimates of parameters $(\pi_0, \pi_1, \pi_2, \tau_1, \tau_2, \sigma_1, \sigma_2)$ can be obtained using an E-M algorithm [Dempster et al., 1977]. Given these and corresponding estimates $\widehat{f^p}$, $\widehat{f_0^p}$ of f^p, f_0^p and $\widehat{F^p}$, $\widehat{F_0^p}$ of F^p, F_0^p we can then define an estimate of cFDR (implicitly conditioning on parametric assumptions):

$$
\begin{aligned}
\widehat{cFDR}_X^p(p,q) &= \frac{Pr(P \leq p | Q \leq q, H_0^p)}{Pr(P \leq p | Q \leq q)} Pr(H_0^p | Q \leq q) \\
&= \frac{\widehat{F_0^p}(-z_p, -z_q)}{\widehat{F^p}(-z_p, -z_q)} \frac{\widehat{F^p}(-z_p, -\infty)}{\widehat{F_0^p}(-z_p, -\infty)} Pr(H_0^p | Q \leq q)
\end{aligned} \tag{4}
$$

The quantity $Pr(H_0^p | Q \leq q)$ may be estimated up to directly from parametric assumptions as

$$
\begin{aligned}
Pr(H_0^p | Q \leq q) &= \frac{Pr(Q \leq q | H_0^P)}{Pr(Q \leq q} Pr(H_0^p) \\
&\propto \frac{\widehat{F_0^p}(-z_p, -\infty)}{\widehat{F^p}(-z_p, -\infty)}
\end{aligned} \tag{5}
$$

or may be estimated on the basis of the empirical CDF of $Q|P > 1/2$ as per equation (7) in the main paper. We found that the performance of $\widehat{cFDR}^p$ was stronger when using the empirical estimate in equation (7) than the parametric estimate in equation (5) (figure 2)

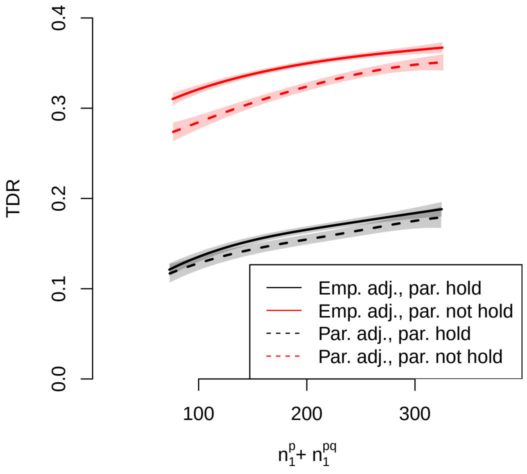

Figure 2: Performance (TDR) of parametric cFDR estimator against $n_1^p + n_1^{pq}$, using either a parametric ('Par. adj') or empirical ('Emp. adj') estimate of $Pr(H_0^P|Q \leq q)$, and separating cases where parametric assumptions hold ('par. hold') or do not hold ('par. not hold'). See section 5 for further details. Shaded regions show 95% pointwise confidence envelopes. The empirical estimate leads to better performance when parametric assumptions are not satisfied, and equivocal performance when they are.

The local cfdr can be readily estimated as

$$\widehat{cfdr}^p_X(p,q) = \frac{f(Z_P = z_p, Z_Q = z_q|H_0^p)}{f(Z_P = z_p, Z_Q = z_q)} Pr(H_0^p) \\ \propto \frac{\widehat{f_0^p}(z_P, z_Q)}{\widehat{f^p}(z_P, z_Q)} \quad (6)$$

where X in this case is the set of points used in the estimation of parameters.

We note that estimator (4) implicitly includes an estimate of $\widehat{Pr}_X(H_0^p|Q \leq q)$, computed from parameter estimates. However, we found that performance of the estimator was improved when equation (7)

1.4.2 KDE-based estimators for cFDR and cfdr

To avoid distributional assumptions while maintaining a smooth form for the density of P, Q, a second estimator of $Pr(P \leq p|Q \leq q)$ can be derived from a two-dimensional kernel density estimator (KDE). We had no reason to prefer any kernel function over another, so opted to use a normal kernel with constant variance I_2. The PDF corresponding to Z_p, Z_q at x, y was modelled in the usual way as

$$f^k(x,y) = \frac{1}{n}\sum_i \frac{1}{\sigma_p\sigma_q}\phi\left(\sqrt{\left(\frac{x-\{-\Phi^{-1}(p_i/2)\}}{\sigma_p}\right)^2 + \left(\frac{y-\{-\Phi^{-1}(q_i/2)\}}{\sigma_q}\right)^2}\right) \quad (7)$$

where $\phi(.)$ is the standard normal density. Values σ_p and σ_q are determined using a standard method based on the observations $p_i, q_i \in X$ [Sheather and Jones, 1991].

Unlike the parametric estimate above, this does not intrinsically specify the density of $P, Q|H_0^p$. We thus incorporate the estimator $\widehat{Pr}_X(H_0^p|Q \leq q)$ from equation (7) in the main paper, and write (implicitly conditioning on correctness of approximations)

$$\begin{aligned} \widehat{cFDR}^k_X(p,q) &= \frac{Pr(P \leq p|Q \leq q, H_0^p)}{Pr(P \leq p|Q \leq q)} Pr(H_0^p|Q \leq q) \\ &= \frac{pPr(Q \leq q)}{Pr(P \leq p, Q \leq q)} Pr(H_0^p|Q \leq q) \\ &= \frac{p\int_{z_q}^{\infty}\int_0^{\infty} f^k(x,y)dxdy}{\int_{z_q}^{\infty}\int_{z_p}^{\infty} f^k(x,y)dxdy}\widehat{Pr}_X(H_0^p|Q \leq q) \end{aligned} \quad (8)$$

where X is the set of points used in the KDE in equation (7). We note that this estimator converges to $\widehat{cFDR}^n$ as $\sigma_p, \sigma_q \to 0$.

Estimating local cfdr using KDEs requires estimation of $f(Z_P = z_p, Z_Q = z_q|H_0^p)$. We use assumption (1) from the main paper, and as for equation (7) in the main paper we assume that $Q|H_0^p \sim Q|P > 1/2$. We then fit a one-dimensional KDE to the values $z_{q_i}|p_i > 1/2$, and denote the resultant function of q as $\hat{f_0^k}(q)$. We then write (conditioning on assumptions)

$$\begin{aligned} \hat{f}_0(p,q) &= p\hat{f_0^k}(q) \\ \widehat{cfdr}^k(p,q) &= \frac{f(P=p, Q=q|H_0^p}{f(P=p, Q=q)} \\ &= \frac{\hat{f}_0(p,q)}{f^k(z_p, z_q)} \end{aligned} \quad (9)$$

1.5 Analysis of cFDR estimators

We required that all estimators be nonincreasing in p, so all were censored when generating L-curves or designing rejection procedures in the same way as in (9) and (16) for $\widehat{cFDR}$ in

the main paper.

We show in figures 3, 4, 5 a series of plots at different values of n which indicate the behaviour of L-curves as n increases. In all cases, P, Q are sampled under the parametric assumptions in supplementary section 1.4.1, with $(\pi_0,\ \pi_1,\ \pi_2,\ \tau_1,\ \tau_2,\ \sigma_1,\ \sigma_2) = (0.7, 0.1, 0.15, 1.5, 2, 1.5, 2)$. Curves are drawn through $(0.1, 0.1)$, which would generally corresponds to a very high FDR level, so oracle PDF and oracle CDF curves are markedly different.

Importantly, $\widehat{cfdr}^p$ and $\widehat{cfdr}^k$ converge to the optimal rejection region (oracle PDF) while $c\widehat{FDR}$, $c\widehat{FDR}^p$ and $c\widehat{FDR}^k$ do not. However, the estimates of the latter are less noisy.

References

Arthur P Dempster, Nan M Laird, and Donald B Rubin. Maximum likelihood from incomplete data via the EM algorithm. *Journal of the Royal Statistical Society, series B (methodological)*, 39(1):1–38, 1977.

Mark H Greene, Jeffrey W Clark, and Douglas W Blayney. The epidemiology of ovarian cancer. In *Seminars in oncology*, volume 11, pages 209–226. Elsevier, 1984.

Alexander Gusev, Arthur Ko, Huwenbo Shi, Gaurav Bhatia, Wonil Chung, Brenda WJH Penninx, Rick Jansen, Eco JC De Geus, Dorret I Boomsma, Fred A Wright, et al. Integrative approaches for large-scale transcriptome-wide association studies. *Nature genetics*, 48(3):245, 2016.

James Liley and Chris Wallace. A pleiotropy-informed Bayesian false discovery rate adapted to a shared control design finds new disease associations from GWAS summary statistics. *PLOS Genetics*, 2015.

Kyriaki Michailidou, Sara Lindström, Joe Dennis, Jonathan Beesley, Shirley Hui, Siddhartha Kar, Audrey Lemaçon, Penny Soucy, Dylan Glubb, Asha Rostamianfar, et al. Association analysis identifies 65 new breast cancer risk loci. *Nature*, 551(7678):92, 2017.

Catherine M Phelan, Karoline B Kuchenbaecker, Jonathan P Tyrer, Siddhartha P Kar, Kate Lawrenson, Stacey J Winham, Joe Dennis, Ailith Pirie, Marjorie J Riggan, Ganna Chornokur, et al. Identification of 12 new susceptibility loci for different histotypes of epithelial ovarian cancer. *Nature genetics*, 49(5):680, 2017.

Simon J Sheather and Michael C Jones. A reliable data-based bandwidth selection method for kernel density estimation. *Journal of the Royal Statistical Society. Series B (Methodological)*, pages 683–690, 1991.

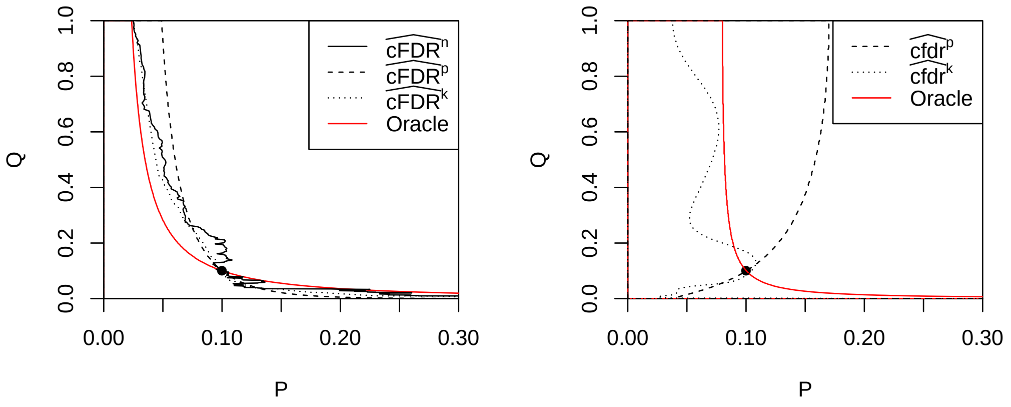

Figure 3: L-curves using various methods for cFDR estimation; n=1000. CDFs on left, PDFs on right.

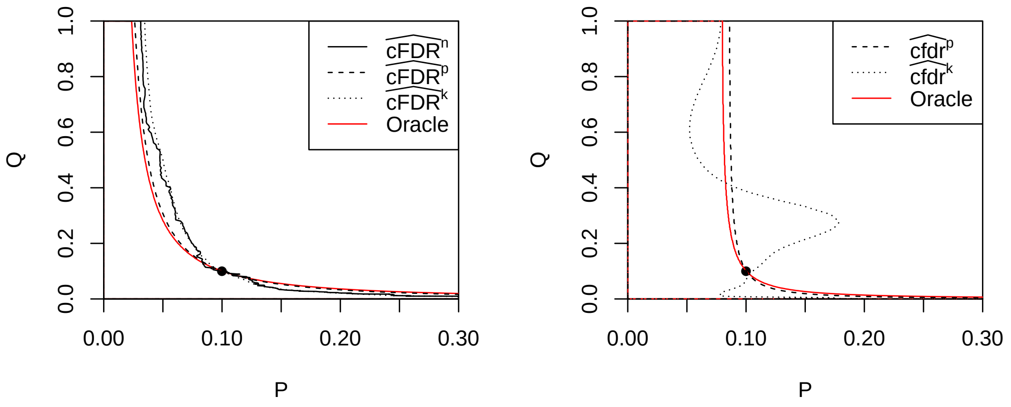

Figure 4: L-curves using various methods for cFDR estimation; n=10000. CDFs on left, PDFs on right.

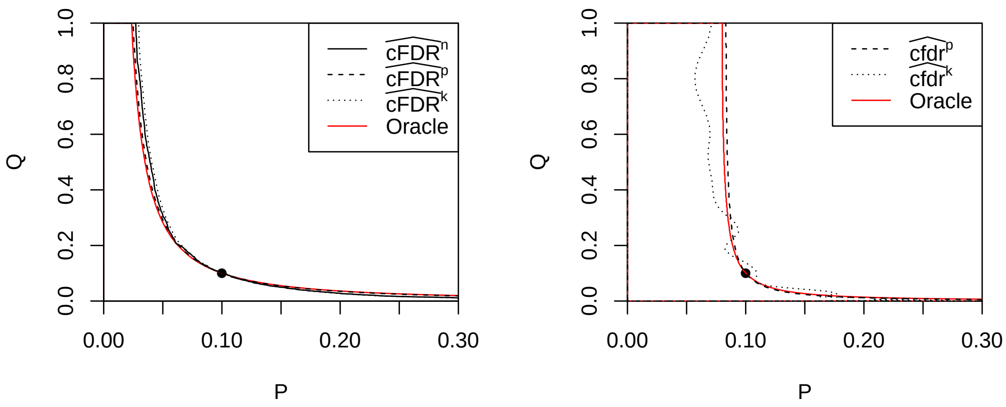

Figure 5: L-curves using various methods for cFDR estimation; n=100000. CDFs on left, PDFs on right.

2 Supplementary figures

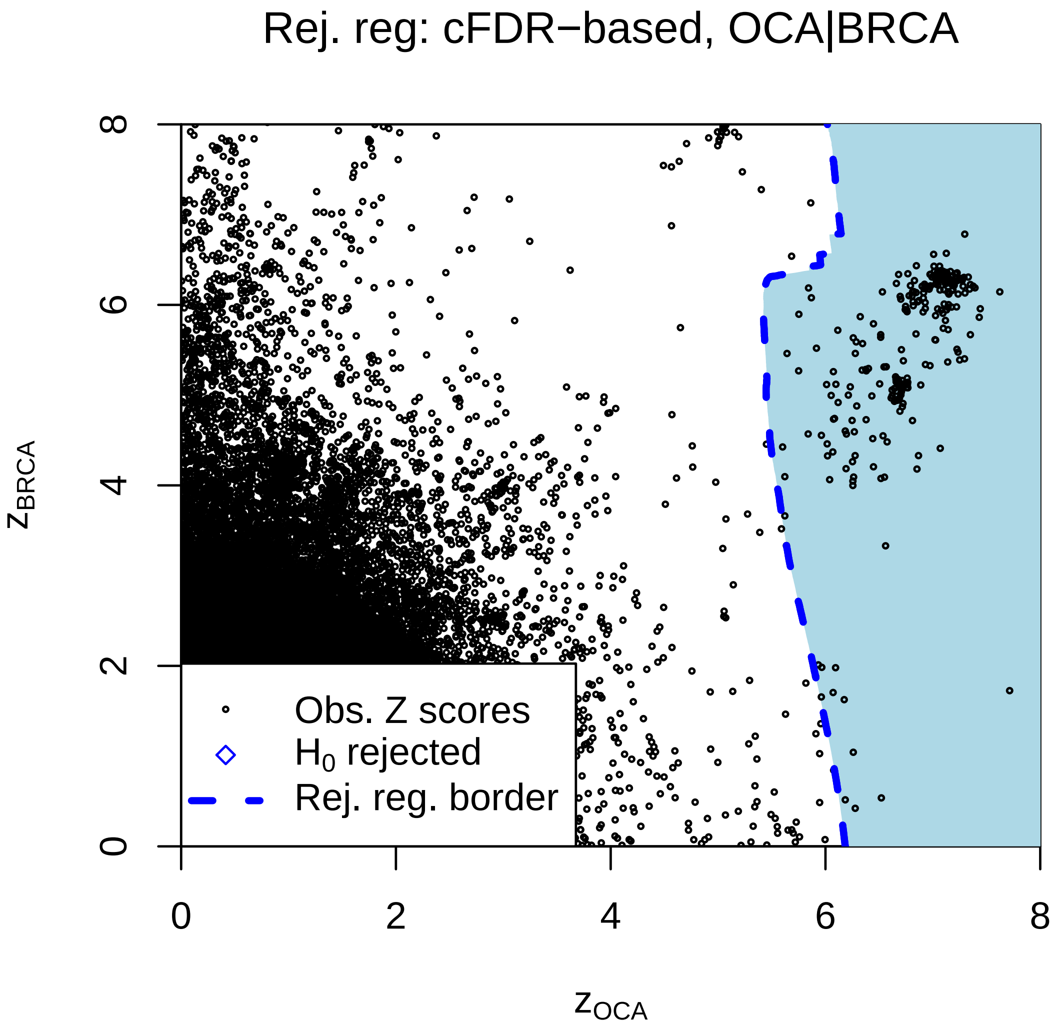

Figure 6: Association analysis of OCA using test statistics for BRCA as covariates. Variables and methods are similar to panel C in figure 1 in the main paper.

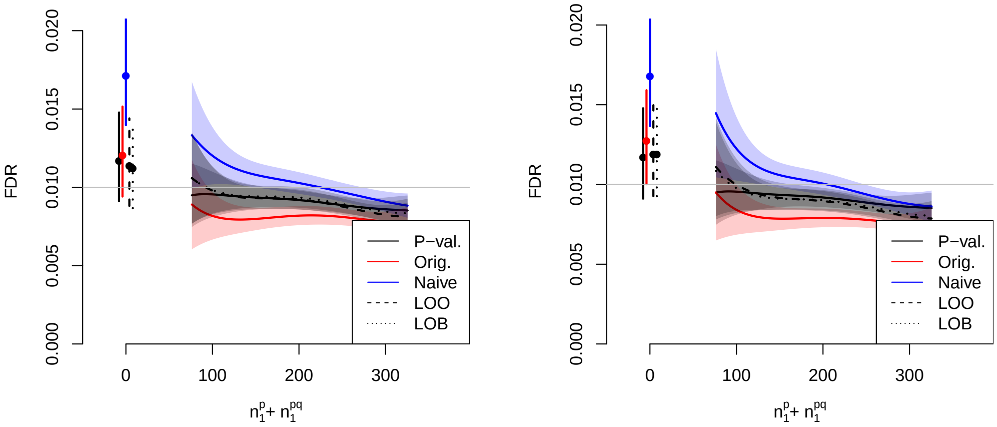

Figure 7: FDR control of various methods against $n_1^p + n_1^{pq}$, the total number of variables associated with P (the primary study under consideration). The horizontal line shows $\alpha = 0.01$, the desired FDR control level. Simulations in the left panel integrate L-regions over the the true distribution f_0; simulations in the right panel integrate over the estimated distribution as per equation (33). Shaded regions indicate 95% confidence envelopes. Curves show moving weighted averages using a Gaussian kernel with SD 15% of the X axis range. Lines on the left indicate FDR control with $n_1^p + n_1^{pq} = 0$.

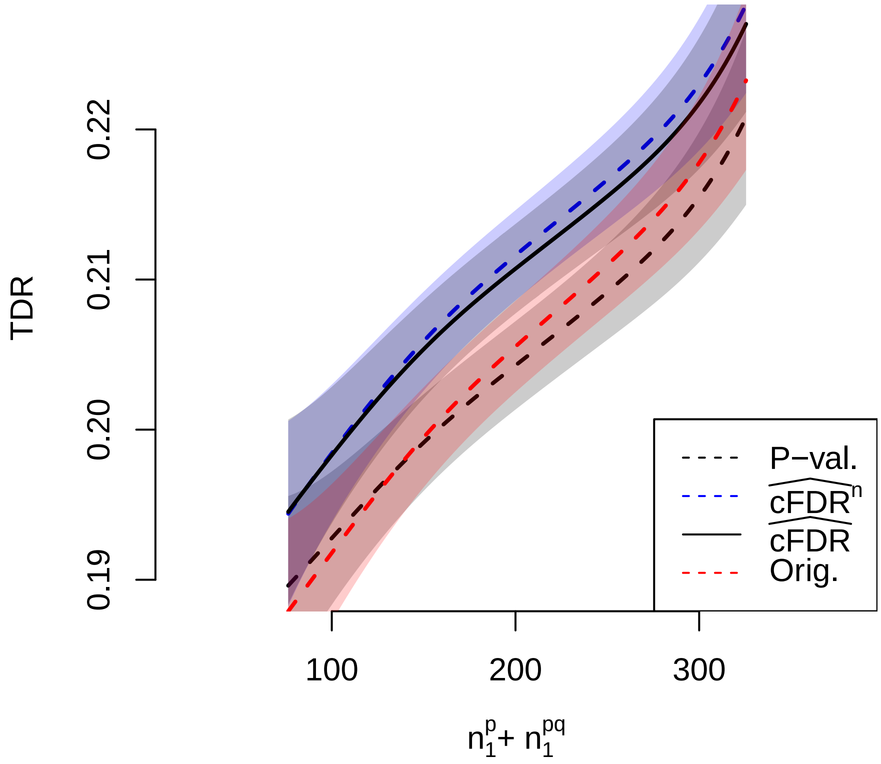

Figure 8: TDR of various methods against $n_1^p + n_1^{pq}$, the total number of variables associated with P (the primary study under consideration), at FDR control level $\alpha = 0.01$. Shaded areas show 95% pointwise confidence envelopes. Curves show moving weighted averages using a Gaussian kernel with SD 15% of the X axis range.

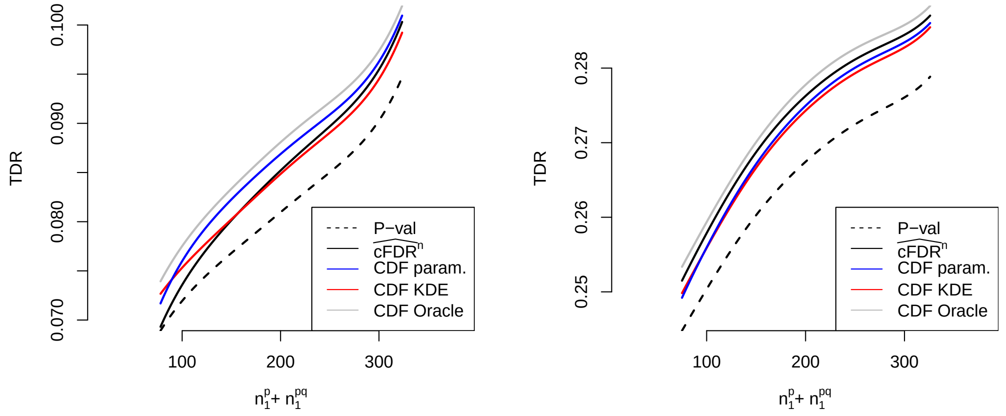

Figure 9: TDR of various methods against $n_1^p + n_1^{pq}$, the total number of variables associated with P (the primary study under consideration), restricting to simulations in which parametric assumptions were satisfied (left panel) or were not satisfied (right panel), at FDR control level $\alpha = 0.01$. Curves show moving weighted averages using a Gaussian kernel with SD 3/10 of the X axis range.

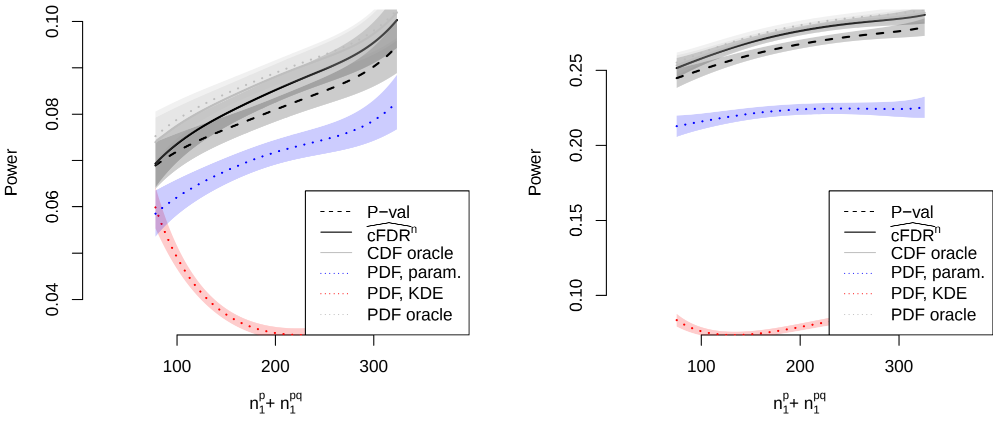

Figure 10: TDR of PDF-based methods against $n_1^p + n_1^{pq}$, the total number of variables associated with P (the primary study under consideration), controlling FDR at $\alpha = 0.01$. In the left panel, parametric assumptions were satisfied (ie $d = 1$ in table 1) and in the right panel, they are not ($d = 2, 3$). Shaded regions show pointwise 95% confidence intervals. Curves show moving weighted averages using a Gaussian kernel with SD 15% of the X axis range.

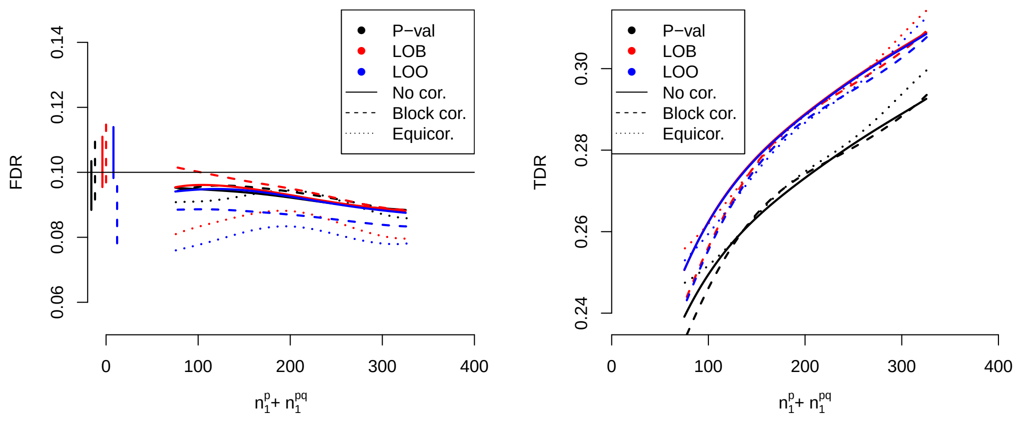

Figure 11: FDR (left) and TDR (right) of FDR-controlling methods leave-out-block (equation (26)) and leave-one-out (equation (25)) applied to $c\widehat{FDR}^n$, and the BH procedure applied to p-values, under several models of correlation between observations ($\rho = 0.1$). Confidence envelopes are omitted for visual clarity. Vertical lines show FDR with 95% confidence intervals at $n_1^p + n_1^{pq} = 0$. Curves show moving weighted averages using a Gaussian kernel with SD 15% of the X axis range.

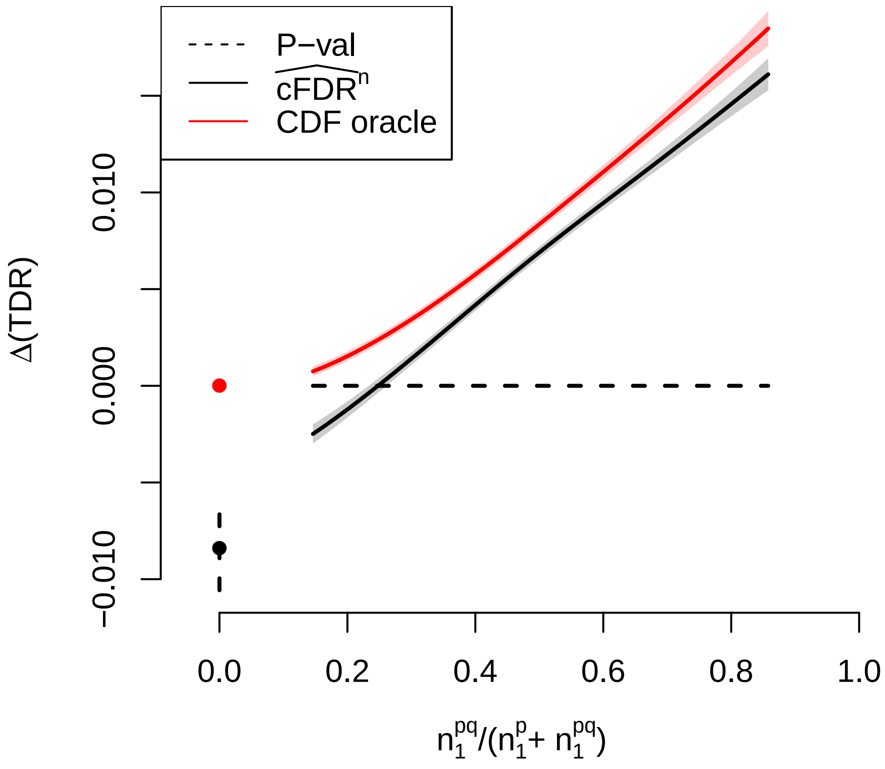

Figure 12: Difference in TDR between $\widehat{cFDR}^n$ (assessed by leave-one-out v-values) and p-values, controlling FDR at $\alpha = 0.01$, against $n_1^{pq}/(n_1^{pq} + n_1^p)$ (proportion of non-null hypotheses for P which are shared with Q). The performance of the oracle CDF method is shown for comparison. Shaded areas show pointwise 95% confidence intervals. Points and lines at the leftmost edge show TDR values and 95% confidence intervals when $n_1^{pq}/(n_1^{pq} + n_1^p) = 0$. Curves show moving weighted averages using a Gaussian kernel with SD 15% of the X axis range.

3 Supplementary tables

Description	n	n_1^p	n_1^q	n_1^{pq}	s_p	s_q	d	ρ
Reference	5000	100	100	100	2	2	2	
No effects	5000	0	0	0	2	2	2	
Weak effects	5000	100	100	100	1.5	1.5	1	
Large variance in effect sizes	5000	100	100	100	3	3	3	
Larger n	10000	100	100	100	2	2	2	
Smaller n	1000	100	100	100	2	2	2	
No non-null shared hypotheses	5000	150	150	0	2	2	2	
All non-null hypotheses shared	5000	0	0	200	2	2	2	
Negative information	5000	2000	2000	0	2	2	2	
Block correlation	5000	100	100	100	2	2	2	0.05
Equicorrelation	5000	100	100	100	2	2	2	0.05

Description	FDR(P)	TDR(P)
Reference	0.096 (0.0929,0.099)	0.194 (0.192,0.196)
No effects	0.0881 (0.0704,0.106)	
Weak effects	0.1 (0.0868,0.113)	0.00803 (0.00748,0.00857)
Large variance in effect sizes	0.0956 (0.0938,0.0975)	0.493 (0.491,0.496)
Larger n	0.1 (0.0971,0.103)	0.173 (0.171,0.175)
Smaller n	0.08 (0.0777,0.0824)	0.26 (0.258,0.263)
No non-null shared hypotheses	0.0991 (0.0956,0.103)	0.188 (0.185,0.19)
All non-null hypotheses shared	0.0982 (0.0952,0.101)	0.195 (0.193,0.197)
Negative information	0.0595 (0.0587,0.0604)	0.302 (0.301,0.303)
Block correlation	0.0961 (0.0925,0.0997)	0.2 (0.194,0.206)
Equicorrelation	0.0987 (0.093,0.104)	0.191 (0.182,0.2)

See continuation on following page

Description	FDR(cFDR)	TDR(cFDR)
Reference	0.0955 (0.0926,0.0984)	0.208 (0.206,0.21)
No effects	0.0973 (0.0788,0.116)	
Weak effects	0.0814 (0.0695,0.0934)	0.00795 (0.00742,0.00849)
Large variance in effect sizes	0.0957 (0.0939,0.0975)	0.517 (0.514,0.519)
Larger n	0.0995 (0.0964,0.103)	0.189 (0.187,0.191)
Smaller n	0.0795 (0.0772,0.0818)	0.265 (0.263,0.268)
No non-null shared hypotheses	0.102 (0.0979,0.105)	0.178 (0.175,0.18)
All non-null hypotheses shared	0.0963 (0.0939,0.0987)	0.26 (0.258,0.263)
Negative information	0.0585 (0.0576,0.0593)	0.314 (0.312,0.315)
Block correlation	0.0974 (0.0939,0.101)	0.213 (0.207,0.219)
Equicorrelation	0.0897 (0.0843,0.095)	0.205 (0.196,0.215)

Description	FDR(oracle)	TDR(oracle)
Reference	0.0953 (0.0925,0.0982)	0.212 (0.21,0.214)
No effects	1 (1,1)	
Weak effects	0.0873 (0.0752,0.0994)	0.00904 (0.00846,0.00962)
Large variance in effect sizes	0.0945 (0.0927,0.0963)	0.527 (0.524,0.529)
Larger n	0.0997 (0.0966,0.103)	0.193 (0.191,0.195)
Smaller n	0.0787 (0.0765,0.0809)	0.269 (0.266,0.271)
No non-null shared hypotheses	0.0975 (0.094,0.101)	0.187 (0.185,0.19)
All non-null hypotheses shared	0.0805 (0.0783,0.0826)	0.309 (0.307,0.312)
Negative information	0.058 (0.0572,0.0588)	0.319 (0.318,0.32)
Block correlation	0.0933 (0.0901,0.0965)	0.217 (0.211,0.223)
Equicorrelation	0.0955 (0.0905,0.101)	0.209 (0.2,0.218)

Table 1: FDR and TDR of p-value, $\widehat{cFDR}^{n}$, and oracle cfdr (best possible procedure) using leave-one-out v-values (equation (25)) for a range of simulation parameters, controlling FDR at $\alpha = 0.1$. Cells show mean and 95% confidence interval. TDR is undefined if $n_1^p + n_1^{pq} = 0$.
