# Supplementary figures and images for: Accurate error control in high-dimensional association testing using conditional false discovery rates
Source: Biom J. Author manuscript; Available in PMC 2022 Feb 3. (PMC7612315; doi:10.1002/bimj.201900254)

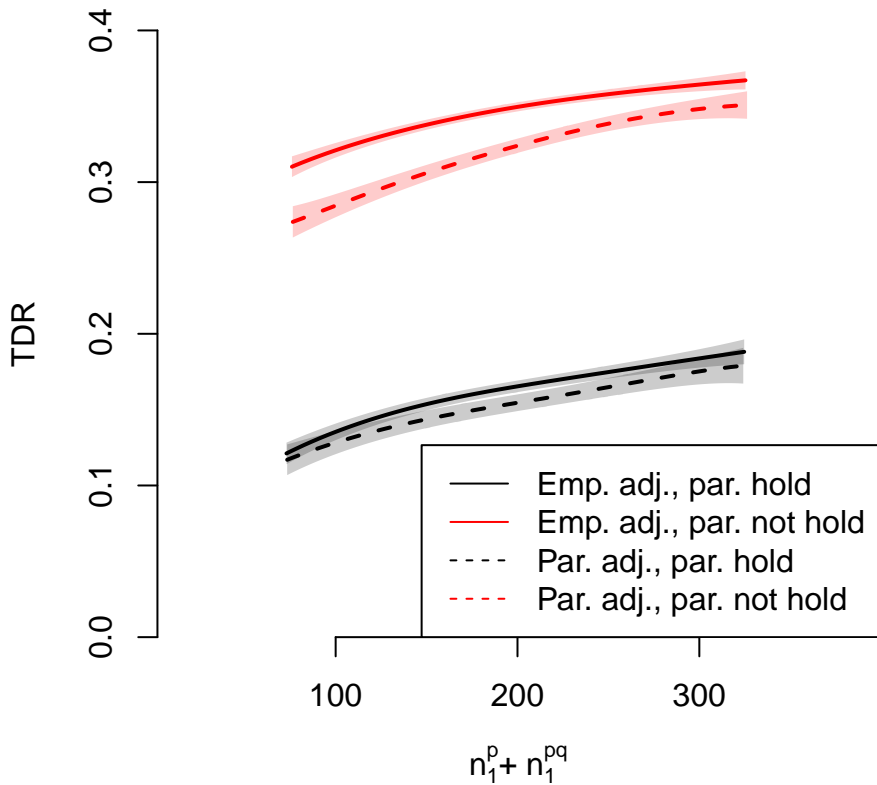

Supplement: Supplementary material 2 [file EMS140914-supplement-Supplementary_material_2.zip › cfdr_pipeline-master/outputs/adjustment_par_vs_emp.pdf]

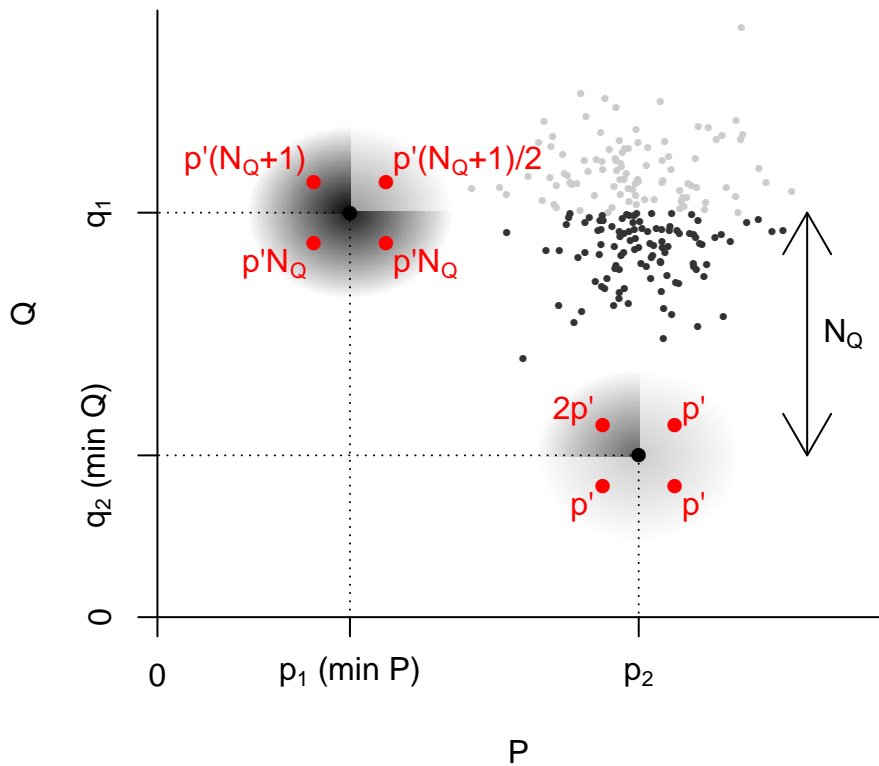

Supplement: Supplementary material 2 [file EMS140914-supplement-Supplementary_material_2.zip › cfdr_pipeline-master/outputs/chaoticity.pdf]

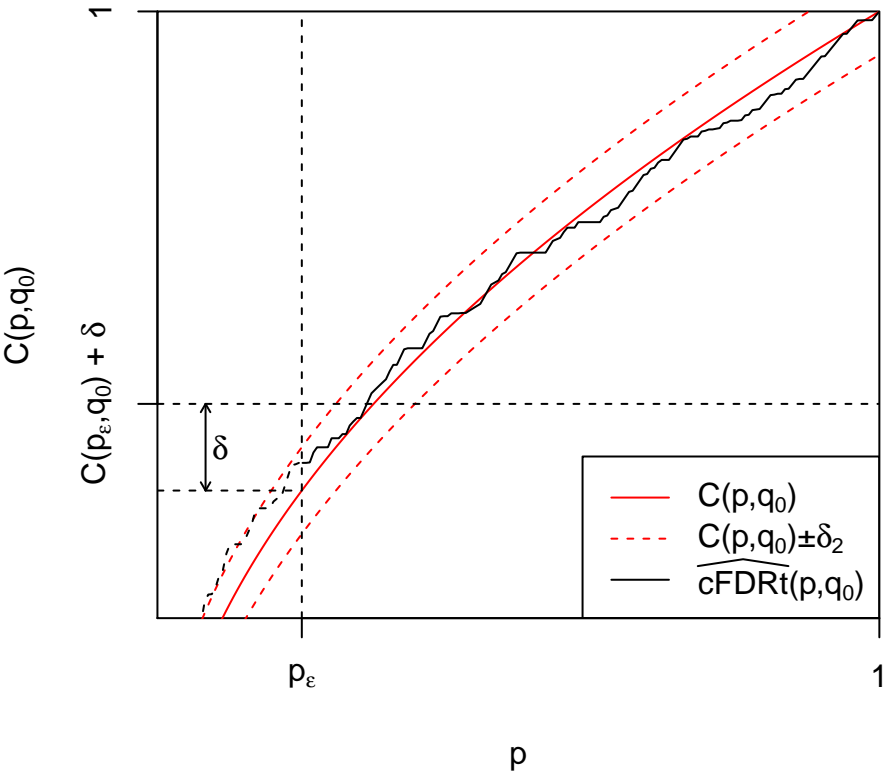

Supplement: Supplementary material 2 [file EMS140914-supplement-Supplementary_material_2.zip › cfdr_pipeline-master/outputs/convergence.pdf]

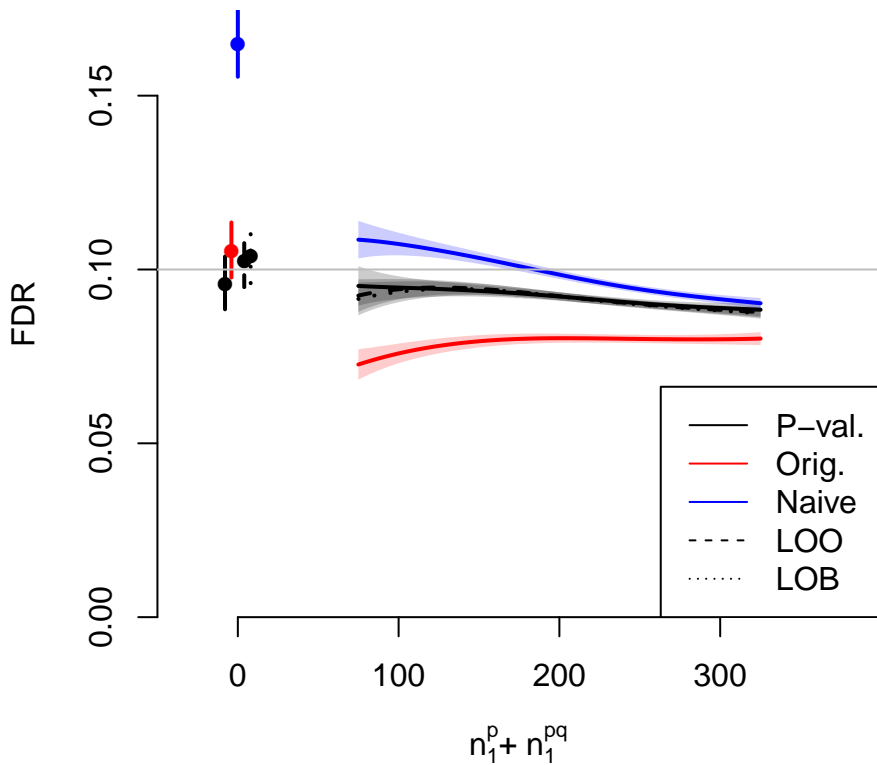

Supplement: Supplementary material 2 [file EMS140914-supplement-Supplementary_material_2.zip › cfdr_pipeline-master/outputs/fdr_control_alpha1_dist1.pdf]

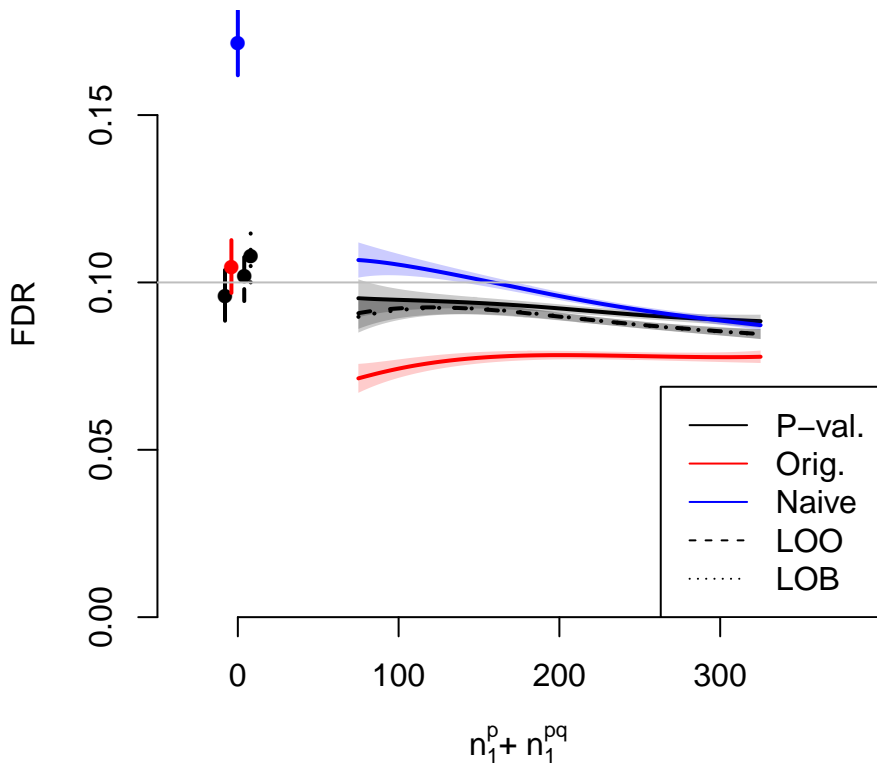

Supplement: Supplementary material 2 [file EMS140914-supplement-Supplementary_material_2.zip › cfdr_pipeline-master/outputs/fdr_control_alpha1_dist2.pdf]

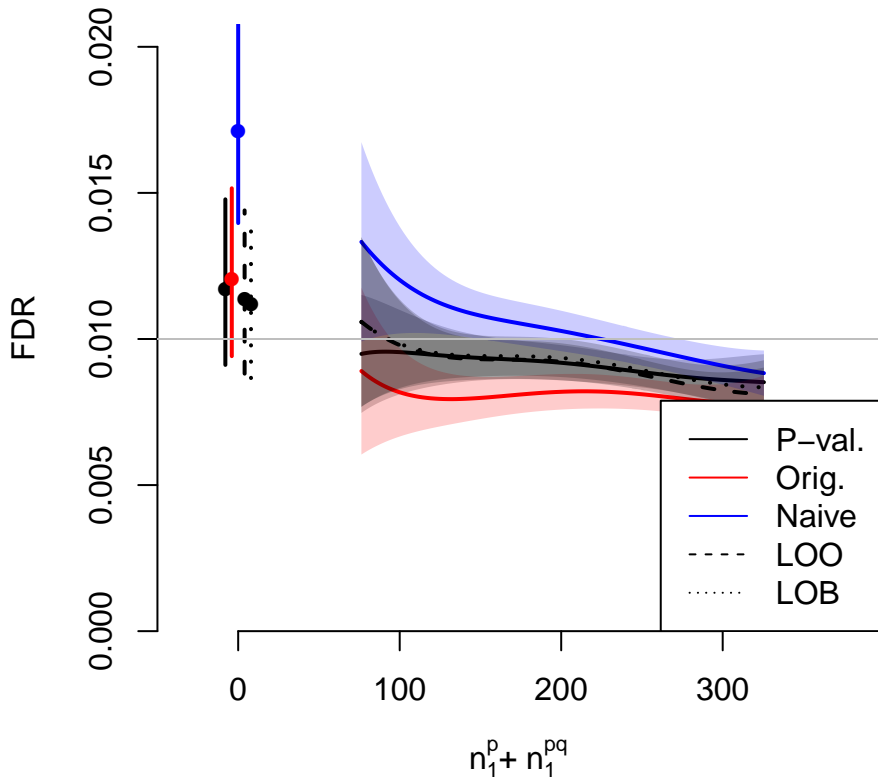

Supplement: Supplementary material 2 [file EMS140914-supplement-Supplementary_material_2.zip › cfdr_pipeline-master/outputs/fdr_control_alpha2_dist1.pdf]

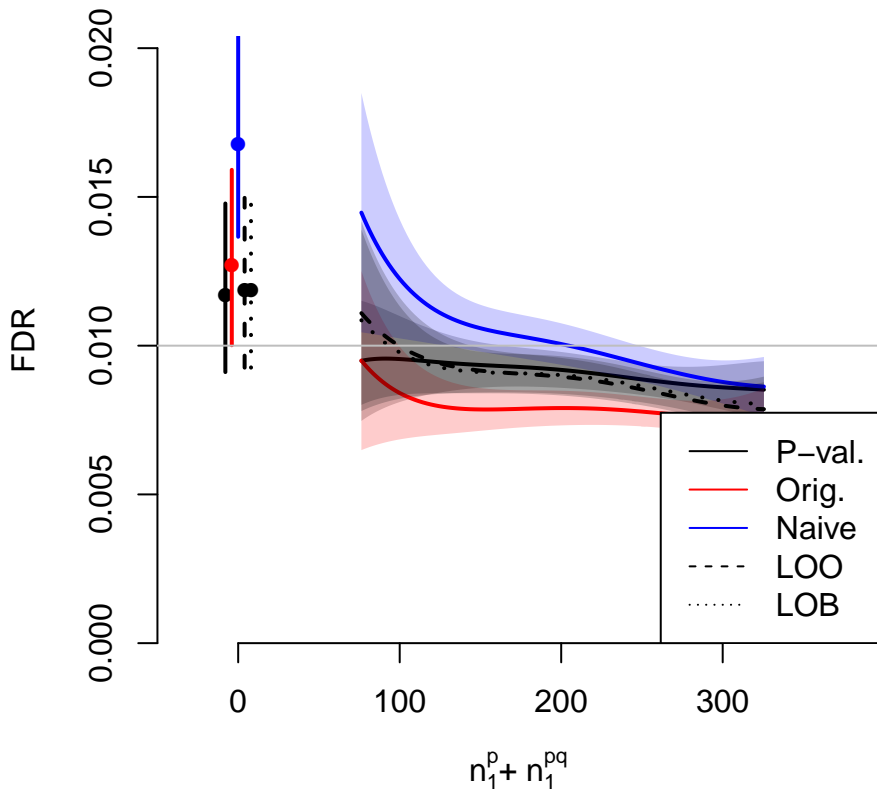

Supplement: Supplementary material 2 [file EMS140914-supplement-Supplementary_material_2.zip › cfdr_pipeline-master/outputs/fdr_control_alpha2_dist2.pdf]

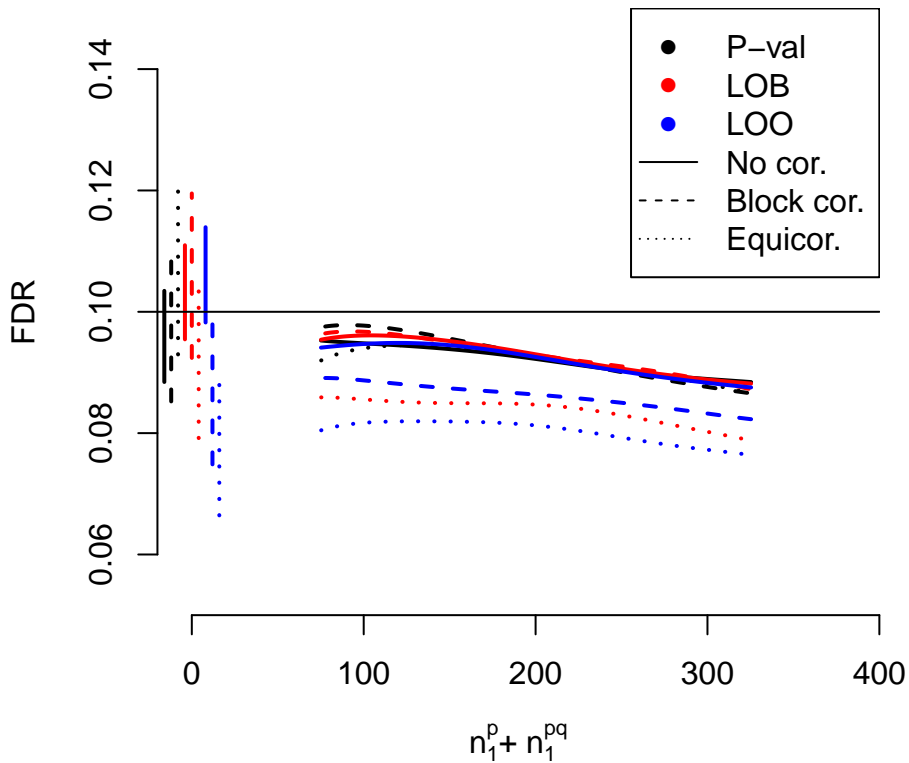

Supplement: Supplementary material 2 [file EMS140914-supplement-Supplementary_material_2.zip › cfdr_pipeline-master/outputs/fdr_cov_1.pdf]

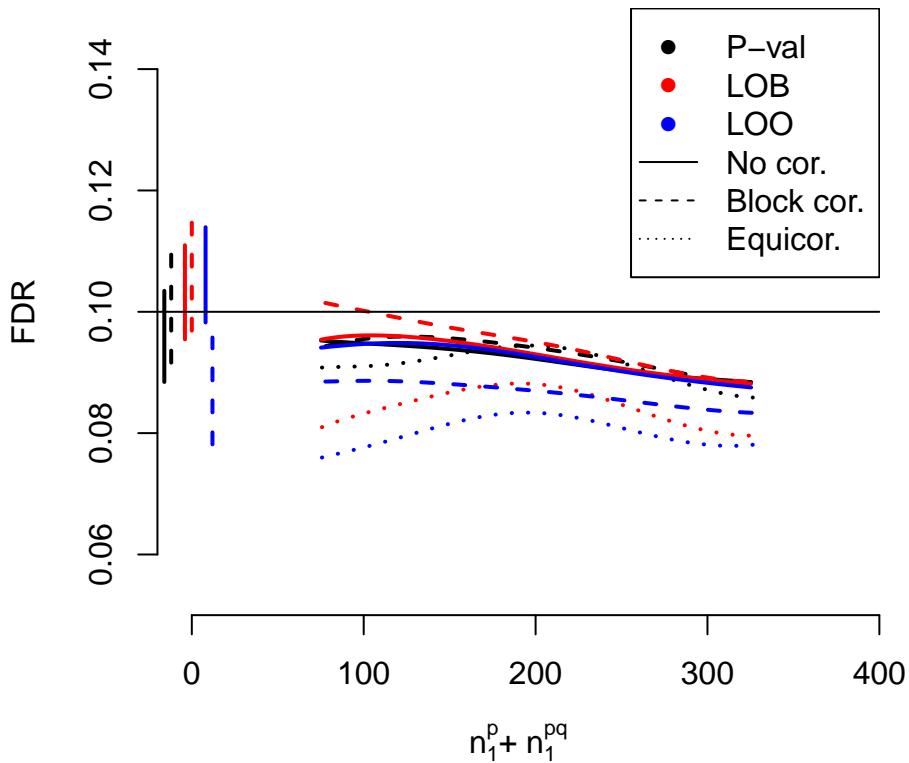

Supplement: Supplementary material 2 [file EMS140914-supplement-Supplementary_material_2.zip › cfdr_pipeline-master/outputs/fdr_cov_2.pdf]

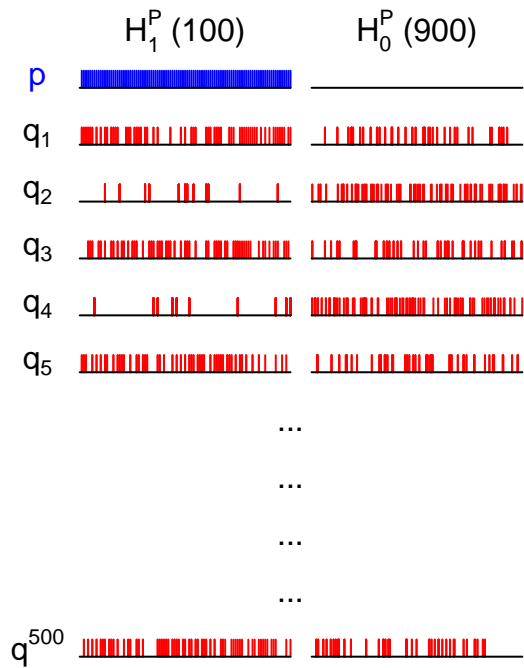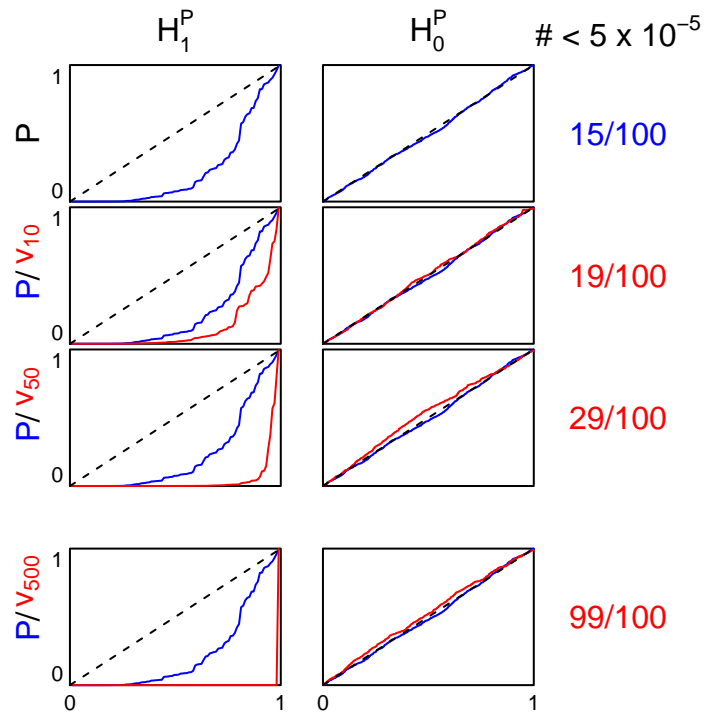

Supplement: Supplementary material 2 [file EMS140914-supplement-Supplementary_material_2.zip › cfdr_pipeline-master/outputs/fig_iterated.pdf]

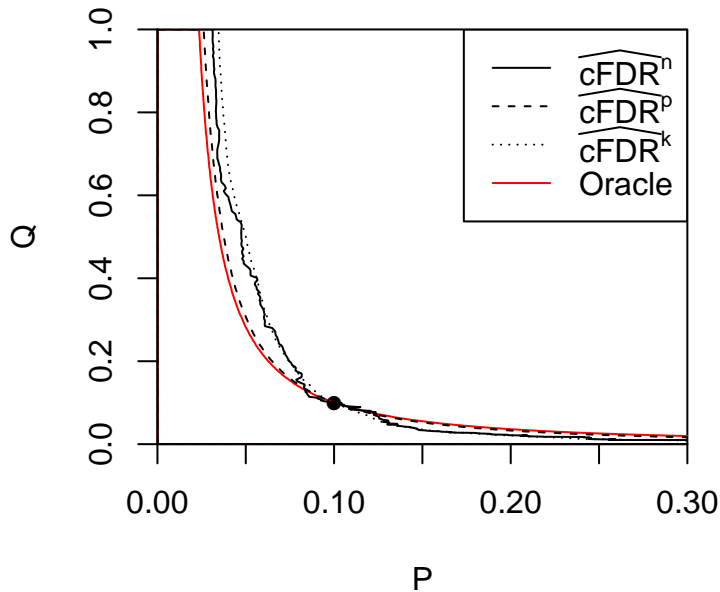

Supplement: Supplementary material 2 [file EMS140914-supplement-Supplementary_material_2.zip › cfdr_pipeline-master/outputs/lcomp_10000_cdf.pdf]

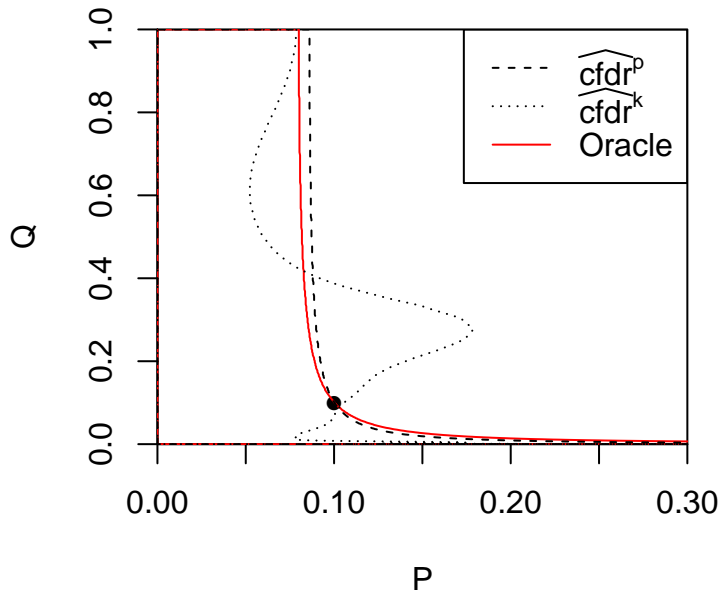

Supplement: Supplementary material 2 [file EMS140914-supplement-Supplementary_material_2.zip › cfdr_pipeline-master/outputs/lcomp_10000_pdf.pdf]

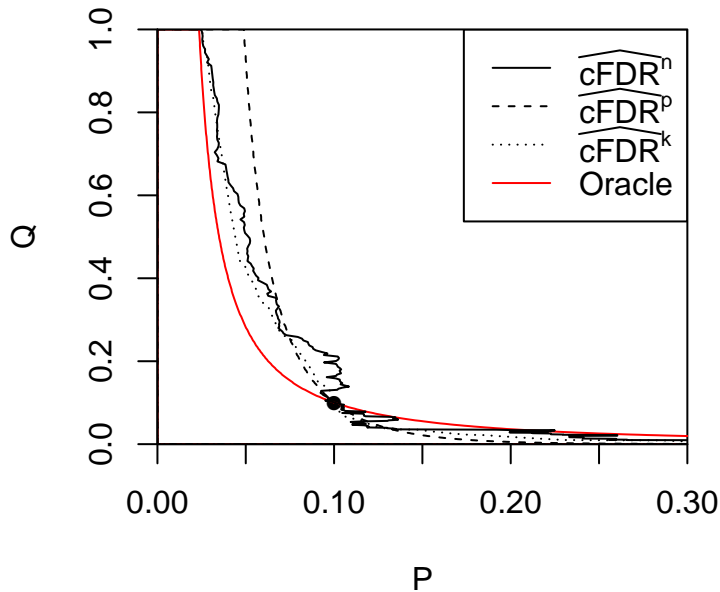

Supplement: Supplementary material 2 [file EMS140914-supplement-Supplementary_material_2.zip › cfdr_pipeline-master/outputs/lcomp_1000_cdf.pdf]

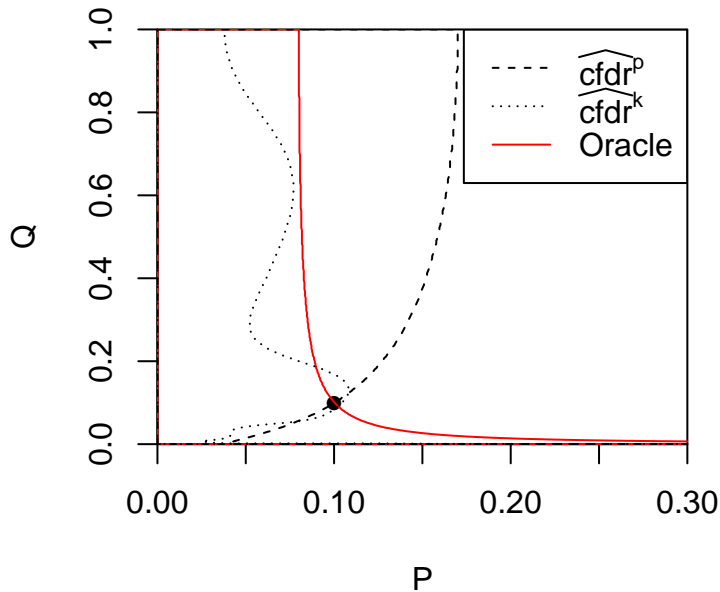

Supplement: Supplementary material 2 [file EMS140914-supplement-Supplementary_material_2.zip › cfdr_pipeline-master/outputs/lcomp_1000_pdf.pdf]

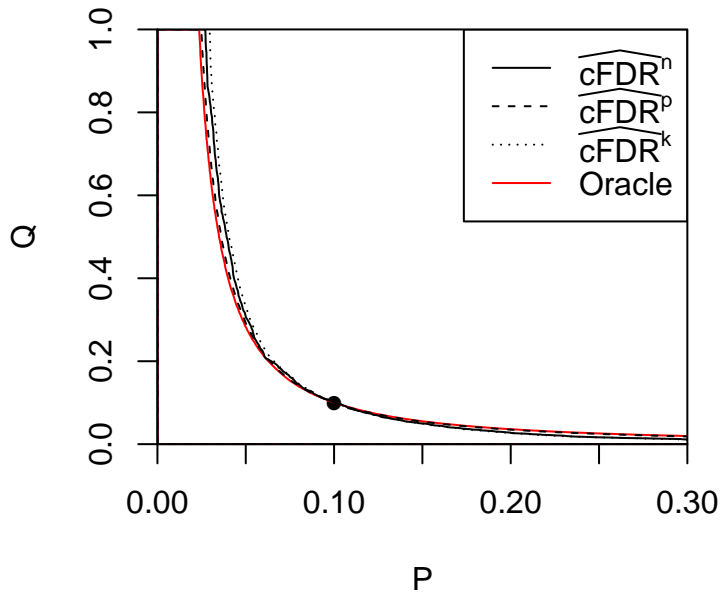

Supplement: Supplementary material 2 [file EMS140914-supplement-Supplementary_material_2.zip › cfdr_pipeline-master/outputs/lcomp_1e+05_cdf.pdf]

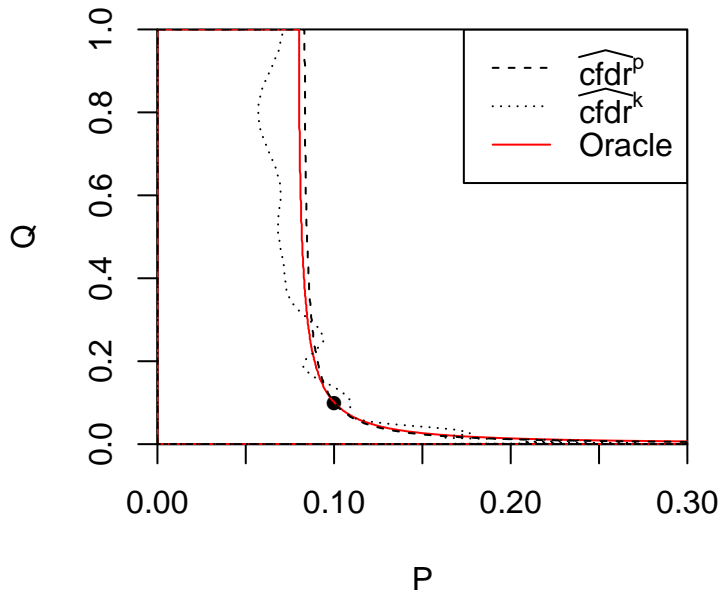

Supplement: Supplementary material 2 [file EMS140914-supplement-Supplementary_material_2.zip › cfdr_pipeline-master/outputs/lcomp_1e+05_pdf.pdf]

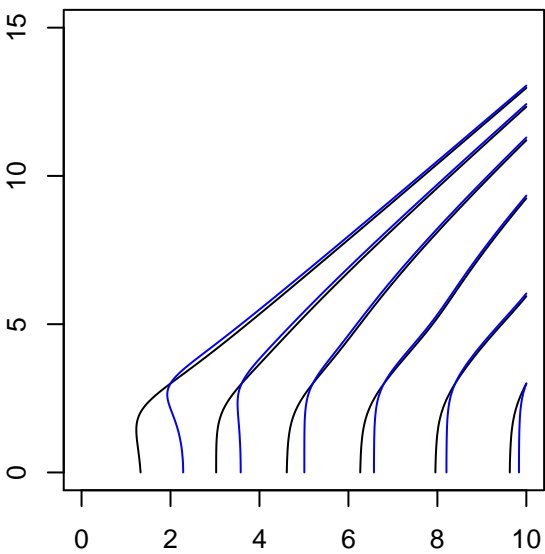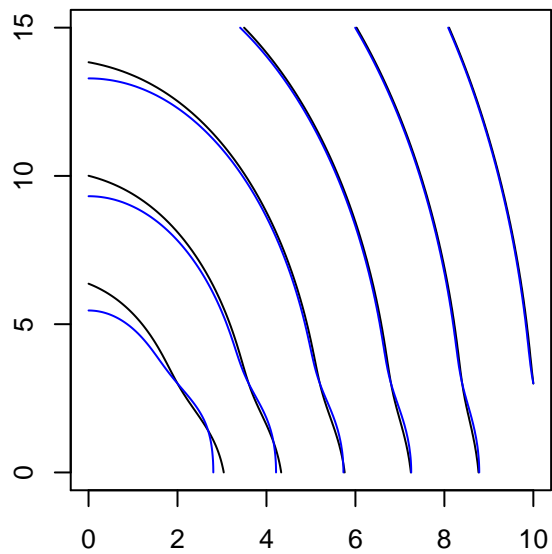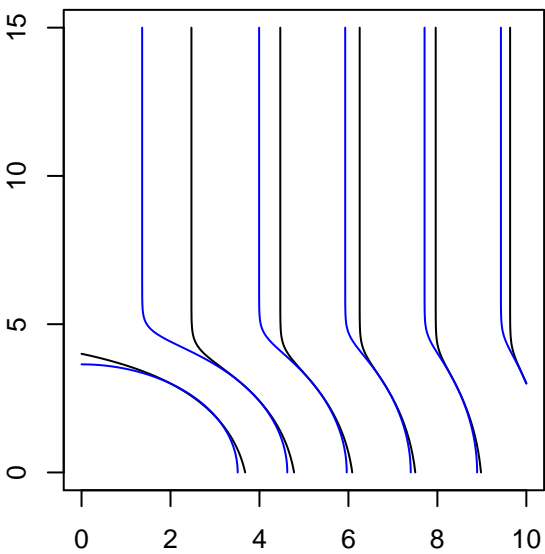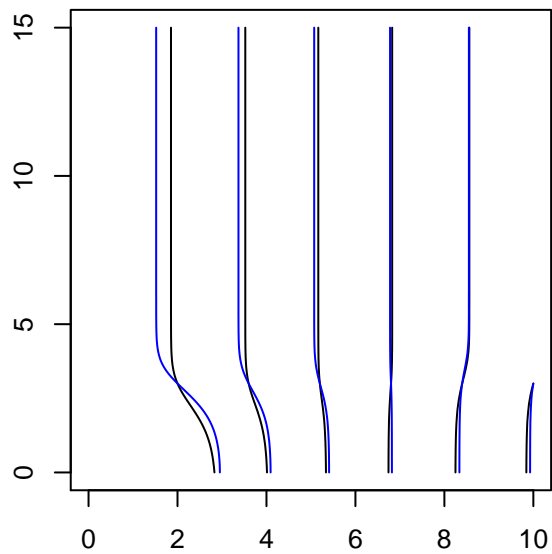

Supplement: Supplementary material 2 [file EMS140914-supplement-Supplementary_material_2.zip › cfdr_pipeline-master/outputs/lconvergence.pdf]

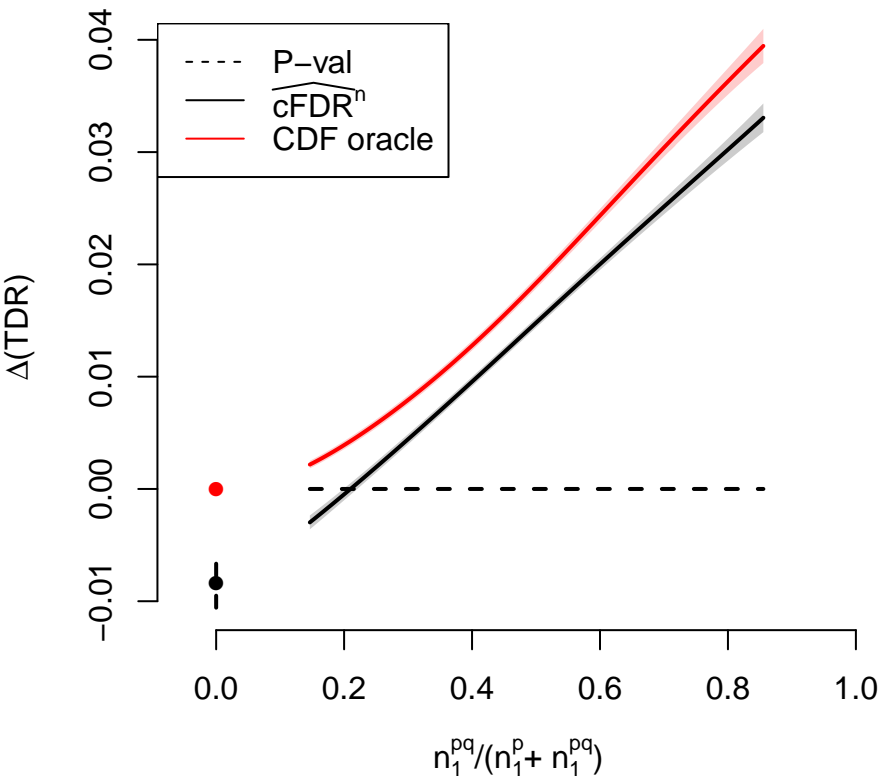

Supplement: Supplementary material 2 [file EMS140914-supplement-Supplementary_material_2.zip › cfdr_pipeline-master/outputs/min_percentage_alpha_high.pdf]

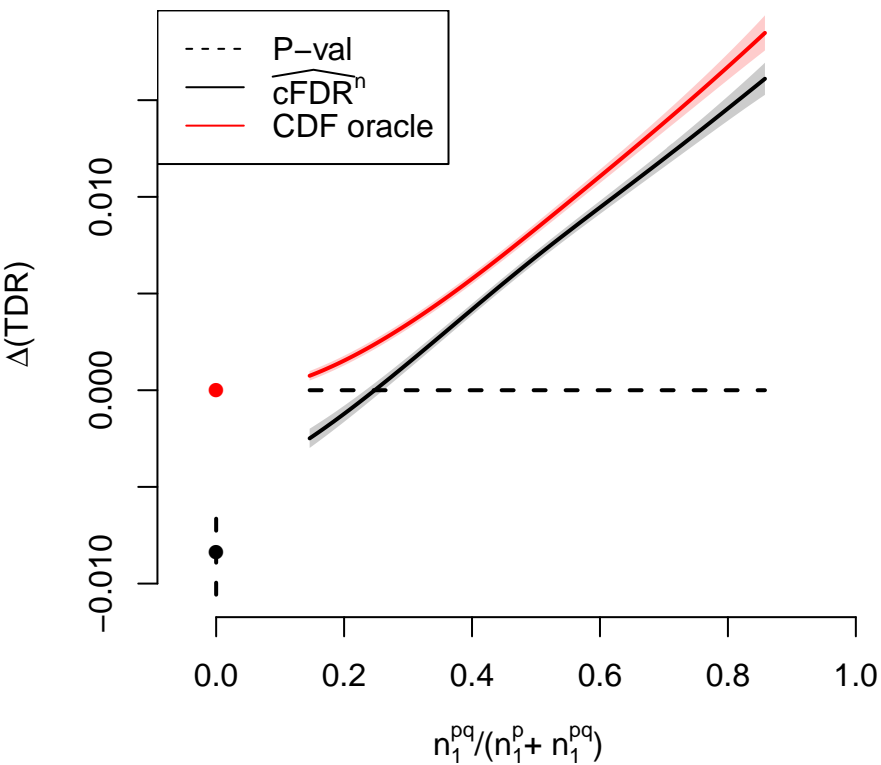

Supplement: Supplementary material 2 [file EMS140914-supplement-Supplementary_material_2.zip › cfdr_pipeline-master/outputs/min_percentage_alpha_low.pdf]

$H^1_P$ 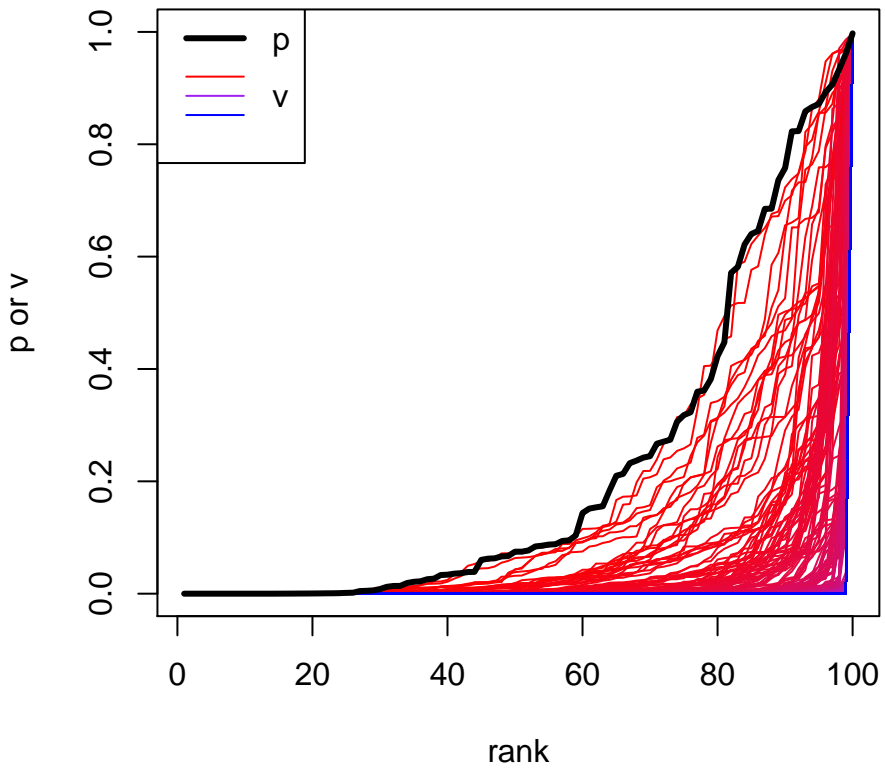 $H^0_P$ 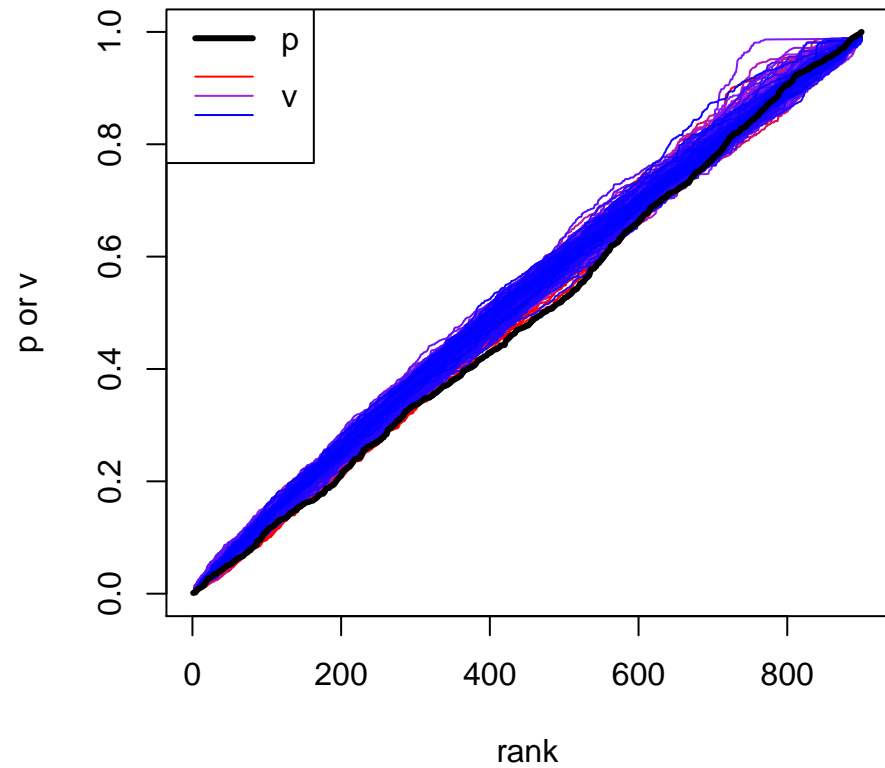

Supplement: Supplementary material 2 [file EMS140914-supplement-Supplementary_material_2.zip › cfdr_pipeline-master/outputs/mix_condition.pdf]

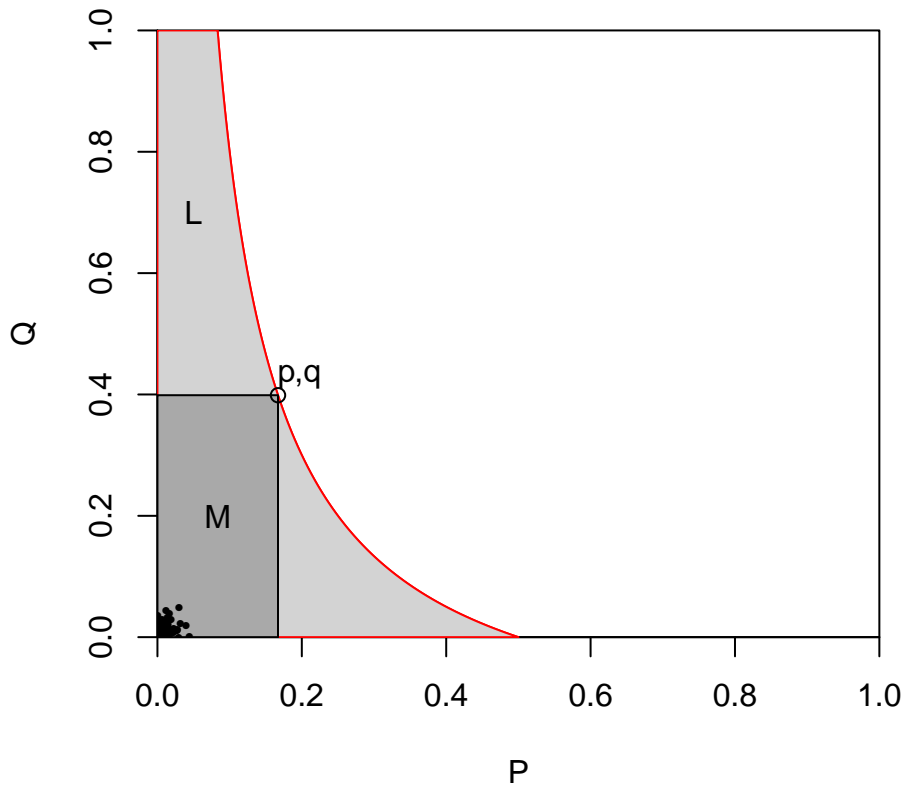

Supplement: Supplementary material 2 [file EMS140914-supplement-Supplementary_material_2.zip › cfdr_pipeline-master/outputs/ml.pdf]

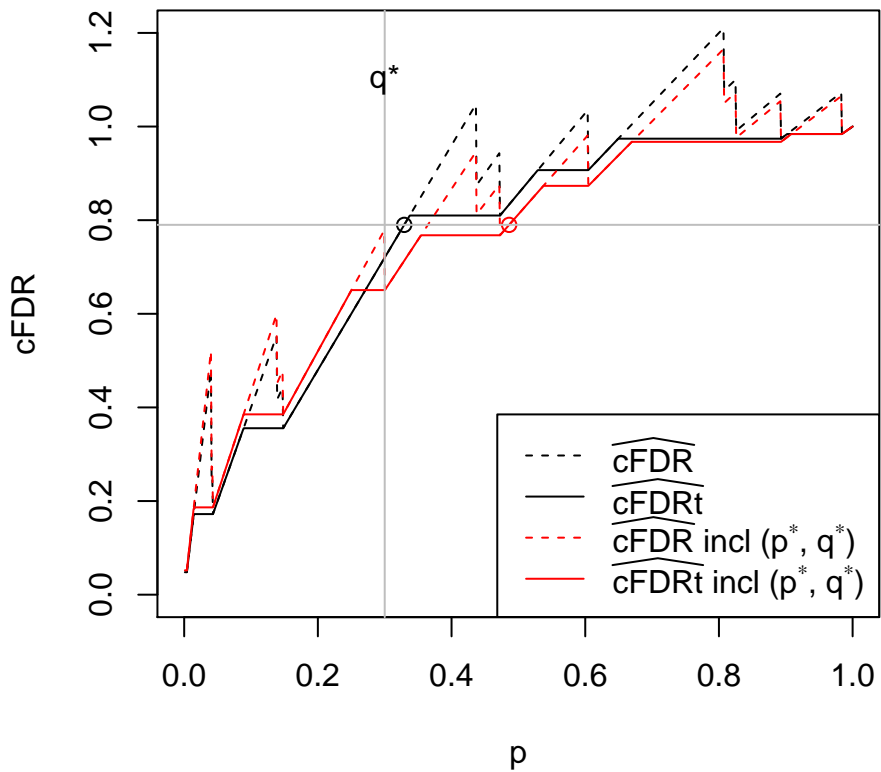

Supplement: Supplementary material 2 [file EMS140914-supplement-Supplementary_material_2.zip › cfdr_pipeline-master/outputs/onepoint_demo.pdf]

Change

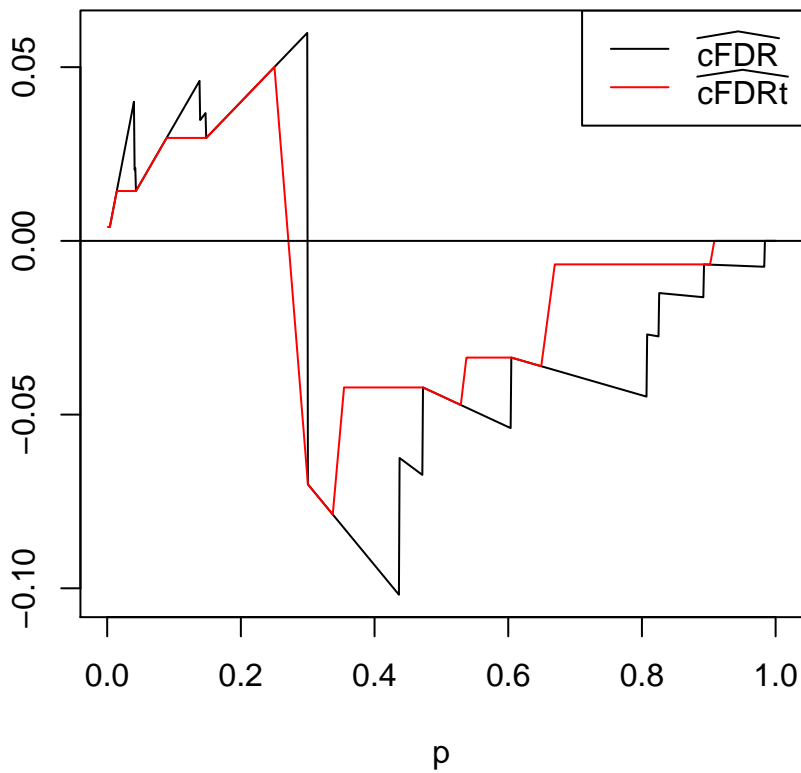

Supplement: Supplementary material 2 [file EMS140914-supplement-Supplementary_material_2.zip › cfdr_pipeline-master/outputs/onepoint_diff.pdf]

TDR

0.24 0.26 0.28 0.30

100

200

300

$n_1^p + n_1^{pq}$

---

P-val.

---

$\widehat{\text{cFDR}}^n$

—

$\widehat{\text{cFDR}}$

---

Orig.

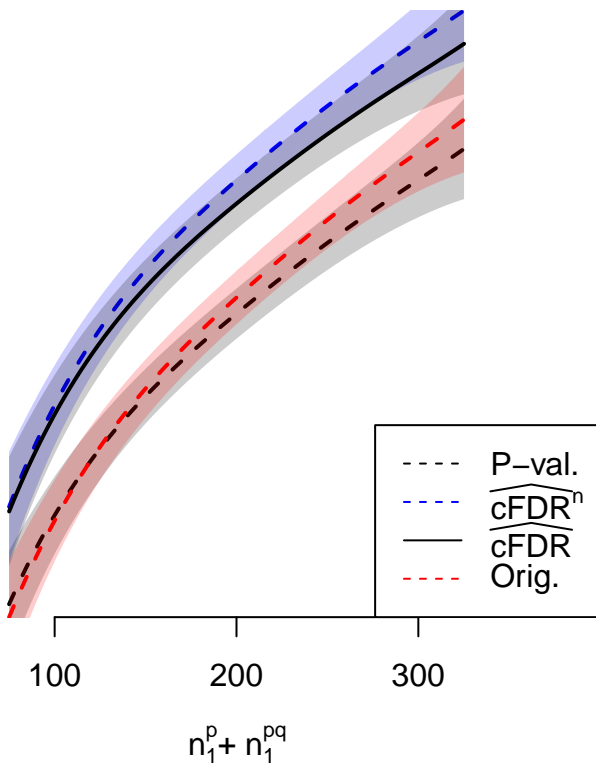

Supplement: Supplementary material 2 [file EMS140914-supplement-Supplementary_material_2.zip › cfdr_pipeline-master/outputs/power1_alpha1.pdf]

TDR

0.19  
0.20  
0.21  
0.22

100

200

300

$n_1^p + n_1^{pq}$

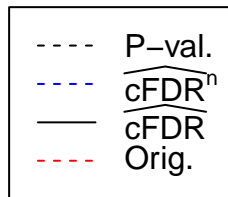

Supplement: Supplementary material 2 [file EMS140914-supplement-Supplementary_material_2.zip › cfdr_pipeline-master/outputs/power1_alpha2.pdf]

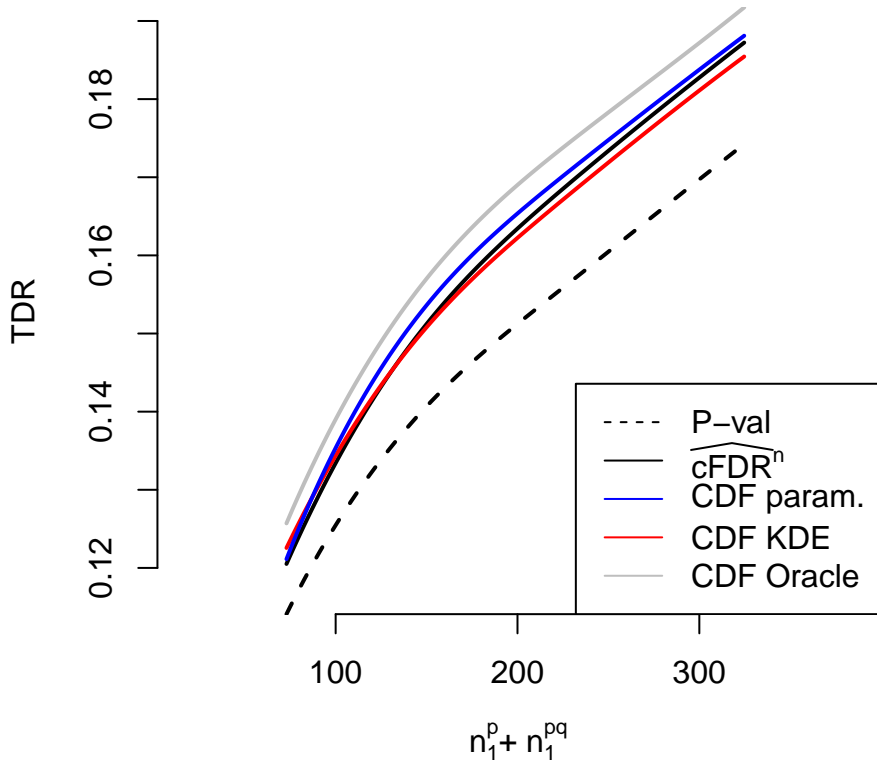

Supplement: Supplementary material 2 [file EMS140914-supplement-Supplementary_material_2.zip › cfdr_pipeline-master/outputs/power2_dist1_alpha1.pdf]

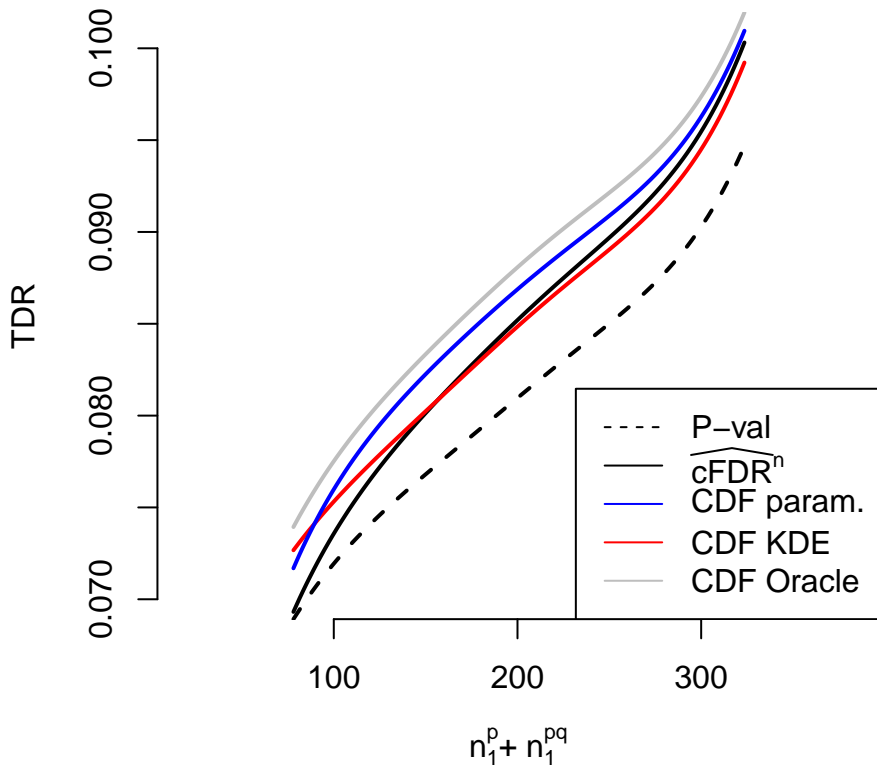

Supplement: Supplementary material 2 [file EMS140914-supplement-Supplementary_material_2.zip › cfdr_pipeline-master/outputs/power2_dist1_alpha2.pdf]

TDR

0.31  
0.33  
0.35  
0.37

100

200

300

$n_1^p + n_1^{pq}$

- P-val
- $\widehat{\text{cFDR}}^n$
- CDF param.
- CDF KDE
- CDF Oracle

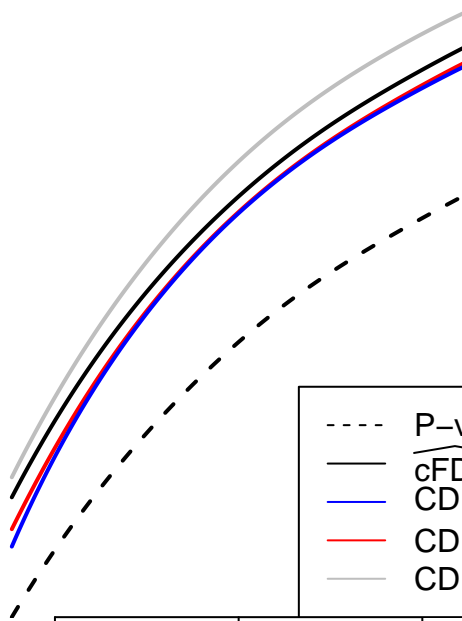

Supplement: Supplementary material 2 [file EMS140914-supplement-Supplementary_material_2.zip › cfdr_pipeline-master/outputs/power2_dist2_alpha1.pdf]

TDR

0.25  
0.26  
0.27  
0.28

100

200

300

$n_1^p + n_1^{pq}$

- P-val
- $\widehat{\text{cFDR}}^n$
- CDF param.
- CDF KDE
- CDF Oracle

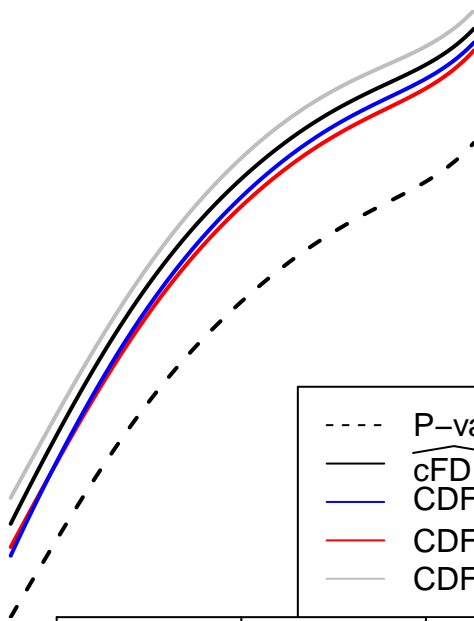

Supplement: Supplementary material 2 [file EMS140914-supplement-Supplementary_material_2.zip › cfdr_pipeline-master/outputs/power2_dist2_alpha2.pdf]

Power

0.10 0.12 0.14 0.16 0.18

100

200

300

$n_1^p + n_1^{pq}$

- P-val
- $\widehat{\text{cFDR}}^n$
- CDF oracle
- ... PDF, param.
- ... PDF, KDE
- ... PDF oracle

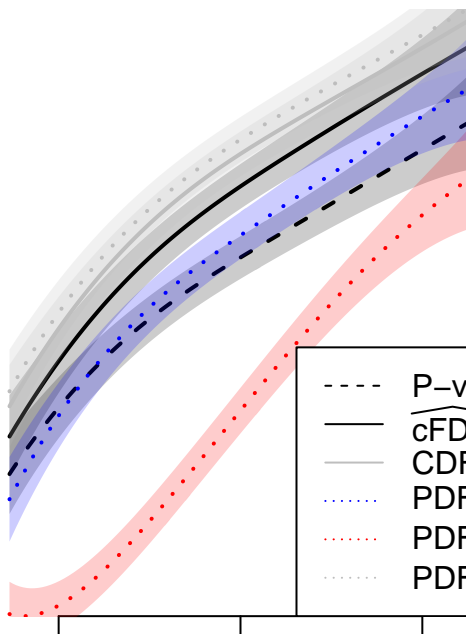

Supplement: Supplementary material 2 [file EMS140914-supplement-Supplementary_material_2.zip › cfdr_pipeline-master/outputs/power3_alpha1_dist1.pdf]

Power

0.35  
0.30  
0.25  
0.20

100

200

300

$n_1^p + n_1^{pq}$

- P-val
- $\widehat{\text{cFDR}}^n$
- CDF oracle
- ... PDF, param.
- ... PDF, KDE
- ... PDF oracle

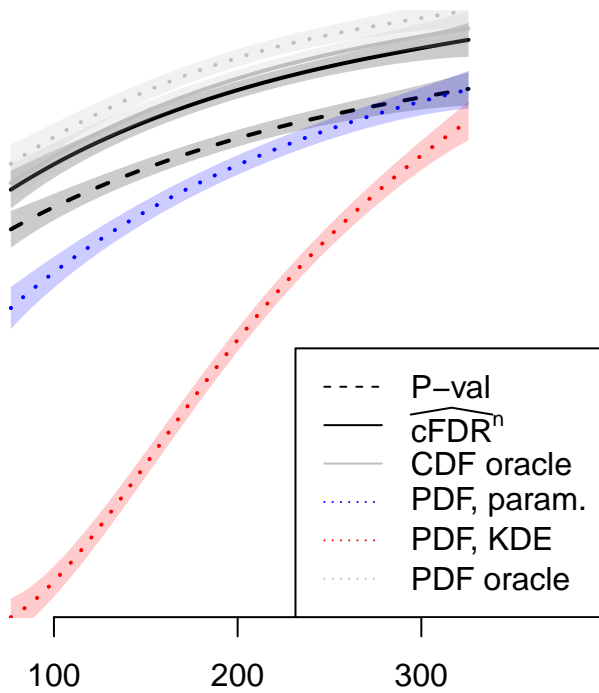

Supplement: Supplementary material 2 [file EMS140914-supplement-Supplementary_material_2.zip › cfdr_pipeline-master/outputs/power3_alpha1_dist2.pdf]

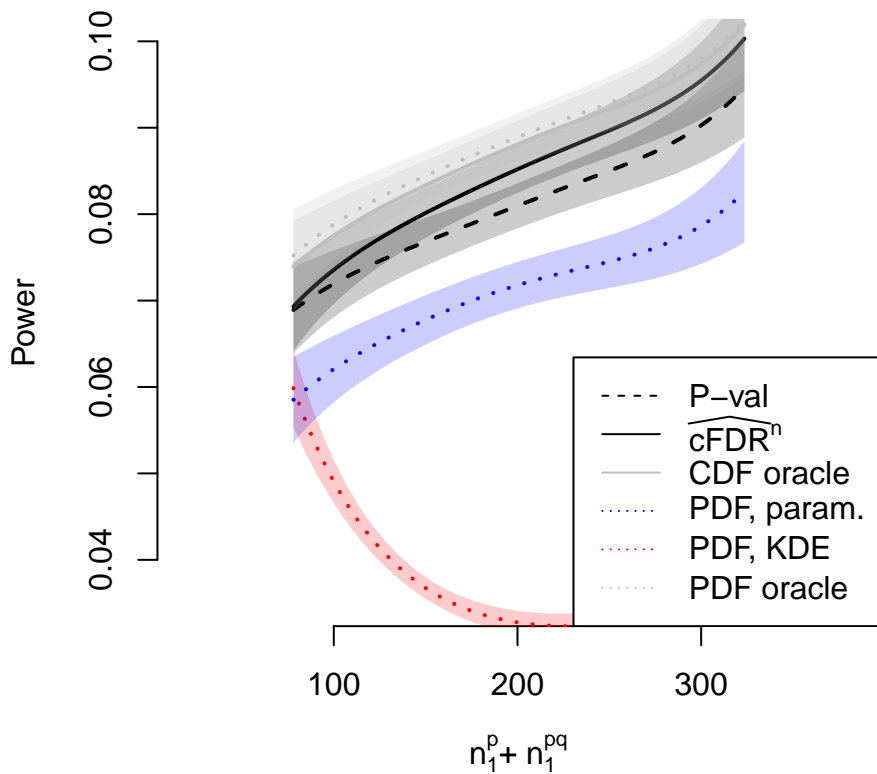

Supplement: Supplementary material 2 [file EMS140914-supplement-Supplementary_material_2.zip › cfdr_pipeline-master/outputs/power3_alpha2_dist1.pdf]

Power

0.10  
0.15  
0.20  
0.25

100

200

300

$n_1^p + n_1^{pq}$

- P-val
- $\widehat{\text{cFDR}}^n$
- CDF oracle
- ... PDF, param.
- ... PDF, KDE
- ... PDF oracle

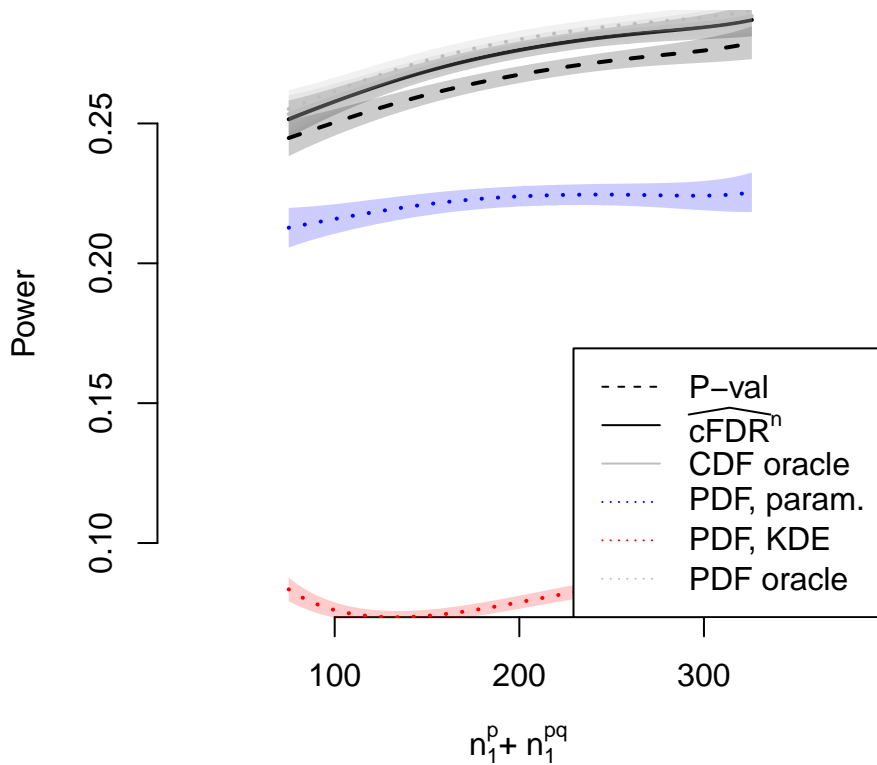

Supplement: Supplementary material 2 [file EMS140914-supplement-Supplementary_material_2.zip › cfdr_pipeline-master/outputs/power3_alpha2_dist2.pdf]

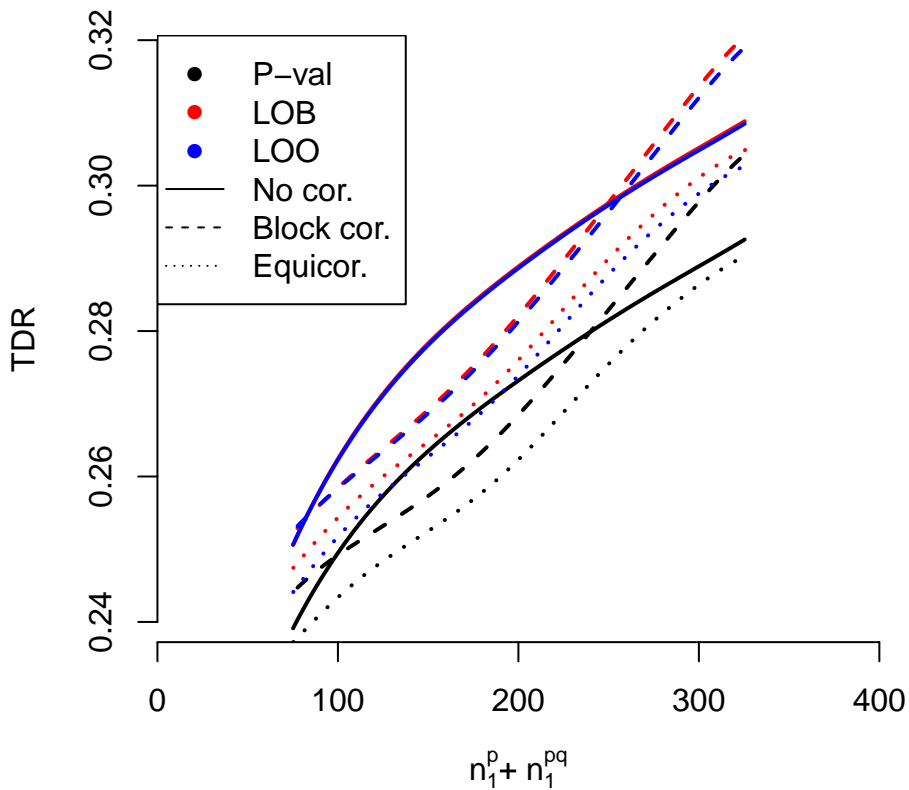

Supplement: Supplementary material 2 [file EMS140914-supplement-Supplementary_material_2.zip › cfdr_pipeline-master/outputs/tdr_cov_1.pdf]

TDR

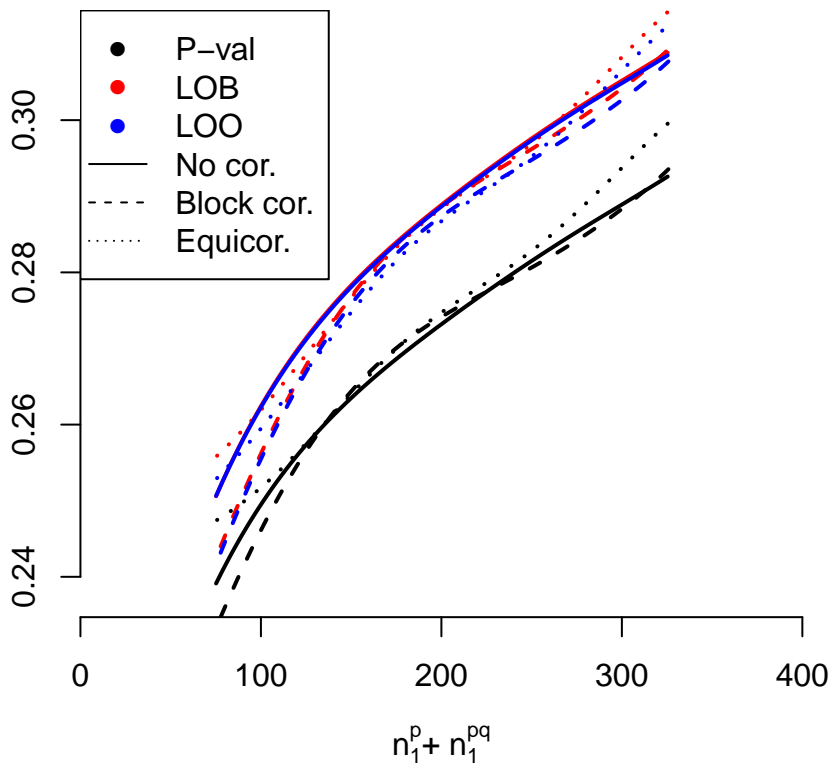

Supplement: Supplementary material 2 [file EMS140914-supplement-Supplementary_material_2.zip › cfdr_pipeline-master/outputs/tdr_cov_2.pdf]

# C. Rej. reg: cFDR-based

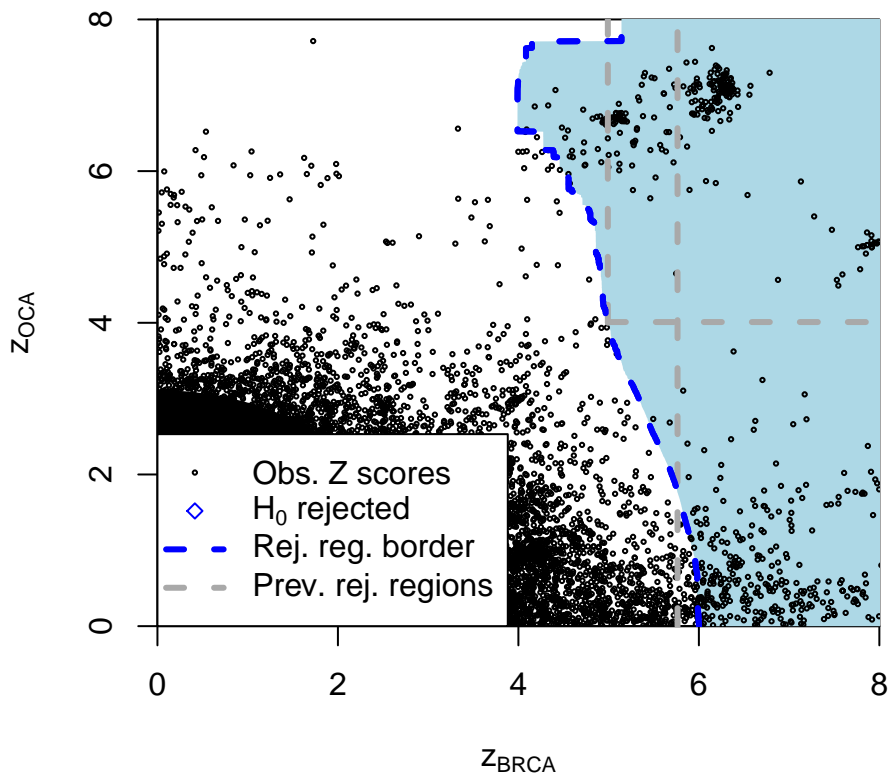

Supplement: Supplementary material 2 [file EMS140914-supplement-Supplementary_material_2.zip › cfdr_pipeline-master/outputs/twas_regions_brca.pdf]

A. Rej. reg. from B-H on  $p_{\text{BRCA}}$  only

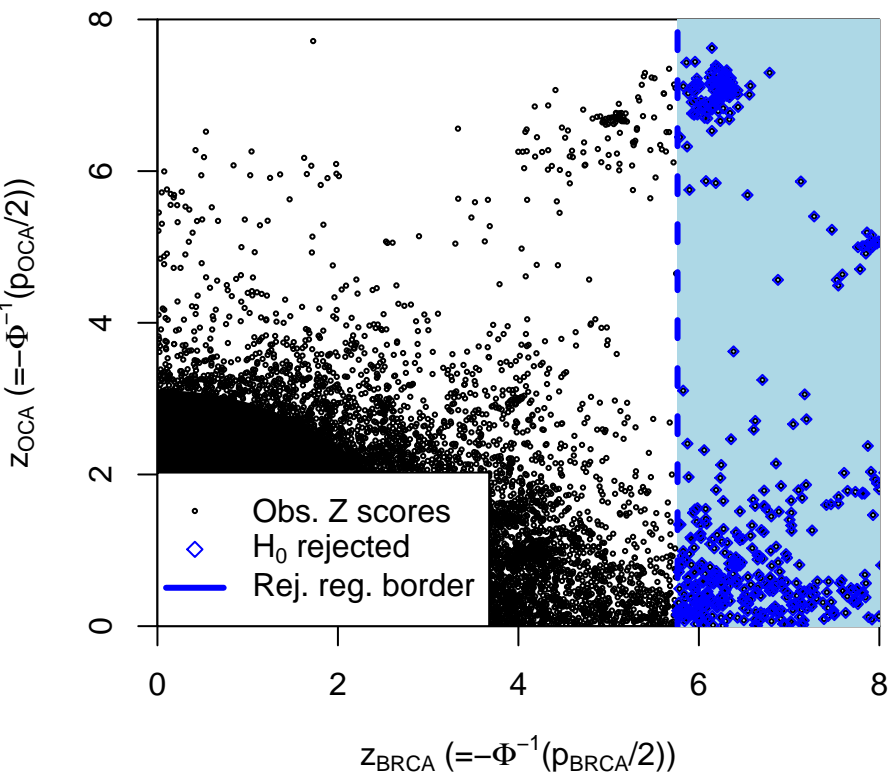

Supplement: Supplementary material 2 [file EMS140914-supplement-Supplementary_material_2.zip › cfdr_pipeline-master/outputs/twas_regions_brca_bh.pdf]

B. Rej. reg: B-H on  $p_{\text{BRCA}}|p_{\text{OCA}} < 10^{-4}$

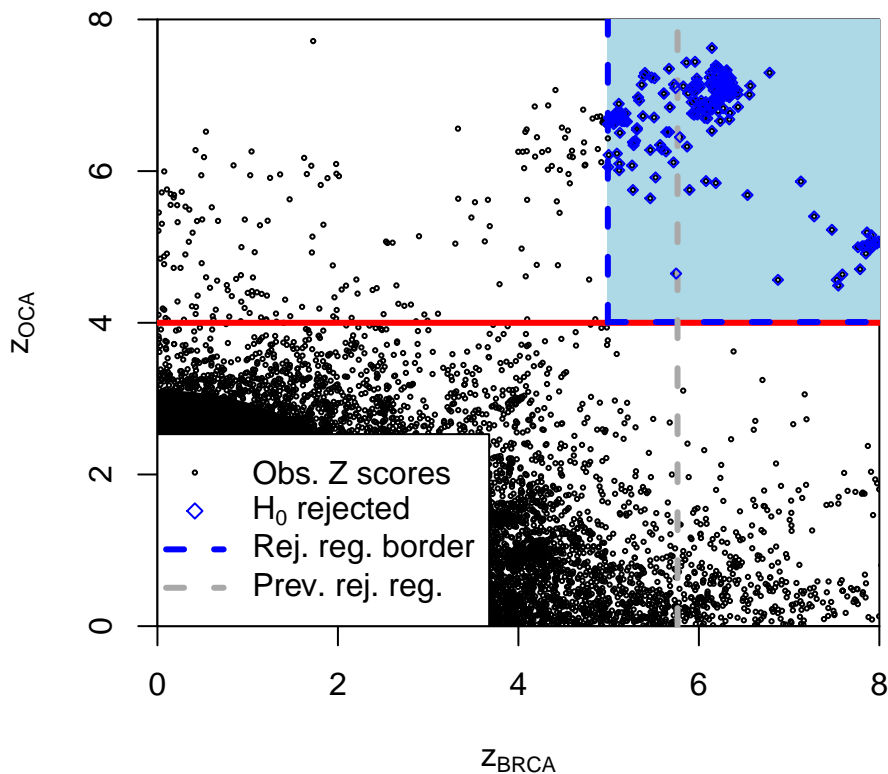

Supplement: Supplementary material 2 [file EMS140914-supplement-Supplementary_material_2.zip › cfdr_pipeline-master/outputs/twas_regions_brca_if.pdf]

### D. L-reg. , p-val scale

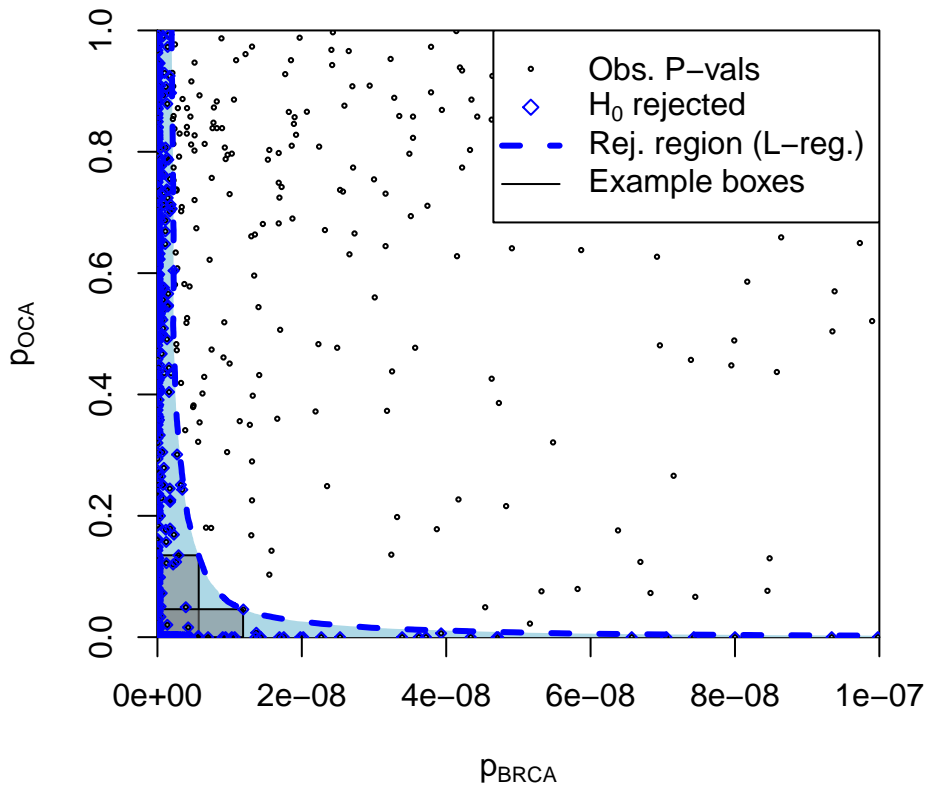

Supplement: Supplementary material 2 [file EMS140914-supplement-Supplementary_material_2.zip › cfdr_pipeline-master/outputs/twas_regions_brca_pval.pdf]

# Rej. reg: cFDR-based, OCA|BRCA

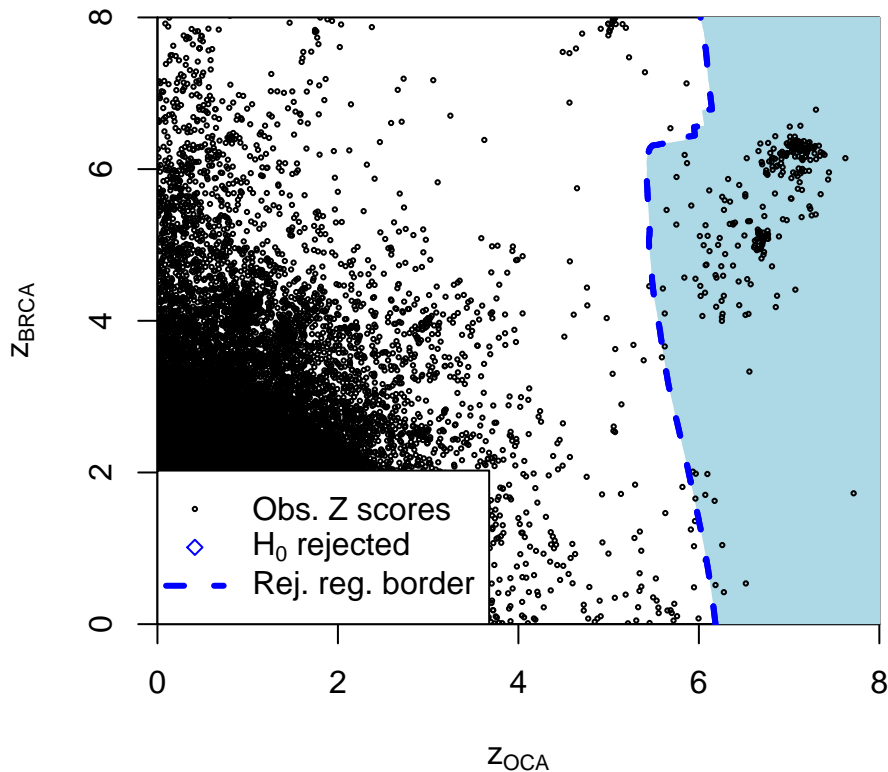

Supplement: Supplementary material 2 [file EMS140914-supplement-Supplementary_material_2.zip › cfdr_pipeline-master/outputs/twas_regions_oca.pdf]
