# Supplementary material for: Accurate error control in high-dimensional association testing using conditional false discovery rates
Source: Biom J. Author manuscript; Available in PMC 2022 Feb 3. (PMC7612315; doi:10.1002/bimj.201900254)
Supplement: Appendix [file EMS140914-supplement-Appendix.pdf]

SUPPORTING INFORMATION

Additional supporting information may be found online in the Supporting Information section at the end of the article.

How to cite this article: Liley J, Wallace C. Accurate error control in high-dimensional association testing using conditional false discovery rates. *Biometrical Journal*. 2021;*63*:1096–1130. https://doi.org/10.1002/bimj.201900254

APPENDIX

A.1 | Optimal procedure

In this section, we show the following result. This is not original; it is shown in various forms in (at least) Alishahi et al. (2016), Du et al. (2014) and Lei and Fithian (2018).

Theorem A.1. *Let f_0 and f_1 be positive Lesbegue-integrable functions of (p, q) on some region Ω. Suppose a Lesbegue-measurable region R_0 satisfies:*

1. $R_0 = \{(p, q) : f_0(p, q)/f_1(p, q) \leq k, (p, q) \in \Omega\}$
2. $\int_{R_0} f_0(p, q)dpdq = \alpha$
3. $\int_{R_0} f_1(p, q)dpdq = 1 - \beta.$

Then no Lesbegue-measurable region $R \subset \Omega$ satisfies both

$$\int_R f_0(p, q)dpdq = \alpha \tag{A.1}$$

and

$$\int_R f_1(p, q)dpdq > 1 - \beta. \tag{A.2}$$

Proof. Suppose such a region existed. Then given condition (A.1), we must have $f_0(p, q)/f_1(p, q) > k$ in $R \setminus R_0$, and since the integral of f_0 over R is equal to its integral over R_0,

$$\int_{R \setminus R_0} f_0(p, q)dpdq = \int_{R_0 \setminus R} f_0(p, q)dpdq = \alpha - \int_{R_0 \cap R} f_0(p, q)dpdq. \tag{A.3}$$

Hence

$$\int_R f_1(p, q)dpdq = \int_{R \setminus R_0} f_1(p, q)dpdq + \int_{R \cap R_0} f_1(p, q)dpdq$$

$$\leq k \int_{R\backslash R_0} f_0(p,q)dpdq + \int_{R\cap R_0} f_1(p,q)dpdq$$

$$= k \int_{R_0\backslash R} f_0(p,q)dpdq + \int_{R\cap R_0} f_1(p,q)dpdq$$

$$\leq \int_{R_0\backslash R} f_1(p,q)dpdq + \int_{R\cap R_0} f_1(p,q)dpdq$$

$$= \int_{R_0} f_1(p,q)dpdq = 1-\beta \tag{A.4}$$

a contradiction of (A.2). Regions $R \neq R_0$ can satisfy (A.1) and

$$\int_R f_1(p,q)dpdq = 1-\beta \tag{A.5}$$

if and only if $R_0 \setminus R$ and $R \setminus R_0$ have Lesbegue measure 0. □

Corollary A.2. *If $f_0(p,q) = f(P=p, Q=q|H_0^P)$ and $f_1(p,q) = f(P=p,Q=q|H_1^P)$ (where H_1^P is the alternative) then amongst all rejection regions R with fixed type-1 error rate $\alpha = \int_R f_0(p,q)dpdq$, power is maximised on a region inside a contour of f_0/f_1, if such a region exists.*

Denoting $f(p,q) = \pi_0 f_0(p,q) + (1-\pi_0)f_1(p,q)$, it is clear that a contour of f_0/f_1 is also a contour of f_0/f and of $Pr(H_0^p|P=p,Q=q)$, so an optimal rejection region is given by

$$\left\{(p,q) : Pr(H_0^p|P=p,Q=q) \leq k_\alpha\right\} \tag{A.6}$$

for some k_α.

A.2 | Failure of FDR control with $\widehat{cFDR} < \alpha$

As described in Section 2, rejection procedure (9) is similar to the B-H procedure, and it may be naively thought that it also controls the FDR at α. This is not the case, and indeed the FDR of such a procedure (and the corresponding procedure with $\widehat{cFDR}^n$) may exceed α by an arbitrary factor depending on α and π_0.

This is most easily seen by considering the extreme case in which

$$P, Q|H_0^p \sim U(0,1)^2 \tag{A.7}$$

$$P, Q|H_1^p \sim (0,0), \tag{A.8}$$

where $\pi_0 = Pr(H_0^p)$ as usual. In this case we show:

Theorem A.3. *Under the above distribution of P, Q, as $n \to \infty$, the FDR α_{TRUE} of rejection procedure (9) for $\widehat{cFDR}$ satisfies*

$$\frac{\alpha_{TRUE}(1-\alpha)}{\alpha(1-\alpha_{TRUE})} = OR(\alpha_{TRUE}, \alpha) \to \log\left(\frac{1-\alpha\pi_0}{1-\pi_0}\right) \tag{A.9}$$

and the corresponding procedure for $\widehat{cFDR}^n$ satisfies

$$\frac{\alpha_{TRUE}}{\alpha} \to \frac{1-\log\left(\frac{\alpha}{1-\alpha}\frac{1-\pi_0}{\pi_0}\right)}{1-\alpha\log\left(\frac{\alpha}{1-\alpha}\frac{1-\pi_0}{\pi_0}\right)}. \tag{A.10}$$

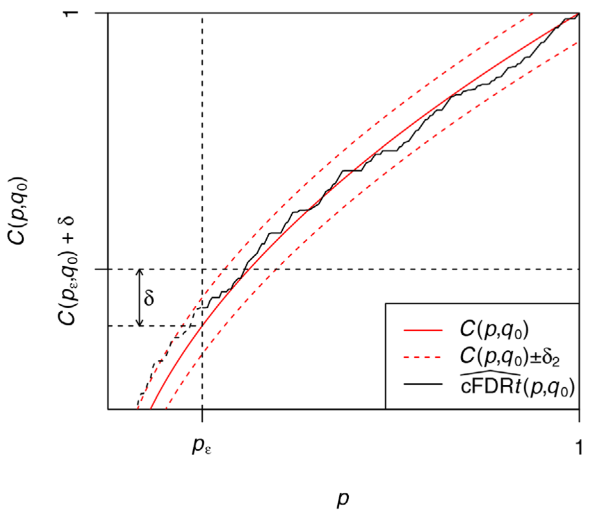

FIGURE A.1 Rejection regions for rejection procedure (9) under assumptions in Section A.2. FDR is not controlled for either of the cFDR-based rejection regions. For reference, the B-H procedure applied to the set of (p, q) pairs with $q \leq q_0$ would reject everything in the dark grey rectangle, which includes all true positives, and this would control FDR at α. The cFDR-based rejection regions reject the same number of true positives, but far more false positives, so FDR control is lost

Corollary A.4. *For $\widehat{cFDR}$, the relative error in FDR (relative to α) can grow arbitrarily large as $\pi_0 \to 1, \alpha \to 0$. For $\widehat{cFDR}^n$, the error can grow arbitrarily large as $\alpha \to 0$, regardless of π_0.*

Proof. Suppose that we have a dataset $S = \{(p_i, q_i)\}, i \in 1 \dots n$ of draws from P, Q under (A.7) and (A.8). Due to assumption (A.7) we have $Pr(P \leq p, Q \leq q | H_0^p) = pq$, and due to (A.8) we have $Pr(P \leq p, Q \leq q | H_1^p) = 1$. Now

$$cFDR(p, q) = Pr(H_0^p | P \leq p, Q \leq q)$$
$$= \frac{\pi_0 pq}{(1 - \pi_0) + \pi_0 pq}.$$

Now

$$Pr(H_0^p | Q \leq q) = \frac{Pr(Q \leq q | H_0^p) Pr(H_0^p)}{Pr(Q \leq q | H_1^p) Pr(H_1^p) + Pr(Q \leq q | H_0^p) Pr(H_0^p)}$$
$$= \frac{\pi_0 q}{(1 - \pi_0) + \pi_0 q}.$$

The estimate $\widehat{cFDR}(p, q)$ is proportional to a consistent estimator of

$$\frac{cFDR(p, q)}{Pr(H_0^p | Q \leq q)} = p \frac{(1 - \pi_0) + \pi_0 q}{(1 - \pi_0) + \pi_0 pq} \tag{A.11}$$

and since $P > 1/2 \Rightarrow H_0^p$, approximation (7) in the main paper is consistent and $\widehat{cFDR}^n(p, q)$ is a generally consistent estimator of $cFDR(p, q)$.

The FDR of the rejection procedure $\widehat{cFDR}(p, q) \leq \alpha$ converges to the FDR of the rejection region $R_\alpha = \{(p, q) : cFDR(p, q)/Pr(H_0^p | Q) \leq q) < \alpha\}$ as $n \to \infty$ (see diagram in Figure A.1). Since this rejection region contains (0,0), all $(1 - \pi_0)n$ non-null hypotheses will be rejected, and the proportion of the total null hypotheses rejected will converge

by the law of large numbers to

$$\int_{R_\alpha} f(P, Q|H_0^p) dpdq = \int_{R_\alpha} dpdq$$
$$= \frac{\alpha}{1-\alpha} \frac{1-\pi_0}{\pi_0} \log\left(\frac{1-\alpha\pi_0}{1-\pi_0}\right), \quad \text{(A.12)}$$

and thus the FDR α_{TRUE} converges to

$$\alpha_{TRUE} \rightarrow \frac{\text{Number of null } (p_i, q_i) \text{ in } R_\alpha}{\text{Total number of } (p_i, q_i) \text{ in } R_\alpha}$$
$$= \frac{\pi_0 n \int_{R_\alpha} dpdq}{(1-\pi_0)n + \pi_0 n \int_{R_\alpha} dpdq}$$
$$= \alpha \frac{\log\left(\frac{1-\alpha\pi_0}{1-\pi_0}\right)}{1-\alpha+\alpha\log\left(\frac{1-\alpha\pi_0}{1-\pi_0}\right)}, \quad \text{(A.13)}$$

which can be written as

$$\log\left(\frac{1-\alpha\pi_0}{1-\pi_0}\right) = \frac{\alpha_{TRUE}(1-\alpha)}{\alpha(1-\alpha_{TRUE})} = OR(\alpha_{TRUE}, \alpha), \quad \text{(A.14)}$$

where OR is the odds ratio. Hence α_{TRUE} can exceed α by an arbitrary degree when pi_0 is close to 1.

The second part of the proof can be shown similarly. The RHS of Equation (A.10) rises as $-\log(\alpha)$ as $\alpha \rightarrow 0$, whatever the value of π_0. □

A.3 | Convergence results

In these appendices, we omit the X from $\widehat{cFDR}_X(p, q)$ and other functions when it is clear. We consider p, q to be in $(0, 1)^2$.

Set $n = |X|$, $F_n(q) = \min(1, |\{i : q_i \leq q, (p_i, q_i) \in X\}|)$, $F_n(p, q) = \min(1, |\{i : p_i \leq p, q_i \leq q, (p_i, q_i \in X)\}|)$, $F(q) = Pr(Q \leq q)$, $F(p, q) = Pr(P \leq p, Q \leq q)$, and

$$C(p, q) = \frac{cFDR(p, q)}{Pr(H_0^p | Q \leq q)} = \frac{pF(q)}{F(p, q)}. \quad \text{(A.15)}$$

We will assume $\partial F(p, q)/\partial p$ exists on $(0, 1)^2$.

We define $\widehat{cFDRt}_X(p, q) = \min_{p' \geq p} \widehat{cFDR}_{X+(p',q)}(p', q)$, and $\overset{n}{\widehat{cFDRt}_X}(p, q)$ similarly for $\overset{n}{\widehat{cFDR}_X}$ ('t' for 'truncated'), so

$$L_X(\alpha) = \left\{(p, q) : \widehat{cFDRt}_X(p, q) \leq \alpha\right\}. \quad \text{(A.16)}$$

The boundary of $L_X(\alpha)$ is continuous and piecewise differentiable.

In this section, we show a series of results relating to convergence of cFDR estimates. We show results relating to convergence of $\widehat{cFDR}$ and $\widehat{cFDRt}$ on a line $q = q_0$, along with convergence of the co-ordinates of L-curves on such lines. We then show slightly weaker results regarding convergence across two-dimensional regions of the unit square.

Theorem A.5. *Suppose that on a line segment $q = q_0$, $p_\gamma < p < 1$, we have $F(p, q) \geq \gamma > 0$ and $F(q_0) > 0$. Then on this segment, $\widehat{cFDR}(p, q)$ converges uniformly to $C(p, q)$ as $n \rightarrow \infty$. If additionally we have $\partial C(p, q)/\partial p \geq 0$, then $\widehat{cFDRt}(p, q)$ converges uniformly to $C(p, q)$ also.*

Proof. Condition on $q = q_0$, and (for the moment) $F_n(q) = m$. Set $\epsilon < \delta$ and let

$$g^-(p,\epsilon) = p\frac{\frac{m}{n}}{F(p,q)+\epsilon} \qquad g^+(p,\epsilon) = p\frac{\frac{m}{n}}{F(p,q)-\epsilon}. \tag{A.17}$$

From the Dvoretzky–Kiefer–Wolfowitz (DKW) inequality, we have

$$\begin{aligned} &Pr\left(F(p,q)-\epsilon \le \frac{F_n(p,q)}{m} \le F(p,q)+\epsilon \middle| q = q_0, F_n(q_0) = m\right) && \ge 1 - e^{-2m\epsilon^2} \\ \Rightarrow\ &Pr\left(g^-(p,\epsilon) \le \widehat{cFDR}(p,q)) \le g^+(p,\epsilon) \middle| q = q_0, F_n(q_0) = m\right) && \ge 1 - e^{-2m\epsilon^2}. \end{aligned} \tag{A.18}$$

If $\partial C(p,q)/\partial p \ge 0$, then (A.18) also holds for $\widehat{cFDRt}$. To see this, note $\widehat{cFDR}(p,q) \ge \widehat{cFDRt}(p,q)$, so if $\widehat{cFDRt}(p,q) \ge g^+(p,\epsilon)$ then $\widehat{cFDR}(p,q) \ge g^+(p,\epsilon)$ also. Now

$$\begin{aligned} \frac{\partial}{\partial p}C(p,q) \ge 0 &\Rightarrow F(p,q) \ge p\frac{\partial}{\partial p}F(p,q) \\ &\Rightarrow F(p,q)+\epsilon \ge p\frac{\partial}{\partial p}F(p,q) \\ &\Rightarrow g^-(p,\epsilon) > 0. \end{aligned}$$

Suppose that for some p we had $\widehat{cFDRt}(p,q_0) \le g^-(p)$. Then either $\widehat{cFDR}(p,q_0) = \widehat{cFDRt}(p,q_0)$ or $\widehat{cFDRt}(p,q_0) = \widehat{cFDR}(p',q_0)$ for some $p' > p$. In the first case $\widehat{cFDR}(p,q_0) \le g^-(p)$, and in the second, $\widehat{cFDR}(p',q_0) = \widehat{cFDRt}(p,q_0) \le g^-(p) \le g^-(p')$; in either case, $\widehat{cFDR}(p,q)$ escapes the bound $g^-(p)$ somewhere. Thus the probability on the LHS of (A.18) can only increase if $\widehat{cFDRt}$ replaces $\widehat{cFDR}$, and $\widehat{cFDRt}(p,q)$ is contained within the bounds $g^-(p), g^+(p)$ with probability at least $1-\exp(-2m\epsilon^2)$.

We now move to remove the condition $F_n(q_0) = m$. Denote the events

$$\begin{aligned} A &: \left\{g^-(p,\epsilon) \le \widehat{cFDR}(p,q) \le g^+(p,\epsilon)\right\} && \text{(A.19)} \\ B &: \{q = q_0\} \end{aligned}$$

and, for some $\epsilon_2 < F(q_0)$

$$C : \left\{p\frac{F(q)-\epsilon_2}{F(p,q)+\epsilon} \le \widehat{cFDR}(p,q) \le p\frac{F(q)+\epsilon_2}{F(p,q)-\epsilon}\right\}. \tag{A.20}$$

Denote by $S(\epsilon_2)$, the set of integers in $[n(F(q_0)-\epsilon_2), n(F(q_0)+\epsilon_2)]$ (and assume n is large enough that $S(\epsilon_2)$ is nonempty). If $m = F_n(q_0) \in S(\epsilon_2)$, the interval in event A is a subinterval of that in event C. Thus

$$\begin{aligned} Pr(C|B) &= \sum_m Pr(C|B, F_n(q_0) = m)Pr(F_n(q_0) = m) \\ &\ge \sum_{m\in S(\epsilon_2)} Pr(C|B, F_n(q_0) = m)Pr(F_n(q_0) = m) \\ &\ge \sum_{m\in S(\epsilon_2)} Pr(A|B, F_n(q_0) = m)Pr(F_n(q_0) = m) \\ &\ge (1 - e^{-2\min\{S(\epsilon_2)\}\epsilon^2})Pr(m \in S(\epsilon_2)) \\ &\ge (1 - e^{-2n(F(q_0)-\epsilon_2)\epsilon^2})(1 - e^{-2n\epsilon_2^2}), \end{aligned} \tag{A.21}$$

where the last inequality comes from the DKW inequality on $F_n(q)$. Since $p \geq p_\epsilon$ and $F(p,q) \geq \gamma$ the widest part of the interval in event C can be made arbitrarily small on the interval $(p_\epsilon, 1)$ and $\widehat{cFDR}(p,q)$ converges uniformly to $C(p,q)$. If $\partial C(p,q)/\partial p \geq 0$, then so does $\widehat{cFDRt}(p,q)$. □

Corollary A.6. *Under the assumptions in Theorem A.5, $\widehat{cFDR}(p,q)$ and $\widehat{cFDRt}(p,q)$ are bound with fixed probability on the line segment $q = q_0$, $p_\gamma < p < 1$ in intervals of width $O(n^{-1/2})$.*

Proof. In inequality (A.21), set

$$\epsilon = \frac{r}{\sqrt{F(q_0) - \epsilon_2}} \qquad \epsilon_2 = \frac{r_2}{\sqrt{n}}. \tag{A.22}$$

Then the RHS is $(1 - \exp(-2r^2))(1 - \exp(-2r_2^2))$, which may be made arbitrarily small by varying r, r_2, and the difference between the upper and lower bounds in event $C|B$ is

$$p\frac{F(q_0) + \epsilon_2}{F(p,q_0) - \epsilon} - p\frac{F(q_0) - \epsilon_2}{F(p,q_0) + \epsilon} = 2p\frac{\sqrt{F(q_0)}r + F(p,q_0)r_2}{F(p,q_0)^2}\frac{1}{\sqrt{n}} + O\left(\frac{1}{n}\right). \tag{A.23}$$

□

Theorem A.7. *Suppose that on a line segment $q = q_0$, $p_\gamma < p < 1$, we have $F(p,q) \geq \gamma > 0$, $F(q_0) > 0$, and $\partial C(p,q)/\partial p \geq \gamma_2 > 0$. Denote by $l(\alpha)$ the value of p at the intersection of the L-curve $L(\alpha)$ with the line $q = q_0$, so*

$$l(\alpha) = \sup\{p : \widehat{cFDRt}(p,q_0) \leq \alpha\}, \tag{A.24}$$

and $c(\alpha)$ the value of p such that $C(p,q_0) = \alpha$ (unique if it exists). For any $\delta > 0$, the function $|l(\alpha) - c(\alpha)|$ converges uniformly to 0 for $\alpha \in [C(p_\epsilon, q_0) + \delta, 1]$.

Proof. Since $C(p,q_0)$ is continuous and increasing on $[p_\epsilon, 1]$, the value $c(\alpha)$ exists for $\alpha \in [C(p_\epsilon,q_0), C(1,q_0)] \supset [C(p_\epsilon,q_0)+\delta, 1]$ by the intermediate value theorem. The function $\widehat{cFDRt}(p,q)$ is continuous and nondecreasing on [0,1] and hence $l(\alpha)$ exists for $\alpha \in [\widehat{cFDRt}(0,q_0), \widehat{cFDRt}(1,q_0)] = [0,1]$.

Given arbitrarily small positive ϵ_3, $\delta_2 < \delta$ choose n large enough that $\widehat{cFDRt}(p,q_0)$ is contained in $[C(p,q_0) - \delta_2, C(p,q_0) + \delta_2]$ for $p \in [p_\epsilon, 1]$ with probability at least $1 - \epsilon_3$. Then with probability $\geq 1 - \epsilon_3$, whenever the curve $\widehat{cFDRt}(p,q)$ is in the region bounded by the rectangle $p_\epsilon \leq p \leq 1$, $C(p_\epsilon,q_0) + \delta \leq q \leq 1$, it is bounded by the curves $C(p,q_0) - \delta_2)$, $C(p,q_0) + \delta_2$. The distance between the two curves in the q-direction is at most $2\gamma_2\delta_2$. Thus, if for some $\alpha \in [C(p_\epsilon,q_0) + \delta, 1]$, we have $|l(\alpha) - c(\alpha)| > \gamma_2\delta_2$, the curve $\widehat{cFDRt}(p,q_0)$ must escape the region bounded the curves $C(p,q_0) - \delta_2)$, $C(p,q_0) + \delta_2$.

So with probability at least $1 - \epsilon_3$, we have

$$\forall \alpha \in [C(p_\epsilon,q_0) + \delta, 1] : |l(\alpha) - c(\alpha)| \leq \gamma_2\delta_2, \tag{A.25}$$

which proves the statement. This is illustrated in Figure A.2. □

We now proceed to the proof of Theorem 3.3, restated here:

Theorem 3.3. *Let R be the region of the unit square for which $F(p,q) \geq \gamma > 0$ and $F(q) > 0$. Then on R, $\widehat{cFDR}(p,q)$ converges uniformly to $C(p,q)$, and if $\partial C(p,q)/\partial p \geq 0$, then so does $\widehat{cFDRt}(p,q)$.*

Proof. We proceed very similarly to Theorem A.5. We employ a result from Kiefer (1961) that for any $\epsilon > 0$

$$Pr\left(\sup|F_n(p,q) - F(p,q)| \geq \frac{r}{\sqrt{n}}\right) \leq c(\epsilon)e^{(2-\epsilon)r^2} \tag{A.26}$$

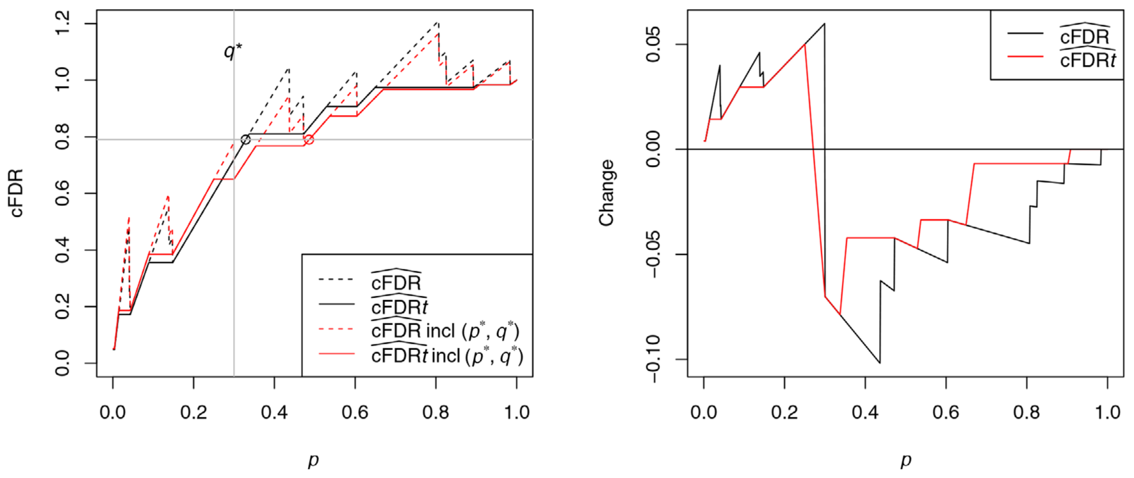

FIGURE A.2 Convergence of intersections of L-curves with a line $q = q_0$. Functions $\widehat{cFDRt}(p, q_0)$, $C(p, q_0)$ and $C(p, q_0) \pm \delta_2$ are shown. The vertical distance between dashed red lines is $2\delta_2$, and since $\partial C(p, q0)/\partial p \geq \gamma_2$ the horizontal distance is at most $2\delta_2\gamma_2$. We must restrict the proof to $\alpha > C(p, q_0) + \delta$ because we cannot assert the behaviour of $\widehat{cFDRt}(p, q)$ left of the line $p = p_\epsilon$

from which, wherever $F(p, q) > f_{min} > \frac{r}{\sqrt{n}}$

$$Pr\left(\frac{1}{F(p,q)+\frac{r}{\sqrt{n}}} \leq \frac{1}{F_n(p,q)} \leq \frac{1}{F(p,q)-\frac{r}{\sqrt{n}}}\right) \geq 1 - c(\epsilon)e^{(2-\epsilon)r^2}$$

$$Pr\left(\frac{p\frac{F_n(q)}{n}}{F(p,q)+\frac{r}{\sqrt{n}}} \leq \widehat{cFDR}(p,q) \leq \frac{p\frac{F_n(q)}{n}}{F(p,q)-\frac{r}{\sqrt{n}}}\right) \geq 1 - c(\epsilon)e^{(2-\epsilon)r^2}. \tag{A.27}$$

The values $F_n(p, q)$ and $F_n(q)$ are dependent. However, given some $r_2 > 0$, we have for all q (by the DKW inequality)

$$Pr\left(F(q) - \frac{r_2}{\sqrt{n}} \leq \frac{F_n(q)}{n} \leq F(q) + \frac{r_2}{\sqrt{n}}\right) \geq 1 - 2e^{-2r_2^2}. \tag{A.28}$$

Denoting the event in (A.27) by A, the event in (A.28) by B, and C as

$$\frac{p(F(q) - \frac{r_2}{\sqrt{n}})}{F(p,q)+\frac{r}{\sqrt{n}}} \leq \widehat{cFDR}(p,q) \leq \frac{p(F(q) + \frac{r_2}{\sqrt{n}})}{F(p,q)-\frac{r}{\sqrt{n}}} \tag{A.29}$$

we have, since the interval in A is a subinterval of that in C when conditioning on B:

$$Pr(C) = Pr(C|B)Pr(B) + Pr(C|\neg B)Pr(\neg B) \tag{A.30}$$

$$\geq Pr(C|B)(1 - 2e^{-2r_2^2}) \tag{A.31}$$

$$\geq Pr(A|B)(1 - 2e^{-2r_2^2}) \tag{A.32}$$

$$\geq (1 - c(\epsilon)e^{(2-\epsilon)r^2})(1 - 2e^{-2r_2^2}). \tag{A.33}$$

As before, this bound also holds for $\widehat{cFDRt}$ as long as $\partial C(p, q)/\partial p > 0$. □

Corollary A.8. *Under the assumptions in 3.3, $\widehat{cFDR}$ and $\widehat{cFDRt}$ are bound with fixed probability in R in intervals of width* $O(n^{-1/2})$

Proof. The difference between the upper and lower bounds in (A.29) is

$$\frac{2p(rF(q)+r_2F(p,q))}{F(p,q)^2}\frac{1}{\sqrt{n}}+O\left(\frac{1}{\sqrt{n}^3}\right). \tag{A.34}$$

□

Our final result describes errors on v-values. Given an L-region $L(\alpha)$, we define the M-region as the 'expected' L-region:

$$M(\alpha)=\{(p,q):C(p,q)\leq\alpha\}, \tag{A.35}$$

and the 'error' on the v-value $v=\int_{L(\alpha)}f_0(p,q)dpdq$ as

$$|\Delta v|=\left|\int_{L(\alpha)}f_0(p,q)dpdq-\int_{M(\alpha)}f_0(p,q)dpdq\right|. \tag{A.36}$$

We now are now in a position to prove Theorem 3.4.

Theorem 3.4. *Define R as in Theorem 3.3, and further assume that $f_0(p,q)=f(P=p,Q=q|H_0^P)$ is known and on R we have $\partial C(p,q)/\partial p\geq\gamma_2$. Write $R^c=[0,1]^2\setminus R$. Then the maximum error on any v-value is*

$$\int_{R^c}f_0(p,q)dpdq+O\left(\frac{1}{\sqrt{n}}\right). \tag{A.37}$$

Proof. Using Theorem 3.3, bound $\widehat{cFDRt}(p,q)$ between $C(p,q)-\delta$, $C(p,q)+\delta$ with probability $\geq 1-\epsilon_3$, where $\delta=O(1/\sqrt{n})$.

Since $F(p,q)$ is non-decreasing with p, we can describe $R=\{(p,q):F(p,q)\geq\gamma\}$ as the union of line segments $q=q_0$, $p_\epsilon(q_0)\leq p\leq 1$. We now define R_1 as the union of all line segments $q=q_0$, $p_\epsilon(q_0)+\delta\gamma_2\leq p\leq 1$; that is, R with the leftmost border shifted $\delta\gamma_2$ to the right.

We show the result by firstly noting that if an L-curve intersects a line segment $q=q_0$ at $l(\alpha)>p_\epsilon(q_0)+\delta\gamma_2$, and we have that $|l(\alpha)-c(\alpha)|>\delta\gamma_2$ (where $c(\alpha)$ is the intersection of the border of $M(\alpha)$ with $q=q_0$), then event C (Equation (A.29)) must have occurred in R, by the same argument as for Theorem A.7. Thus with probability at least $1-\epsilon_3$, every segment of a right-most border of an L-region $L(\alpha)$ in R_1 is at a horizontal distance from the corresponding rightmost-border of $M(\alpha)$ of at most $\delta\gamma_2$

We now write

$$\begin{aligned}\Delta v=&\left(\int_{L(\alpha)\cap R^c}f_0(p,q)dpdq-\int_{M(\alpha)\cap R^c}f_0(p,q)dpdq\right)\\&+\left(\int_{L(\alpha)\cap(R\setminus R_1)}f_0(p,q)dpdq-\int_{M(\alpha)\cap(R\setminus R_1)}f_0(p,q)dpdq\right)\\&+\left(\int_{L(\alpha)\cap R_1}f_0(p,q)dpdq-\int_{M(\alpha)\cap R_1}f_0(p,q)dpdq\right).\end{aligned} \tag{A.38}$$

The first term is at most $\int_{R^c}f_0(p,q)dpdq$. The region $R\setminus R_1$ has constant width $\delta\gamma_2$, and since f_0 only varies with q, hence the second term is at most $\int_{R\setminus R_1}f_0(p,q)dpdq=\delta\gamma_2=O(n^{-1/2})$. Within R_1, if the horizontal separation between curves

at the rightmost border of $L(\alpha)$ and $M(\alpha)$ is greater than $\delta\gamma_2$, then C has occurred, so this can happen with probability at most ϵ_3. Thus with probability $1 - \epsilon_3$, the third term is also bounded by $\delta\gamma_2 = O(n^{-1/2})$, establishing the result. □

A.4 | Influence of a single point

Intuitively, adding a single point to a map defined by n other points should have a small effect on that map, and hence on the resultant v-values. We show the following:

Theorem 3.5. *Suppose we add a point (p^*, q^*) to a set of n points (p_i, q_i), considered as realisations of P, Q, and conditions are satisfied for convergence of v-values as above. Let $\Delta v(L(\alpha))$ be the shift in a v-value corresponding to an L-curve $L(\alpha)$ after adding (p^*, q^*). Then*

$$E_{\alpha\sim U(0,1)}(|\Delta v(L(\alpha))|) = O\left(\frac{1}{n^2}.\right). \tag{A.39}$$

Proof. Consider the profile of $\widehat{cFDRt}(p,q)$ on a line $q = q_0$, and how this changes with the addition of (p^*, q^*). The functions $F_n(q), F_n(p,q)$ will be taken to be with respect to the n points (p_i, q_i) but not (p^*, q^*).

For $q_0 < q^*$, the addition of (p^*, q^*) changes neither $F_n(q_0)$ nor $F_n(p, q_0)$, so on lines $q = q_0 < q^*$ the profile of $\widehat{cFDRt}$ will remain the same.

Denote

$$c^+(p) = p\frac{F_n(q)+1}{F_n(p,q)} \qquad c^-(p) = p\frac{F_n(q)+1}{F_n(p,q)+1}. \tag{A.40}$$

For $q_0 > q^*$, $p < p^*$, the value of $\widehat{cFDR}(p, q_0)$ will increase by

$$c^+(p) - p\frac{F_n(q)}{F_n(p,q)} = \frac{p}{F_n(p,q_0)}, \tag{A.41}$$

and for $q_0 > q^*$, $p > p^*$, it will decrease by

$$p\frac{F_n(q)}{F_n(p,q)} - c^-(p) = p\frac{F_n(q_0) - F_n(p,q_0)}{F_n(p,q_0)(F_n(p,q_0)+1)}. \tag{A.42}$$

In either case, $\widehat{cFDR}(p, q_0)$ changes by $O(\frac{1}{n^2})$. The behaviour of $\widehat{cFDRt}$ is a little more complex. If we define $c_t^+(p)$ and $c_t^-(p)$ analogously to $\widehat{cFDRt}(p, q_0)$, then for $p > p^*$ $\widehat{cFDRt}(p, q_0)$ shifts to $c_t^-(p)$, and for $p < p^*$ it shifts to $\min(c_t^+(p), c_t^-(p^*)$ (see example in Figure A.3).

We can show that the absolute difference in $\widehat{cFDRt}(p, q_0)$ is always less than the absolute difference in $\widehat{cFDR}(p, q_0)$ after adding (p^*, q^*). Denote these differences $\Delta\widehat{cFDR}(p, q_0)$ and $\Delta\widehat{cFDRt}(p, q_0)$. Since $\widehat{cFDRt}(p, q_0)$ always shifts to between $c_t^-(p)$ and $c_t^+(p)$, it suffices to show that

$$c_t^+(p) - \widehat{cFDRt}(p, q_0) \le \Delta\widehat{cFDR}(p, q_0) \tag{A.43}$$

$$\widehat{cFDRt}(p, q_0) - c_t^-(p) \le \Delta\widehat{cFDR}(p, q_0). \tag{A.44}$$

Inequality (A.43) follows from the observation that $c^+(p) \propto \widehat{cFDR}(p, q_0)$, so order relations between $\widehat{cFDR}(p, q_0)$ and $c^+(p, q_0)$ are preserved. Thus

$$|\Delta\widehat{cFDRt}(p, q_0)| = \min_{p'\ge p} \Delta|\widehat{cFDR}(p', q_0)| \le |\Delta\widehat{cFDR}(p', q_0)|. \tag{A.45}$$

Order relations are not preserved between $\widehat{cFDR}(p, q_0)$ and $c^-(p)$, but the denominators increment at the same values of p. The functions $\widehat{cFDR}(p, q_0)$ and $c^-(p)$ both rise linearly in p between successive increment points p_a, p_d of $F_n(p, q_0)$,

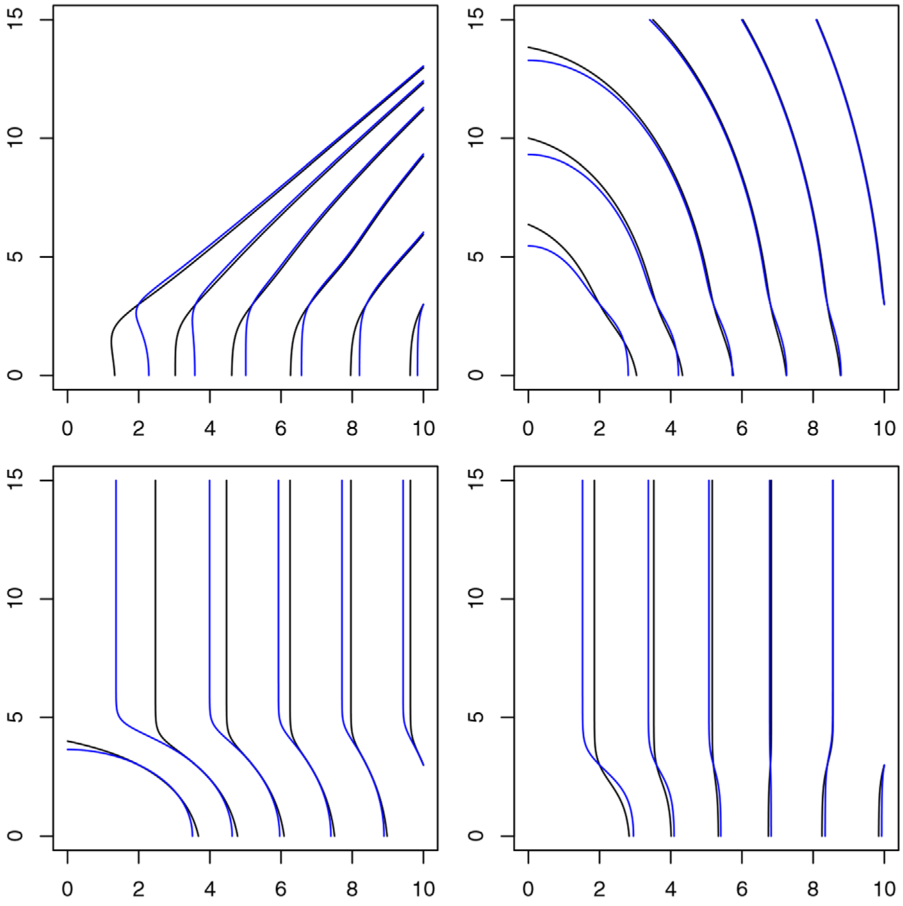

FIGURE A.3 Behaviour of $\widehat{cFDR}$ and $\widehat{cFDRt}$ on a line $q = q_0$ after adding a point (p^*, q^*) to a set of n points (n is considerably smaller in this example than in Figure A.2). In the left panel, curves of $\widehat{cFDR}$ and $\widehat{cFDRt}$ before and after adding (p^*, q^*) are shown. Adding (p^*, q^*) may have a substantial impact on the intersection of an L-curve with $q = q_0$, such as that in the horizontal line: the black and red points show the intersection points of a curve before and after adding (p^*, q^*). However, the average effect across all curves is limited to the integral of the difference between the black and red lines, which is $O(1/n^2)$. The right panel demonstrates that $|\Delta\widehat{cFDRt}(p,q)| \leq |\Delta\widehat{cFDR}(p,q)|$

with $\widehat{cFDR}(p, q_0)$ having the higher gradient, since

$$\frac{F_n(q)}{F_n(p,q)} > \frac{F_n(q)+1}{F_n(p,q)+1}. \tag{A.46}$$

At p_d, both functions are discontinuous and drop in value. On (p_a, p_d), the values of $c_t^-(p)$ and $\widehat{cFDRt}(p, q_0)$ are either equal to $c^-(p)$, $\widehat{cFDR}(p, q_0)$, or 'censored' at some values $c^-(p')$, $\widehat{cFDR}(p', q_0)$ with $p' > p$ (see the right-hand part of Figure A.3 for an example of this). We note that $c^-(p) > c^-(p'), p' > p \Rightarrow \widehat{cFDR}(p, q_0) > \widehat{cFDR}(p, q_0)$, so the first point at which $c_t^-(p)$ is censored on (p_1, p_2) is further right than the first point at which $\widehat{cFDRt}(p, q_0)$ is censored. Denote the leftmost point at which $\widehat{cFDRt}(p, q_0)$ is censored as p_b and the leftmost point at which $c_t^-(p)$ is censored as p_c, so $p_a \leq p_b \leq p_c \leq p_d$.

On (p_a, p_b), where neither are censored, $\widehat{cFDR}(p, q_0) - c^+(p) = \widehat{cFDRt}(p, q_0) - c_t^-(p)$ and $\Delta\widehat{cFDR}(p, q_0) = \Delta\widehat{cFDR}(p, q_0)$. On (p_b, p_c), when only $\widehat{cFDRt}(p, q_0)$ is censored, $\widehat{cFDRt}(p, q_0) - c_t^-(p) = \widehat{cFDR}(p_b, q_0) - c^-(p) < \widehat{cFDR}(p, q_0) - c^-(p)$, so $\Delta\widehat{cFDR}(p, q_0) \leq \Delta\widehat{cFDR}(p, q_0)$. On (p_c, p_d), we have $\widehat{cFDRt}(p, q_0) - c_t^-(p) = \widehat{cFDR}(p_b, q_0) - c^-(p_c) \leq \widehat{cFDR}(p_c, q_0) - c^-(p_c) \leq \widehat{cFDR}(p, q_0) - c^-(p)$, so again, $\Delta\widehat{cFDRt}(p, q_0) \leq \Delta\widehat{cFDR}(p, q_0)$. Thus, for all p,

$$|\Delta\widehat{cFDRt}(p, q_0)| \leq |\Delta\widehat{cFDR}(p, q_0)| = O\left(\frac{1}{n^2}\right), \tag{A.47}$$

where the multiplicative factor in $O(1/n^2)$ is independent of q_0. This inequality is demonstrated in the right panel of Figure A.3.

Denote by l_α the value of p at the intersection of an L-curve corresponding to $\widehat{cFDRt}(p,q) \leq \alpha$ with the line $q = q_0$. We have $l_\alpha = \max\{p : \widehat{cFDRt}(p,q_0) = \alpha\}$. The value l_α may shift substantially when adding p^*, q^*, as shown in Figure A.3

However, the effect is small on average. The plot of the function $l(\alpha)$ before and after adding (p^*, q^*) is identical to the plot of the function of p given by $\widehat{cFDRt}(p,q_0)$ before and after adding (p^*, q^*) rotated by $\pi/2$. The average difference in movement of l_α is the integral of the difference in l_α with and without (p^*, q^*). However, this is simply the area between the two curves, which is invariant under rotating $\pi/2$. Hence

$$\int_0^1 \Delta l_\alpha d\alpha = \int_0^1 \Delta\widehat{cFDRt}(p,q_0)dp = O\left(\frac{1}{n^2}\right). \tag{A.48}$$

Denote the region $L(\alpha) : \widehat{cFDRt}(p,q) \leq \alpha$ and the co-ordinates of its rightmost border (L-curve) $(q, l_\alpha(q))$, $q \in (0,1)$. Then, denoting the indicator function by I

$$\begin{aligned} v(L(\alpha)) &= \int_0^1 \int_0^1 I((p,q) \in L(\alpha)) f_0(p,q) dp\, dq \\ &= \int_0^1 \int_0^1 I((p,q) \in L(\alpha)) f_0^q(q) dp\, dq \\ &= \int_0^1 f_0^q(q) \int_0^1 I((p,q) \in L(\alpha)) dp\, dq \\ &= \int_0^1 f_0^q(q) l_\alpha(q) dq, \end{aligned} \tag{A.49}$$

and the average error in v-values $v(L(\alpha))$ over $\alpha \sim U(0,1)$ is

$$\begin{aligned} E_{\alpha\sim U(0,1)}(|\Delta v(L(\alpha))|) &= \int_0^1 \Delta v(L(\alpha)) d\alpha \\ &= \int_0^1 \int_0^1 f_0^q(q) \Delta l_\alpha(q) dq\, d\alpha \\ &= \int_0^1 f_0^q(q) \int_0^1 \Delta l_\alpha(q)\, d\alpha dq \\ &= O\left(\frac{1}{n^2}\right) \int_0^1 f_0^q(q) dq \\ &= O\left(\frac{1}{n^2}\right) \end{aligned} \tag{A.50}$$

as required. □

A.5 | Asymptotic equivalence of PDF- and CDF- based L-regions

We show in this section that under a fairly common condition L-regions based on the PDF of p, q are similar to L-regions based on the CDF. In this section, we generally work on the Z-scale rather than the p-value scale for convenience.

Denote a 'fast-decreasing' function as a function g such that for each $\epsilon_1, \epsilon_2 > 0$, there exists δ such that for all X, Y of distance at least δ from the origin, we have

$$\iint\limits_{\substack{x \leq X, y \leq Y \\ (x-X)^2 + (y-Y)^2 \leq \epsilon_1^2}} g(x,y) dx dy > (1-\epsilon_2) \iint\limits_{x \leq X, y \leq Y} g(x,y) dx dy \tag{A.51}$$

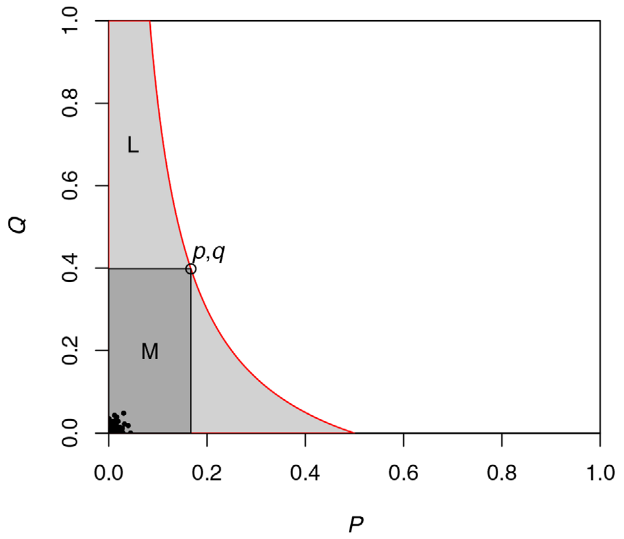

FIGURE A.4 These plots show contours of CDF- based and PDF-based L-regions for a range of distributions of P, Q. Plots are on the Z-score scale (e.g., rejection regions in terms of Z_P, Z_Q). The distributions are parameterised in terms of the mixture-Gaussian distribution detailed in Supporting Information, Section 1.4.1; parameters $(\pi_0, \pi_1, \pi_2, \tau_1, \tau_2, \sigma_1, \sigma_2)$ were (0.7,0.1,0.1,2,3,1.5,1.5), (0.7,0.1,0.1,2,2,3,3), (0.99,0.0005,0.0003,2,3,4,3) and (0.7,0.1,0.05,3,2,2,2), respectively. Curves are generated passing through the points $Z_P, Z_Q = (3, 2 \ldots 6)$, with curves further to the right corresponding to smaller α. As α gets smaller, contours $F_0(x,y)/F(x,y) = \alpha$ (black lines) become closer to contours of $f_0(x,y)/f(x,y) = \alpha$ (blue lines) under reasonably general circumstances

so for $x \le X, y \le Y$, the function g falls off rapidly enough as x, y decrease that we can disregard its value except when it is close to X, Y.

We show the following:

Theorem A.9. *Denote* $c(x,y) = f_0(x,y)/f(x,y)$ *and* $C(x,y) = F_0(x,y)/F(x,y)$. *Given a region of the* $(-,-)$ *quadrant* $A_\epsilon = (-\infty, 0] \times (I_1 - \epsilon, I_2 + \epsilon)$ *(where* $\epsilon > 0$ *is arbitrarily small), suppose that for* $x, y \in A_\epsilon$ *and for sufficiently small* α *we have*

1. f_0 *and* f *are fast-decreasing continuous positive functions,*
2. *Along horizontal rays in A,* $c(x,y)$ *satisfies* $\partial^2 \log(c(x,y))/\partial x^2 > 0$ *and*
3. *The contour* $c(x,y) = \alpha$ *is continuous and bounded, and the rightmost bound increases to* ∞ *as* $\alpha \to 0$.

Then for each $\epsilon_3 > 0$, *there exists an* ϵ_1 *as above and an* α_1 *such that whenever* $\alpha < \alpha_1$, *there is a contour of* $C(x,y)$ *is never further than* ϵ_3 *from the contour* $c(x,y) = \alpha$ *in the region* A_0.

Proof. Set R_3 as the region defined by the union of all circles of radius ϵ_3 with centres on points $y, l_\alpha(y)$. Choose $\epsilon_1 = \epsilon_3/2$ (supposing that $\epsilon_1 < \epsilon$), and define R_1 similarly to R_3 with radii ϵ_1. Let α^+ be the minimum value of f_0/f on the rightmost border of R_1, and α^- the maximum value on the leftmost border so $\alpha^+ > \alpha > \alpha^-$.

Condition 2 implies that for fixed y

$$\frac{d}{dx}\left(\frac{c(x+\epsilon_1, y)}{c(x,y)}\right) < 0. \tag{A.52}$$

Since the horizontal distance between the rightmost border of R_3 and the curve is at least $2\epsilon_1$ and similarly from the leftmost border of R_3, the values $\alpha^+ - \alpha$, $\alpha - \alpha^-$ must increase for fixed ϵ_1 as we move left. Thus, for some fixed $\epsilon_2 > 0$, choose δ_2 large enough that $\alpha^+/\alpha^- > 1/(1-\epsilon_2)^2$ and larger than the δ corresponding to ϵ_1, ϵ_2 by assumption, and α_1 large enough that the contour $c(x,y)$ is entirely left of the line $x = -\delta_2$.

Let X, Y be a point in A_0 to the right of R_3, so a circle of radius ϵ_1 centred at X, Y is in A_ϵ but does not intersect R_1. Thus across such a circle, the value of $c(x,y)$ is at least α^+. Similarly, across a circle of radius ϵ_1 centred to the left of R_3, the value of $c(x,y)$ is at most α^-

For x, y to the right of R_3, denote by H the circle of radius ϵ_1 centred at x, y. Now by the fast-decreasing property of f_0 and f, we have

$$F_0(x,y) > \int_H f_0(x,y)dxdy > \alpha^+ \int_H f(x,y)dxdy > \alpha^+(1-\epsilon_2)F(x,y) \tag{A.53}$$

so $C(x, y) > \alpha^+(1 - \epsilon_2)$. Similarly for x, y to the left of R_3, we have $C(x, y) < \alpha^-/(1 - \epsilon_2)$. By our choice of α_1, we have $\alpha^+(1 - \epsilon_2) > \alpha^-/(1 - \epsilon_2)$, so any contour of $C(x, y)$ at a level between these values must pass within R_3 through A_0. □

Contours of F_0/F correspond to contours of $cFDR$, and contours of f_0/f correspond to contours of $Pr(H_0^p|P = p, Q = q)$. Theorem A.9 has obvious analogies in other quadrants, and for the p-value rather than z-score scale.

The conditions in the theorem may seem restrictive, but they are satisfied by many distributions; for instance, when f_0 and f are mixture Gaussian, and f dominates f_0 as $|x| \to \infty$. Figure A.4 shows the similarity of a range of shapes of contours of C and c.
